# EnsembleDesign: messenger RNA design minimizing ensemble free energy via probabilistic lattice parsing

**DOI:** 10.1093/bioinformatics/btaf245

**Published:** 2025-07-15

**Authors:** Ning Dai, Tianshuo Zhou, Wei Yu Tang, David H Mathews, Liang Huang

**Affiliations:** School of Electrical Engineering and Computer Science, Oregon State University, Corvallis, OR 97330, United States; School of Electrical Engineering and Computer Science, Oregon State University, Corvallis, OR 97330, United States; School of Electrical Engineering and Computer Science, Oregon State University, Corvallis, OR 97330, United States; Department of Biochemistry and Biophysics, University of Rochester Medical Center, Rochester, NY 14642, United States; Center for RNA Biology, University of Rochester Medical Center, Rochester, NY 14642, United States; Department of Biostatistics and Computational Biology, University of Rochester Medical Center, Rochester, NY 14642, United States; School of Electrical Engineering and Computer Science, Oregon State University, Corvallis, OR 97330, United States; Department of Biochemistry and Biophysics, Oregon State University, Corvallis, OR 97330, United States

## Abstract

**Motivation:**

The task of designing optimized messenger RNA (mRNA) sequences has received much attention in recent years, thanks to breakthroughs in mRNA vaccines during the COVID-19 pandemic. Because most previous work aimed to minimize the minimum free energy (MFE) of the mRNA in order to improve stability and protein expression, which only considers one particular structure per mRNA sequence, millions of alternative conformations in equilibrium are neglected. More importantly, we prefer an mRNA to populate multiple stable structures and be flexible among them during translation when the ribosome unwinds it.

**Results:**

Therefore, we consider a new objective to minimize the ensemble free energy of an mRNA, which includes all possible structures in its Boltzmann ensemble. However, this new problem is much harder to solve than the original MFE optimization. To address the increased complexity of this problem, we introduce EnsembleDesign, a novel algorithm that employs continuous relaxation to optimize the expected ensemble free energy over a distribution of candidate sequences. EnsembleDesign extends both the lattice representation of the design space and the dynamic programming algorithm from LinearDesign to their probabilistic counterparts. Our algorithm consistently outperforms LinearDesign in terms of ensemble free energy, especially on long sequences. Interestingly, as byproducts, our designs also enjoy lower average unpaired probabilities (which correlates with degradation) and flatter Boltzmann ensembles (more flexibility between conformations).

**Availability and implementation:**

Our code is available on: https://github.com/LinearFold/EnsembleDesign.

## 1 Introduction

RNA is of utmost importance in life because it plays both the *informational* role, where messenger RNAs (mRNAs) pass genetic information from DNA to proteins, as well as the *functional* roles where non-coding RNAs facilitate protein translation, catalyze reactions, and regulate gene expression. In addition, the public became more aware of RNA because COVID-19 was caused by the RNA virus SARS-CoV-2 (Wu *et al.* 2020), which was then partially contained by mRNA vaccines (Polack *et al.* 2020, Baden *et al.* 2021). The breakthrough behind the vaccines won the 2023 Nobel Prize.

For a given protein, the task of mRNA design aims to find, among all valid mRNA sequences encoding that protein, a handful of optimized sequences under some metric. One such metric that has gained popularity is *thermodynamic stability*, which has been shown to correlate with half-life, protein expression, and immunogenicity (Mauger *et al.* 2019, Wayment-Steele *et al.* 2021, Leppek *et al.* 2022, Zhang *et al.* 2023). More formally, this optimization aims to find the mRNA sequence with the lowest minimum free energy (MFE). Although the design space is exponentially large due to synonymous codons, this problem can be solved by efficient dynamic programs (DP) (Cohen and Skiena 2003, Terai *et al.* 2016, Zhang *et al.* 2023). In particular, LinearDesign (Zhang *et al.* 2023) provides a conceptually simple solution by borrowing the concepts of lattice and lattice parsing from computational linguistics to compactly represent the design space and to efficiently score the energy model over that space, respectively.

However, the above MFE metric only considers a single (MFE) structure per mRNA sequence, while in reality, there are exponentially many alternative conformations in equilibrium. More importantly, we prefer mRNA to be conformationally “flexible” during translation when the ribosome unwinds it. For example, mRNA secondary structures are known to contribute to ribosome stalling in frameshifting (Yan *et al.* 2015) and no-go mRNA decay (Doma and Parker 2006). In other words, we want smoother energy landscapes, while optimizing for MFE often produces steep ones.

To mitigate these potential issues and to consider the Boltzmann ensemble of all secondary structures, here we design mRNAs that minimize the *ensemble free energy* (EFE). Sequences chosen under this new metric will populate multiple low free energy structures, which maximizes base-pairing probabilities and reduces the thermodynamic penalty for the mRNA sequence to switch between alternative structures as the ribosome opens base pairs during translation. This “by-product” of maximizing base-pairing probabilities also leads to minimizing the *average unpaired probabilities* (AUP) (Wayment-Steele *et al.* 2021) which has been shown to correlate with degradation (Leppek *et al.* 2022). A parallel work (Krueger and Ward 2024) also minimizes EFE but with a very different method; see Section 7.

Unfortunately, this new objective, being a minimization over a summation (of all structures), is much harder to optimize than the MFE one (a minimization over a minimization). DP is not feasible for such problems and it is likely NP-hard (Cormen *et al.* 2022). To tackle this challenge, we introduce *EnsembleDesign*, a new algorithm that employs continuous relaxation by optimizing the expected EFE over a distribution of candidate sequences. We generalize the lattice representation in LinearDesign to a probabilistic lattice and extend its lattice parsing algorithm to compute the expected partition function over this relaxed design space.

We evaluate EnsembleDesign on 20 UniProt protein sequences as well as the SARS-CoV-2 spike protein. Across all cases, EnsembleDesign consistently outperforms LinearDesign in reducing EFE, with especially strong improvements on longer sequences. More interestingly, as byproducts expected above, our algorithm indeed reduces AUP as well as flattens energy landscapes and Boltzmann ensembles which suggest more conformational flexibility during translation.

## 2 Background: mRNA design for minimum free energy

We briefly review the mRNA design problem, the classical MFE objective, and the LinearDesign algorithm for this problem.

**Figure 1.**
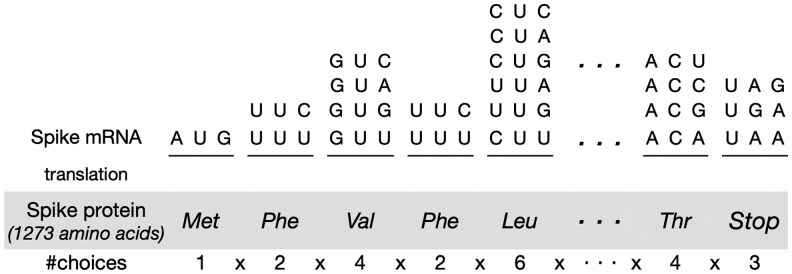
Illustration of the exponentially large mRNA design space. Here, the target protein has |p|=1273 amino acids, which can be encoded by $|X(p)|\approx 2.4\times 10^{632}$ mRNA sequences.

The input to the mRNA design problem is a target protein sequence $p=(p_{1},p_{2},\ldots ,p_{n})$ where each $p_{i}$ is an amino acid residue. We denote the set X(p) to be the set of all valid mRNA sequences that translate to protein p (i.e. the design space). Each mRNA sequence $x=(x_{1},x_{2},..,x_{3n})$ in X(p) contains *n* codons, where each codon is the three consecutive nucleotides $x_{\left[3i-2:3i\right]}$ that translates to amino acid $p_{i}(i=1\ldots n)$. Because most amino acids can be encoded by multiple codons (due to the redundancy in the genetic code), the mRNA design space grows exponentially with protein length, and is prohibitively large. For example, there are $2.4\times 10^{632}$ mRNA sequences for the SARS-CoV-2 spike protein with 1273 amino acids (see Fig. 1).

### 2.1 Classical objective: minimum free energy

Among all possible mRNAs in X(p), which one should we prefer? A standard objective is to find the thermodynamically most stable one, because thermodynamic stability has been shown to improve half-life, protein expression, and immunogenicity (Mauger *et al.* 2019, Zhang *et al.* 2023). We denote MFE(x) to be the minimum folding free energy change of mRNA x:


$$\text{MFE}(x)\overset{\Delta }{=}\underset{y\in Y(x)}{\min}\Delta G^{{}^{\circ}}(x,y),$$

where Y(x) is the set of all possible secondary structures for x, and $\Delta G^{{}^{\circ}}(x,y)$ is the free energy change of mRNA x and structure y. This function is also known as the RNA folding problem (Do *et al.* 2006, Lorenz *et al.* 2011, Huang *et al.* 2019), where we want to find the most stable structure for the input RNA x. Based on this function, we can express the optimization problem the following, which is a minimization over a minimization:


(1) $$\underset{x\in X(p)}{\min}\text{MFE}(x)=\underset{x\in X(p)}{\min}\underset{y\in Y(x)}{\min}\Delta G^{{}^{\circ}}(x,y).$$

### 2.2 LinearDesign: lattice and lattice parsing

**Figure 2.**
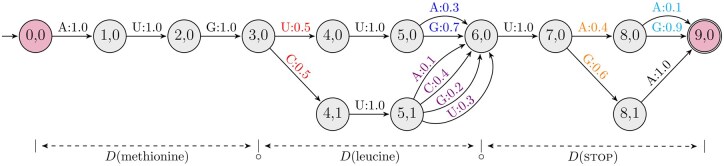
A probabilistic lattice is a probabilistic pDFA and represents a distribution over mRNA sequences for a given protein. Each node corresponds to a state in the DFA, with outgoing edges labeled by nucleotides and their associated probabilities. For each node, the probabilities of outgoing edges (sharing the same color) sum to 1, ensuring proper distribution.

The LinearDesign algorithm (Zhang *et al.* 2023) provided a conceptually simple and practically efficient solution to the above optimization problem, by borrowing two key ideas from computational linguistics. The first idea is to compactly represent the exponentially large design space X(p) by a “lattice”, or more formally a deterministic finite-state automata (DFA) (Fig. 2). The second idea is to use “lattice parsing” (Bar-Hillel *et al.* 1961) to efficiently score the energy function $\Delta G^{{}^{\circ}}(\cdot ,\cdot )$ over the whole lattice, thanks to the decomposability of the energy function onto individual loops. Lattice parsing is a generalization of single-sequence folding where the latter is special case where the lattice is a linear chain.

## 3 New objective: minimum ensemble free energy

While the MFE objective focuses on identifying a single most stable conformation, it is more beneficial to consider the stability of the mRNA across *all* possible conformations. Since an RNA sequence can fold into exponentially many structures within the Boltzmann ensemble at equilibrium, optimizing for overall structural stability can be modeled as minimizing the EFE:


$$\Delta G_{\text{ens}}^{{}^{\circ}}(x)\overset{\Delta }{=}-RT\;\text{log}\;Q(x),$$

where *R* is the universal gas constant, *T* is the absolute temperature, and Q(x) is the *partition function* for x:


$$Q(x)\overset{\Delta }{=}\sum_{y\in Y(x)}e^{-\Delta G(x,y)/RT},$$

where Y(x) represents the set of all feasible secondary structures for RNA x, and ΔG(x,y) is the free energy change associated with structure y. Formally, we can express the new optimization problem as:


(2) $$\underset{x\in X(p)}{\min}\Delta G_{\text{ens}}^{{}^{\circ}}(x)=\underset{x\in X(p)}{\min}-RT\;\text{log}\;\sum_{y\in Y(x)}e^{-\Delta G(x,y)/RT}$$

Using this new objective, we can find mRNA sequences with multiple low free energy change conformations, which could lead to structurally flexible mRNAs. While the MFE objective (Equation 1) is a minimization over a minimization, this new objective is a minimization over a summation, which cannot be solved by DP such as LinearDesign, and is likely NP-hard.

## 4 Continuous relaxation via probabilistic lattice

Given the difficulty of optimizing our new objective, a common solution is continuous relaxation, which generalizes the discrete design space and objective into their continuous counterparts. First, we introduce a probabilistic distribution, D:X(p)↦[0,1], over the design space X(p), assigning a probability to each valid mRNA candidate for the given target protein p. A natural next step is to generalize the concept of EFE to such distributions. For a distribution over mRNA sequences, we define the *expected* EFE, ${\tilde{\Delta G^{{}^{\circ}}}}_{\text{ens}}(D)$, as:


$${\tilde{\Delta G^{{}^{\circ}}}}_{\text{ens}}(D)\overset{\Delta }{=}E_{x∼D(\cdot )}[\Delta G_{\text{ens}}^{{}^{\circ}}(x)]=-RTE_{x∼D(\cdot )}[\text{log}\;Q(x)].$$

where ${\tilde{\Delta G^{{}^{\circ}}}}_{\text{ens}}(D)$ reduces to $\Delta G_{\text{ens}}^{{}^{\circ}}(x)$ if the distribution D degenerates to a single RNA sequence x such that D(x)=1.

These generalizations together lead to the following relaxed optimization problem:


(3) $$\begin{array}{l} \underset{D}{\min}\;{\tilde{\Delta G^{{}^{\circ}}}}_{\text{ens}}(D)\\ s.t.\;D:X(p)\mapsto [0,1];\sum_{x\in X(p)}D(x)=1 \end{array}$$

where the original discrete problem (2) is relaxed into this continuous form, and we can optimize it using gradient-based methods, if the objective can be computed efficiently.

To represent a distribution of mRNA sequences over the design space given by a mRNA DFA *D*, we introduce a probabilistic DFA (pDFA) defined by a 6-tuple $D=\langle D,\tau \rangle =\langle Q,\Sigma ,\delta ,q_{0},F,\tau \rangle$, where the initial five components are identical to those in *D*, and τ represents the probability function. This function assigns to each transition from state q∈Q using symbol a∈Σ a probability within the range [0, 1]. Specifically, for any state *q*, the probability distribution is given by:


$$\tau (q,\cdot ):N(q)\mapsto [0,1],\;s.t.\;\sum_{x\in N(q)}\tau (q,x)=1.$$

where N(q) denotes the set of nucleotides allowed from state *q*, as determined by the transition function δ, defining the valid transitions from *q* with nucleotide *a*.

In the pDFA D, the probability of an mRNA sequence x within the distribution defined by it is conceptualized as the product of probabilities across each node along the sequence’s path:


$$D(x)=\prod_{i}\tau (q_{i},x_{i}),\;\text{given}\;q_{0}=(0,0)\;\text{and}\;q_{i+1}=\delta (q_{i},x_{i}),$$

where $q_{0}=\text{state}(0,0)$ is the starting node, and $q_{i+1}=\delta (q_{i},x_{i})$ is the next node reached by following the edge corresponding to nucleotide $x_{i}$. Thus, each path through D is associated with a probability defined by τ, making D a distribution D:X(p)↦[0,1], over any valid mRNA sequences x∈X(p). See Fig. 2 for a pDFA.

## 5 Expected partition function and probabilistic lattice parsing

However, directly computing ${\tilde{\Delta G^{{}^{\circ}}}}_{\text{ens}}(D)$ is challenging due to the expectation over the logarithm of a sum (i.e. E log ∑):


$$\begin{array}{l} {\tilde{\Delta G^{{}^{\circ}}}}_{\text{ens}}(D)=-RTE_{x∼D(\cdot )}[\text{log}\;Q(x)]\\ =-RT\sum_{x\in X(p)}D(x)\left[\text{log}\;\left(\sum_{y\in Y(x)}e^{-\Delta G^{{}^{\circ}}(x,y)/RT}\right)\right]. \end{array}$$

### 5.1 Approximation by Jensen’s inequality

We can apply Jensen’s inequality to swap the order of E and log , resulting in the logarithm of an expectation over a sum (log E∑), which yields a surrogate for ${\tilde{\Delta G^{{}^{\circ}}}}_{\text{ens}}(D)$:


$$\begin{array}{l} {\tilde{\Delta G^{{}^{\circ}}}}_{\text{ens}}(D)=-RTE_{x∼D(\cdot )}[\text{log}\;Q(x)]\\ \geq -RT\;\text{log}\;E_{x∼D(\cdot )}[Q(x)]\overset{\Delta }{=}{\bar{\Delta G^{{}^{\circ}}}}_{\text{ens}}(D) \end{array}$$

We further show that 1) ${\tilde{\Delta G^{{}^{\circ}}}}_{\text{ens}}(D)$ and ${\bar{\Delta G^{{}^{\circ}}}}_{\text{ens}}(D)$ coincide for every integral solution; 2) and they both achieve the same local optimums at integral solutions; 3) the surrogate is usually tight. See Appendix for details.

The surrogate leads us to define *expected partition function*, $\tilde{Q}(D)$, a generalization of partition function to distribution of sequences:


$$\tilde{Q}(D)\overset{\Delta }{=}E_{x∼D(\cdot )}[Q(x)]=\sum_{x\in X(p)}D(x)(\sum_{y\in Y(x)}e^{-\Delta G^{{}^{\circ}}(x,y)/RT}),$$

which represents the expected value of the partition function across all possible RNA sequences in distribution D.

Finally, by leveraging the relation ${\bar{\Delta G^{{}^{\circ}}}}_{\text{ens}}(D)=-RT\;\text{log}\;\tilde{Q}(D)$, minimizing ${\bar{\Delta G^{{}^{\circ}}}}_{\text{ens}}(D)$ is equivalent to maximizing $\tilde{Q}(D)$, which leads to the following optimization problem:


(4) $$\begin{array}{l} \underset{D}{\max}\;\tilde{Q}(D)\\ s.t.\;D:X(p)\mapsto [0,1];\sum_{x\in X(p)}D(x)=1 \end{array}$$

See Table 1 for a summary of various objective functions.

**Table 1.** Summary of optimization objectives.

Objective	Comment	Equation
$\underset{x\in X(p)}{\min}\text{MFE}(x)=\underset{x\in X(p)}{\min}\underset{y\in Y(x)}{\min}\Delta G^{{}^{\circ}}(x,y)$	Min. MFE	(1)
$\underset{x\in X(p)}{\min}\Delta G_{\text{ens}}^{{}^{\circ}}(x)=\underset{x\in X(p)}{\min}-RT\;\text{log}\;Q(x)$	Min. EFE	(2)
$\underset{D}{\min}{\tilde{\Delta G^{{}^{\circ}}}}_{\text{ens}}(D)=\underset{D}{\min}E_{x∼D(\cdot )}[\Delta G_{\text{ens}}^{{}^{\circ}}(x)]$	Continuous	(3)
$\underset{D}{\max}\tilde{Q}(D)=\underset{D}{\max}E_{x∼D(\cdot )}[Q(x)]$	Approximation	(4)

### 5.2 Probabilistic lattice parsing for computing expected partition function

**Figure 3.**
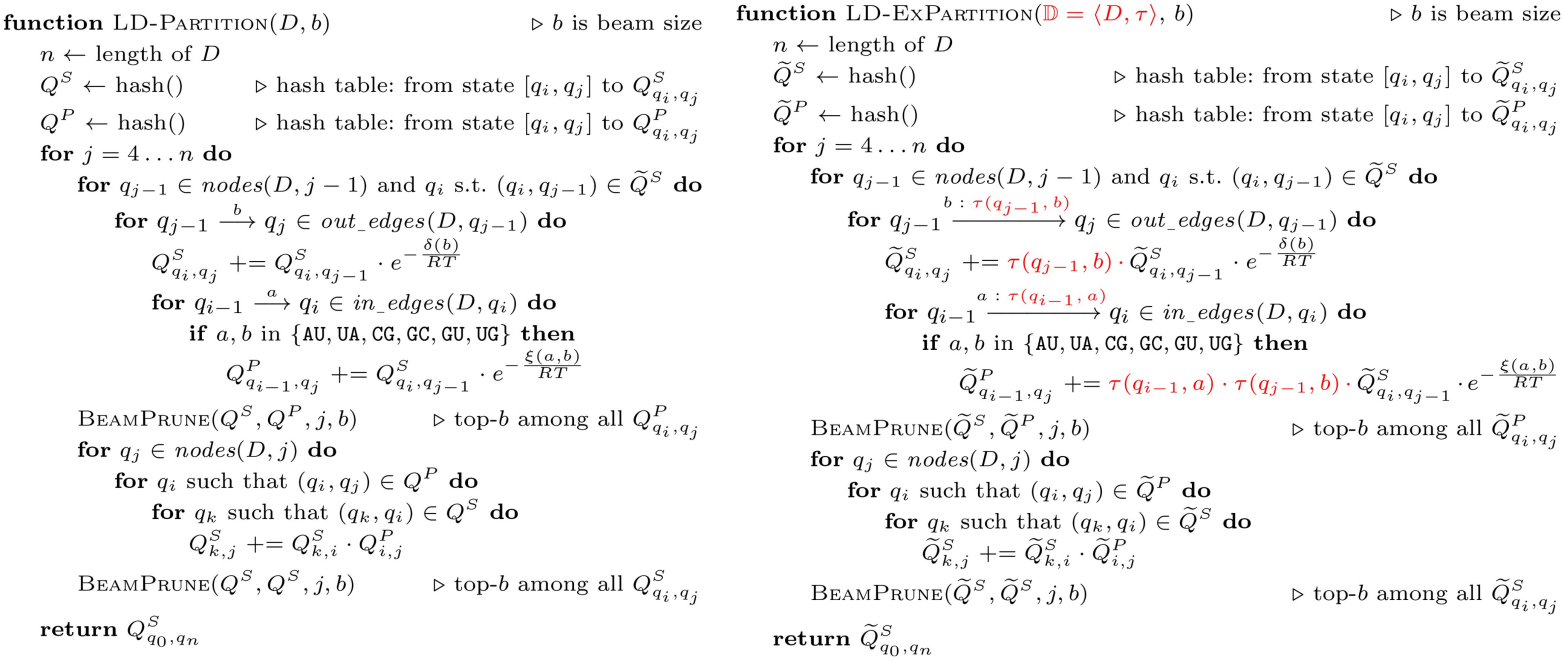
The pseudocode of partition function (left) and expected partition function (right) calculations, adapted from the lattice parsing algorithm in LinearDesign. The left one is the summation version of lattice parsing, while the right one (expected partition function) extends the left to a probabilistic lattice (Fig. 2). We use the Nussinov energy model here for simplicity of presentation, while our C++ code uses the Turner energy model.

The final optimization formulation (4), being a summation over a summation, is a practical alternative to the original problem (2) and can be efficiently solved using DP, similar to LinearDesign’s minimization over minimization. The key extensions from LinearDesign are two-fold:

- change of the DP semiring from Viterbi (min/max) to inside (sum) (Huang 2008), which results in the partition function version of LinearDesign (Fig. 3, left);
- extend lattice parsing to probabilistic lattice parsing, which computes the expected partition function $\tilde{Q}(D)$ (Fig. 3, right).

For simplicity of presentation, we use the Nussinov-Jacobson energy model for the pseudocode, but our C++ code uses the Turner energy model (Turner and Mathews 2010), and we need to make the following non-trivial changes to the pseudocode. First, we employ different nonterminals as in LinearFold (Huang *et al.* 2019) and LinearPartition (e.g. hairpin candidates $H_{i,j}$, pairs $P_{i,j}$, and multiloop candidates $M_{i,j}^{1}$, $M_{i,j}^{2}$, $M_{i,j}$). But more importantly, we also extend $P_{i,j}$ to $P_{i,j,t}$ where t∈{AU,UA,CG,GC,GU,UG} is the pair type of (i,j), following the implementation of lattice parsing in LinearDesign (Zhang *et al.* 2023). This is because, for example, when we extend $P_{i,j}$ to $P_{i-1,j+1}$ form a stack, we need to know the identity of the (i,j) pair and the (i−1,j+1) pair, which are no longer deterministic given a distribution of sequences. We also need this information for terminal mismatches.

## 6 Constrained optimization: projected gradient descent

In the final optimization problem (4), the goal is to maximize the objective function $\tilde{Q}(D)$ over the valid probability distributions D for mRNA sequences in the design space, which is formulated as a constrained optimization problem. We adopt Projected Gradient Descent to find a robust solution to the constrained optimization problem.

In each iteration, we first compute the gradient of our objective function, $\tilde{Q}(D_{\theta })$, with respect to the parameters θ. We define the parameters of the probability distribution D by expressing it as the product of individual probabilities, thus:


$$D_{\theta }(x)=\prod_{i}\theta_{q_{i},x_{i}}=\prod_{i}\tau (q_{i},x_{i})=D(x),$$

where $\theta_{q_{i},x_{i}}=\tau (q_{i},x_{i})$ stands for the probability of selecting the nucleotide $x_{i}$ at the node $q_{i}$ within a lattice, and θ encompasses all such probabilities for each nucleotide at each node.

Upon computing the gradient, we update our parameters by stepping in the positive gradient direction:


$$\theta^{\prime }=\theta^{\left(t\right)}+\alpha \nabla \tilde{Q}(D_{\theta^{\left(t\right)}}),$$

where α is a learning rate, and *t* denotes the current iteration.

Next, we need to project $\theta^{\prime }$ back onto the set of valid multinomial distributions. This can be done by projecting the parameters $\theta_{q}$ in each node *q* back to the probability simplex, which is equivalent to solving to following problem:


$$\begin{array}{l} \theta_{q}^{\left(t+1\right)}={\text{argmin}}_{\theta_{q}}{\left\|\theta_{q}-\theta_{q}^{\prime }\right\|}_{2}^{2}\\ \text{subject}\;\text{to}\;\sum_{x\in N(q)}\theta_{q,x}=1,\;\theta_{q,x}\geq 0,\forall x\in N(q) \end{array}$$

The equation above represents the projection of $\theta_{q}^{\left(t+1\right)}$ onto the probability simplex, where ${\left\|\cdot \right\|}_{2}^{2}$ denotes the Euclidean norm. The constraints ensure that $\theta_{q}$ is a valid probability distribution over N(q). The solution to this problem can be efficiently computed using algorithms for simplex projections.

This process of gradient update and projection is repeated until the changes in θ or the value of the objective function $\tilde{Q}(D_{\theta })$ becomes sufficiently small, which indicates that the solution has converged. Algorithm 1 presents a summary of the method.

Algorithm 1. Projected Gradient Descent

 Initialize $\theta^{\left(0\right)}$ such that each $\theta_{q_{i},x_{i}}^{\left(0\right)}$ is a valid distribution

 **while** not converged **do**

  Compute gradient $\nabla \tilde{Q}(D_{\theta^{\left(t\right)}})$

  Update $\theta^{\left(t+1\right)}=\theta^{\left(t\right)}+\alpha \nabla \tilde{Q}(D_{\theta^{\left(t\right)}})$

  Project $\theta_{q}^{\left(t+1\right)}$ onto probability simplex for each node *q*

  Update t=t+1

 **return** Solution $\theta^{*}$ to the optimization problem

## 7 Related work

**Figure 4.**
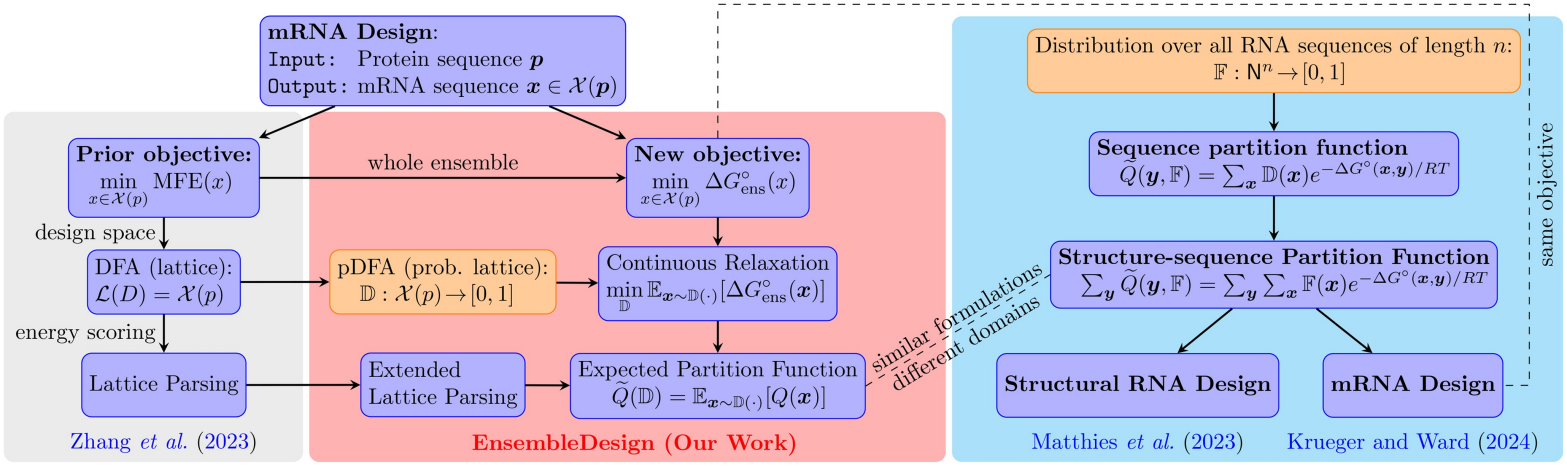
Comparison between our work with Zhang *et al.* (2023), Matthies *et al.* (2024), and Krueger and Ward (2024).

The recent work by Matthies *et al.* (2024) proposes a concept called “structure-sequence partition function” which is very similar to our expected partition function, but from a different motivation and for the different task of (non-coding) RNA design for a given secondary structure (see Fig. 4 for a comparison).

**Figure 5.**
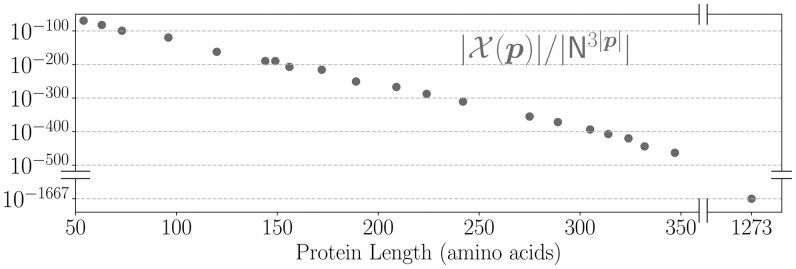
The set of valid mRNA sequences X(p) for a given protein is a tiny fraction among all possible RNA sequences, and the valid ratio decreases exponentially with sequence length. Each dot depicts a protein in Table 2 (UniProt samples and the spike protein).

After the initial release of our preprint on arXiv, a related parallel work by Krueger and Ward (2024) extended the above framework of Matthies *et al.* (2024) for mRNA design optimizing EFE, the same problem we studied. The key difference is that their sequence distribution is a simple product of independent distributions over N={A,C,G,U} at each position. As a result, their domain $N^{3|p|}$ contains *all* RNA sequences of length 3|p|, but most of them are invalid and do not encode the given protein due to the lack of any constraint in the representation (see Fig. 5 for the validity ratio). By contrast, our algorithm extends the design space representation from LinearDesign (Zhang *et al.* 2023), which makes sure the domain of our distribution D is exactly the set X(p) of *all and only* the mRNA sequences encoding protein p. To ensure the validity of sequences sampled from their distribution, they had to introduce an additional loss term that evaluates the probability of a sampled sequence encoding the target protein to rule out invalid mRNAs. Our algorithm runs on CPUs and is also much more scalable than theirs which runs on GPUs. Supplementary Figure S7 compares the running times of EnsembleDesign and Krueger and Ward (2024). Their method is orders of magnitude slower than EnsembleDesign and has higher asymptotic complexity. In particular, for “eGFP+degron” protein (284 aa), their method takes 27.0 hours while ours only takes 28.5 minutes (57× speedup). Even when utilizing advanced GPU hardware (NVIDIA H100, with 80GB of VRAM), their method can only design proteins up to 382aa, significantly limiting its practicality for real-world applications.

## 8 Evaluation results

In this section, we evaluate EnsembleDesign through a series of experiments designed to assess its effectiveness and characteristics. We conducted a random sampling of 20 natural protein sequences from the UniProt database (The UniProt Consortium 2022). These sequences exhibit a diverse range of lengths, varying from 50 to 350 amino acids, which allows for an analysis across different protein sizes. We also use the SARS-CoV-2 spike protein as a representative sample for long protein sequences. The specific sequence IDs, along with their corresponding length statistics, are detailed in Table 2.

**Table 2.** Comparative results of baseline and EnsembleDesign across protein sequences. The best result for each protein (row) is highlighted in bold.^a^

UniProt proteins												
	Length	LinearDesign		Random Walk (100 steps)			EnsembleDesign (ours)					
		MFE solution					Soft-MFE ε=0.5, *b* = 100			Soft-MFE ε=0.5, *b* = 200		
ID	Protein/mRNA	$\Delta G^{{}^{\circ}}$	$\Delta G_{\text{ens}}^{{}^{\circ}}$	$\Delta \Delta G^{{}^{\circ}}$	$\Delta \Delta G_{\text{ens}}^{{}^{\circ}}$	$\Delta_{\text{codon}}^{\%}$	$\Delta \Delta G^{{}^{\circ}}$	$\Delta \Delta G_{\text{ens}}^{{}^{\circ}}$	$\Delta_{\text{codon}}^{\%}$	$\Delta \Delta G^{{}^{\circ}}$	$\Delta \Delta G_{\text{ens}}^{{}^{\circ}}$	$\Delta_{\text{codon}}^{\%}$
Q13794	54 aa/162 nt	−112.20	−113.39	0.20	−**0.19**	3.7	0.20	−**0.19**	3.7	0.20	−**0.19**	3.7
Q9UI25	63 aa/189 nt	−124.40	−126.07	0.00	−**0.16**	7.9	0.00	−**0.16**	7.9	0.00	−**0.16**	7.9
Q9BZL1	73 aa/219 nt	−113.00	−114.87	0.00	−0.25	6.8	0.00	−**0.38**	5.5	0.00	−**0.38**	6.8
P60468	96 aa/288 nt	−232.60	−234.36	0.00	−0.14	2.1	0.20	−**0.38**	4.2	0.20	−**0.38**	4.2
Q9NWD9	120 aa/360 nt	−223.90	−226.36	0.00	0.00	0.8	0.10	−**0.67**	4.2	0.10	−**0.67**	4.2
P14555	144 aa/432 nt	−273.60	−275.06	0.00	0.00	0.0	0.20	−**0.41**	3.5	0.20	−**0.41**	3.5
Q8N111	149 aa/447 nt	−334.00	−335.89	0.80	−0.05	0.7	0.80	−**0.51**	5.4	0.80	−**0.51**	4.7
P63125	156 aa/468 nt	−296.20	−299.18	0.00	−**0.42**	3.2	0.50	−0.41	5.1	0.50	−0.41	5.1
Q6XD76	172 aa/516 nt	−424.40	−427.19	0.00	−0.31	1.2	0.00	−**0.63**	2.9	0.00	−**0.63**	1.7
P0DMU9	189 aa/567 nt	−359.40	−361.48	0.00	−0.62	2.6	0.00	−**0.64**	3.7	0.00	−**0.64**	3.2
P0DPF6	209 aa/627 nt	−542.90	−545.55	0.00	−0.42	1.0	0.00	−**0.52**	1.9	0.00	−**0.52**	1.9
Q9HD15	224 aa/672 nt	−530.00	−532.98	0.00	−0.21	2.2	10.30	10.14	18.8	0.00	−**0.32**	3.6
Q6T310	242 aa/726 nt	−500.90	−504.29	0.00	−0.24	1.2	0.80	−**0.53**	3.7	0.80	−**0.53**	3.3
Q9BRP0	275 aa/825 nt	−583.70	−586.50	0.00	−0.34	0.7	4.60	2.11	29.5	0.50	−**0.99**	3.6
P56178	289 aa/867 nt	−602.80	−606.58	0.00	−0.46	0.3	0.00	−**0.92**	2.8	0.00	−**0.92**	2.8
Q8NH87	305 aa/915 nt	−564.10	−572.37	0.20	−0.46	1.0	0.20	−**1.06**	3.6	0.20	−**1.06**	3.9
Q8NGU1	314 aa/942 nt	−606.30	−612.55	0.00	−0.12	1.3	4.40	2.19	8.6	0.80	−**0.87**	5.1
Q8NGC9	324 aa/972 nt	−576.40	−582.39	0.00	−0.47	1.9	5.30	3.88	23.5	0.20	−**1.04**	4.3
Q99729	332 aa/996 nt	−663.00	−667.21	0.00	−0.53	0.6	0.00	−**1.00**	3.0	0.00	−**1.00**	3.0
Q9P2M1	347 aa/1041 nt	−656.10	−663.54	0.00	−0.61	0.9	1.10	−1.41	5.5	1.00	−**1.53**	5.5

SARS-CoV-2 spike protein									
		LinearDesign		Random Walk			EnsembleDesign, soft-MFE ε=0.5, *b* = 300		
SPIKE	1273 aa/3819 nt	−2486.70	−2511.49	0.00	−0.82	0.2	0.50	−**4.35**	3.8

a The first section of this table presents the best outcomes from 20 independent runs per protein sequence, comparing the random walk baseline and our proposed method on UniProt samples. The second section details results for the SARS-CoV-2 spike protein, based on 40 independent runs. For each protein, we report key evaluation metrics: the minimum free energy ($\Delta G^{{}^{\circ}}$) and the ensemble free energy ($\Delta G_{\text{ens}}^{{}^{\circ}}$). The table presents both the *change* in $\Delta G^{{}^{\circ}}$ (as $\Delta \Delta G^{{}^{\circ}}$) and the *change* in $\Delta G_{\text{ens}}^{{}^{\circ}}$ (as $\Delta \Delta G_{\text{ens}}^{{}^{\circ}}$), each computed relative to the MFE solution from LinearDesign. We also report the *percentage of codon changes* for each design ($\Delta_{\text{codon}}^{\%}$) (with respect to LinearDesign). EnsembleDesign consistently achieves larger improvements in EFE compared to the random walk baseline at the expense of slightly reduced MFE stability.

### 8.1 Implementation details

We adapted and modified the mRNA design lattice parsing framework originally developed by Zhang *et al.* (2023). Our extensions primarily focus on the computation of the expected partition function, adopting a methodology akin to that used in LinearPartition (Zhang *et al.* 2020). Additionally, we also incorporated the beam pruning heuristic to accelerate this computation. Regarding the calculation of gradients, we leveraged the properties of the inside-outside algorithm, which allows us to derive the gradients directly by combining the results from both the inside and outside phases of the algorithm; see Eisner (2016).

Throughout the development and experimental phases, we observed that the projected gradient method, which primarily considers local statistics of the optimization problem, is significantly influenced by the initial distribution, denoted as $\theta^{\left(0\right)}$, in Algorithm 1. The choice of $\theta^{\left(0\right)}$ has a substantial impact on the final solution attained by our program. To address this, we implemented the following strategies:

- **Initializing close to MFE solution:** Rather than starting from a random distribution $D_{\text{rand}}$, which often leads to suboptimal solutions especially for longer proteins, we propose using the MFE solution $D_{\text{MFE}}$ (a distribution contains only the MFE mRNA sequence) from LinearDesign (Zhang *et al.* 2023) as the initial point. However, directly initializing the initial distribution $D_{\theta^{\left(0\right)}}$ with the MFE solution may lead to the algorithm becoming trapped in the MFE solution. To mitigate this, we introduce a *soft-MFE* initialization strategy:
  $$D_{\text{soft-MFE}}(\varepsilon )=\varepsilon D_{\text{rand}}+(1-\varepsilon )D_{\text{MFE}},$$
  blending the random distribution $D_{\text{rand}}$ with the MFE solution $D_{\text{MFE}}$, controlled by the parameter ε∈[0,1]. Setting ε to 0 reduces $D_{\text{soft-MFE}}$ to MFE initialization $D_{\text{MFE}}$, while setting ε to 1 transforms it into random initialization $D_{\text{rand}}$.
- **Parallelization with multiple runs:** Another strategy is to experiment with various initializations of $\theta^{\left(0\right)}$, executing the gradient descent process in parallel. The best solution can then be selected from the outcomes of these concurrent runs.

### 8.2 Baseline method

For comparative purposes, we implemented a *random walk* baseline. In this approach, we continuously refine the best mRNA sequence we have identified by randomly altering one codon at a time. Initially, the best mRNA sequence is set to the MFE solution from LinearDesign. At each step, we randomly select an amino acid and substitute its corresponding mRNA segment with a random codon. The objective value of the new mRNA sequence is then evaluated; if it shows an improvement over the current best sequence, it is recorded as the new optimal sequence. This process is repeated until a predetermined number of steps is reached.

### 8.3 Main results

In our principal experiments, we conducted 20 independent runs for each protein sequence using both the baseline method and EnsembleDesign. Each run was initialized with a distinct random seed, ensuring consistency across different methods and settings. The final reported result, $\Delta G_{\text{ens}}^{{}^{\circ}}$, for each method and setting was determined by selecting the best mRNA sequence evaluated by LinearPartition (using the -V -p -b0 -d0 options, i.e. no dangles, and exact search) from all the runs. For the baseline method, we implemented a 100-step random walk for each run. By contrast, EnsembleDesign employed a 30-step projected gradient descent, as we consistently observed model convergence within this number of iterations for proteins of varying lengths. For the initialization parameter ε in our method, we consistently used ε=0.5, as empirical testing revealed this to be the most effective setting across different proteins.

Table 2 presents the comparative results of these different methods. At a glance, the random walk method provides a robust baseline, generally yielding mRNA sequences with lower EFE ($\Delta G_{\text{ens}}^{{}^{\circ}}$) across the tested protein sequences. However, an analysis of the percentage change in codons compared to the MFE solution reveals that the random walk baseline tends to explore sequences in close proximity to the MFE solution (typically within about 1–2% change for longer proteins), indicating its potential limitations for longer proteins.

**Figure 6.**
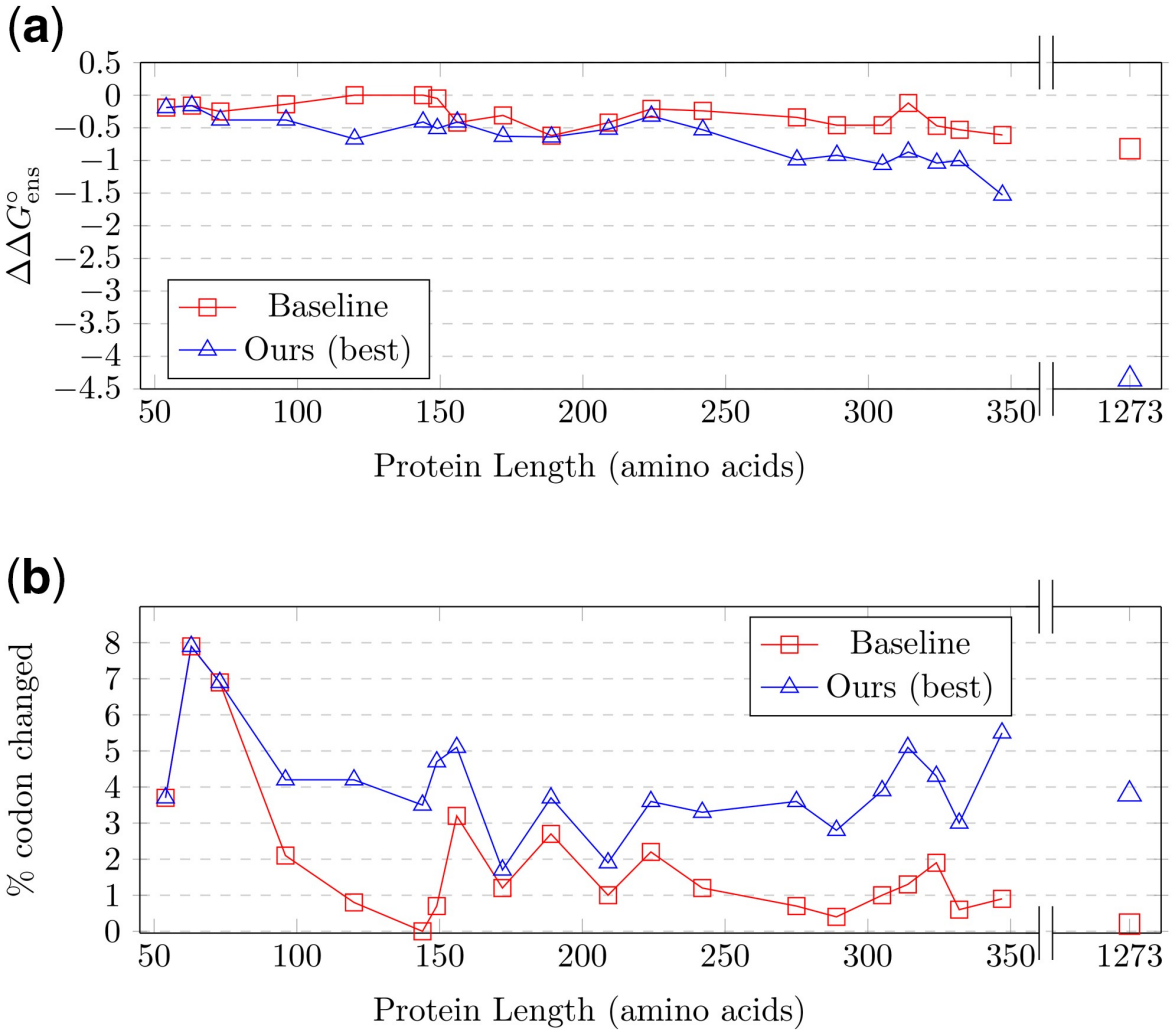
Comparative analysis of ensemble free energy variations in subfigure (a) and codon changes in subfigure (b). These subfigures compare the baseline and EnsembleDesign, with *b* = 200 for UniProt proteins and *b* = 300 for the spike protein, focusing on codon variability and $\Delta \Delta G_{\text{ens}}^{{}^{\circ}}$ across a range of protein lengths, according to the results in Table 2.

In contrast to the baseline, EnsembleDesign demonstrated the ability to identify more diverse mRNA solutions, differing more significantly from the MFE solution than those found by the baseline. This is particularly evident in the case of longer proteins, where the best solutions from our method display a 3–5% variation in codons relative to the MFE solutions, as depicted in Fig. 6b. Additionally, EnsembleDesign not only explores a broader range of mRNA sequences than the baseline but also consistently achieves lower EFE for the best-found sequences. Moreover, in scenarios with a beam size of 200, there is a discernible trend: for longer sequences, our method tends to yield solutions with greater improvements compared to the baseline method, as illustrated in Fig. 6a. These findings indicate the potential advantages of our method, especially for sequences of extended length.

We further test EnsembleDesign on the SARS-CoV-2 Spike Protein, comprising 1273 amino acids, and compare it with both the Random Walk baseline and LinearDesign. As indicated in Table 2, our method’s best solution exhibits a 3.8% codon alteration, significantly surpassing the baseline’s 0.2% variation from the MFE solution. This underscores EnsembleDesign’s advantage for long sequences. Additionally, our method markedly improves the EFE, as depicted in Supplementary Fig. S6. While aiming for superior EFE, our solution accepts a trade-off in MFE, unlike the baseline, which maintains the MFE but with lesser EFE improvement.

To test whether EnsembleDesign’s solutions could be further refined, we initialized the Random Walk procedure from our best designs and ran it for 300 steps under the same settings. As shown in Supplementary Table S3, the resulting sequences showed minimal improvement, suggesting that EnsembleDesign finds strong local optima. In contrast, when starting from the MFE solution from LinearDesign (Supplementary Table S2), it achieves notable gains—yet the final designs still underperform compared to EnsembleDesign.

### 8.4 Byproducts: lower unpaired probabilities and flatter distributions

**Figure 7.**
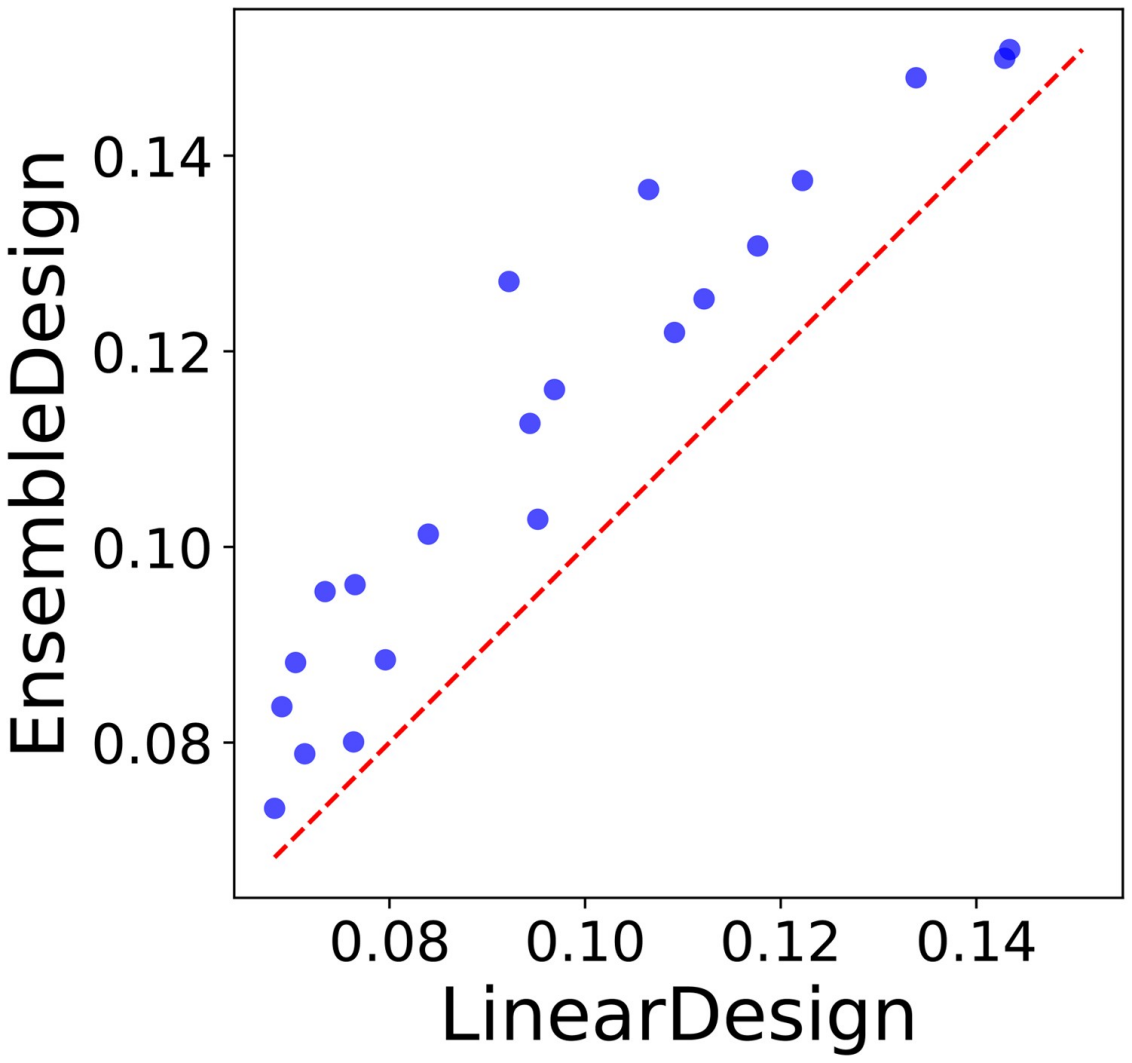
Comparison of the average *positional structural entropy* (Garcia-Martin and Clote 2015) for mRNA sequences designed by EnsembleDesign vs. LinearDesign. The positional structural entropy $H_{2}(i)$ at position *i* is defined as $H_{2}(i)=-\sum_{j=1}^{n}p_{i,j}\ln p_{i,j}$, where $p_{i,j}$ represents either the base-pairing probability (i≠j) or the unpaired probability (i=j).

In this section, we present the byproducts of our approach, specifically the reduction in unpaired probabilities and flatter distributions of Boltzmann ensembles. Table 3 presents the AUP for LinearDesign, Random Walk, and EnsembleDesign across 21 proteins. Notably, our approach achieves the lowest AUP, reducing it by 0.0024 compared to LinearDesign. This reduction is crucial as lower AUP correlates with enhanced mRNA stability and reduced degradation risks (Leppek *et al.* 2022). Figure 7 illustrates the average positional entropy comparison between mRNA sequences designed using LinearDesign and EnsembleDesign. The results clearly show that our method produces sequences with significantly higher entropy, reflecting a more uniform base-pairing probability distribution.

**Table 3.** Comparison of the mean AUP over all 21 proteins in Table 2 for LinearDesign, Random Walk, and our work.

	LinearDesign	Random Walk	EnsembleDesign (ours)
AUP	0.1973	0.1966 (−0.0007)	0.1949 (−0.0024)

The values in parentheses show the difference from LinearDesign. A lower AUP is preferred for mRNA vaccine, as AUP correlates with degradation (Leppek *et al.* 2022). EnsembleDesign consistently lowers AUP for the 21 proteins and achieves the lowest AUP, reducing it by 0.0024 from LinearDesign.

**Figure 8.**
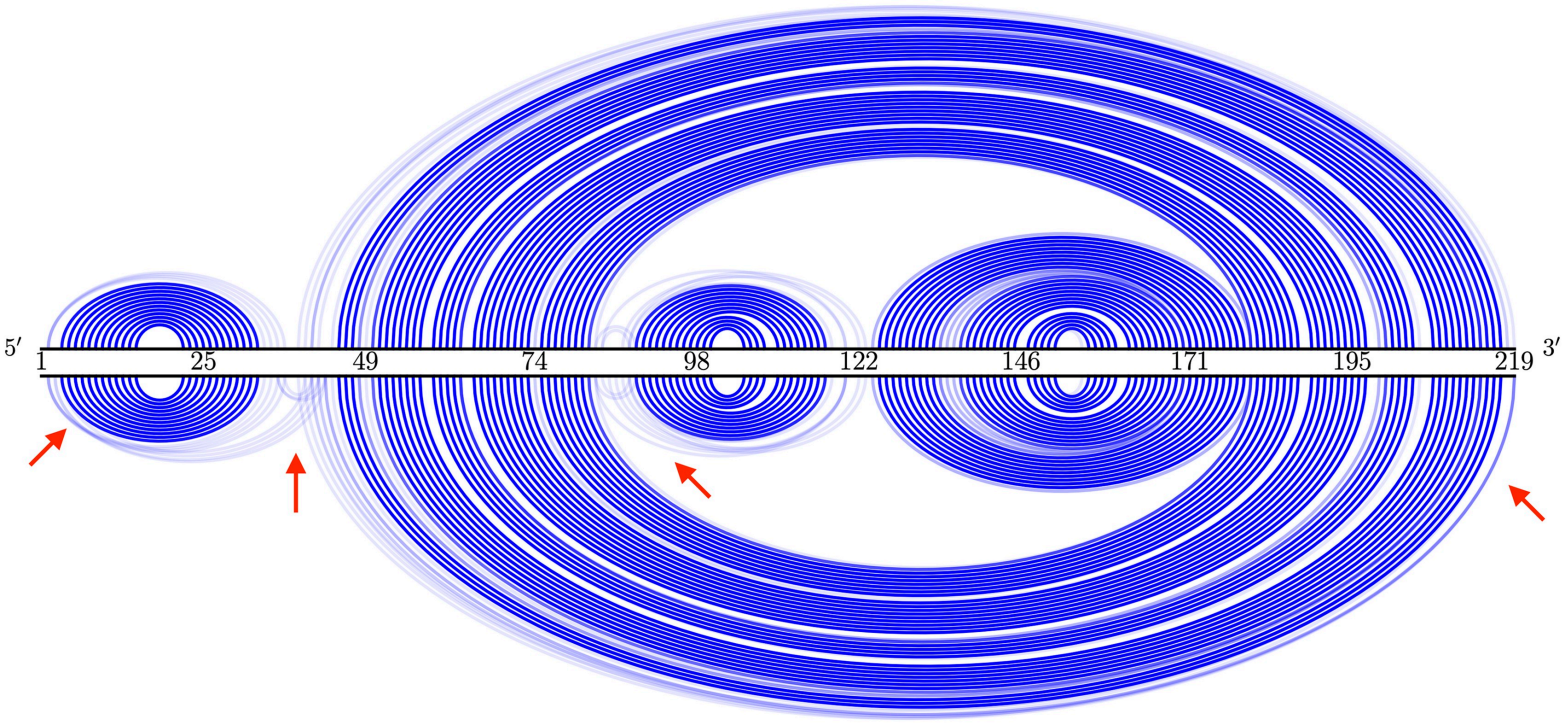
Base-pairing probabilities comparison between LinearDesign (top) and our (bottom) solutions for protein Q9BZL1. Our base pairs have higher probabilities (see arrows), suggesting more conformational flexibility.

**Figure 9.**
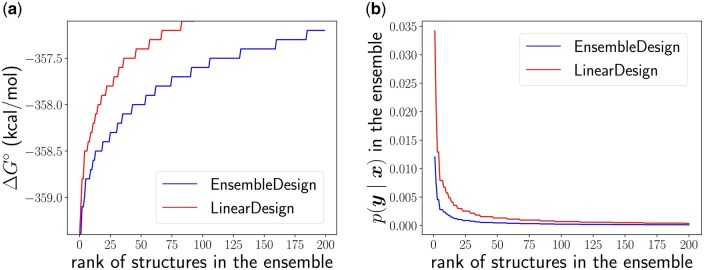
Energy landscape (a) and Boltzmann probabilities (b) of near-optimal structures for protein P0DMU9. Compared to LinearDesign, our method produces a flatter energy landscape and a flatter Boltzmann distribution, indicating reduced concentration around the MFE and near-MFE structures.

As a case study, Fig. 8 compares the base-pairing probabilities for protein Q9BZL1. Our solutions exhibit higher probabilities in key regions (highlighted by the arrows), indicating increased conformational flexibility, which is beneficial for translation. Figure 9 displays the energy landscapes (a) and Boltzmann distributions (b) for near-optimal structures of protein P0DMU9. EnsembleDesign results in a noticeably flatter energy landscape and a more evenly distributed probability across suboptimal structures compared to LinearDesign. This suggests enhanced structural diversity and flexibility, which align with our goal of reducing ribosome stalling and promoting efficient translation.

### 8.5 Beam pruning search error analysis

In this part, we aimed to quantify the search error introduced by the beam pruning method. To achieve this, we tracked and recorded the objective values computed at various beam sizes throughout the 30-step optimization process. Our analysis focused on comparing these values with the ‘true’ objective value, which we obtained in the absence of beam pruning. The results, illustrated in Supplementary Fig. S4, display the relative search error across different sequence lengths and various stages of optimization.

The findings reveal that search errors tend to be more significant during the early stages of optimization when the distributions are relatively “soft”, characterized by higher entropy (see Supplementary Fig. S5b in Appendix). For instance, in the initial iterations (1–5), we observed a maximum relative error of approximately 16%, with an average error around 5%. In contrast, during the later stages of the process (iterations 11–30), the maximum relative error notably decreases to below 1.25%, and the mean error falls to <0.25%. This trend suggests that the impact of beam pruning on search accuracy diminishes as the optimization progresses and the distributions become more “focused” or “sharpened”.

In our subsequent analysis, we delved deeper into the relationship between the entropy of distributions and the search error attributable to beam pruning during the optimization process. The data points for this analysis were consistent with those used in the previous experiments. In this context, we quantify the entropy of a distribution as the average entropy over all nodes in the mRNA DFAs. The entropy of a specific node in a DFA, for a given distribution D, is mathematically computed as follows:


$$\text{Entropy}(D,q)=-\sum_{x\in N(q)}D_{q}(x){\;\text{log}\;}_{2}D_{q}(x),$$

where *q* represents a node in the mRNA DFA, and N(q) denotes the set of nucleotides that are permissible at node *q*. This set is determined by the transition function δ(q,a), which specifies the valid transitions from node *q* given nucleotide *a*.

As depicted in Supplementary Fig. S5a, there is a visible trend that, for a given beam size, the relative search error increases together with the entropy of the distribution. This observation underpins the idea that higher uncertainty (entropy) in the distribution leads to greater search errors during optimization. Furthermore, Supplementary Fig. S5b provides a clear illustration of the entropy dynamics throughout the optimization process. Notably, at the start stage, the entropy values are at their peak, gradually decreasing as the process progresses. This trend is consistent regardless of the protein length.

### 8.6 Trade-off between MFE and EFE

We take a deeper look at the results all 20 runs for four of the longest proteins examined in our UniProt set: Q8NGU1, Q8NGC9, Q99729, and Q9P2M1. We visualize the outcomes as points on a 2D plane based on their MFE and EFE values, as calculated by LinearFold and LinearPartition, respectively (Supplementary Fig. S3). Similarly, we visualize the best design found by all three methods on the SARS-CoV-2 Spike Protein in Supplementary Fig. S6.

A key observation from the results is the tendency of the baseline method to yield solutions clustered around the MFE solution. This pattern is consistent with our earlier findings (Fig. 6b), indicating the baseline’s limited exploration range, primarily near the initial MFE solution. Conversely, our method demonstrates a broader search capability, generating more diverse solutions that significantly deviate from the MFE baseline.

While our approach outperforms the baseline in terms of solution diversity, it is crucial to note that it can sometimes converge to suboptimal solutions with higher EFE than the MFE solution. This outcome contrasts with the baseline method, which consistently improves or matches the MFE solution. Our method’s divergence from the initial distribution during gradient descent may lead to local optima without prior knowledge of their EFE values. To address the risk of converging to suboptimal solutions, we emphasize the importance of multiple initializations and runs. By exploring various starting points, our method increases the likelihood of discovering superior solutions, especially for long protein sequences.

### 8.7 Performance comparison of mRNA design methods

Finally, we compare our proposed method with two prior works: LinearDesign (Zhang *et al.* 2023) and the parallel method by Krueger and Ward (2024). Supplementary Table S1 presents the results for various protein sequences used in Krueger and Ward (2024). Both EnsembleDesign and Krueger and Ward (2024) outperform LinearDesign, achieving lower EFE values for most cases. While the performance of our method is generally on par with Krueger and Ward (2024), it demonstrates notable improvements for longer sequences such as Nanoluciferase and eGFP + degron.

In addition to performance, the efficiency of the methods is a crucial factor. As shown in Supplementary Fig. S7, our method exhibits a significant advantage in running time, primarily due to the use of an mRNA DFA for the design space representation and beam pruning for lattice parsing. This demonstrates the practical benefits of our method in scenarios demanding both performance and computational efficiency.

## 9 Conclusions and future work

In this work, we introduced EnsembleDesign, a novel algorithm for mRNA design that minimizes EFE as an alternative to the traditional MFE objective. This new objective promotes smoother energy landscapes and conformational flexibility, which are desirable properties during translation. While this new problem is much harder than minimizing MFE, we employed continuous relaxation and approximation to eventually arrive at maximizing expected partition function. To compute this quantity, we extended LinearDesign’s lattice representation to a probabilistic lattice to encode a sequence distribution, and extended lattice parsing to probabilistic lattice parsing. Our results on 21 protein sequences showed that our approach can consistently improve over LinearDesign’s results in terms of EFE, with bigger improvements on longer sequences. We also demonstrated the interesting byproducts of lower unpaired probabilities (less likely to degrade) and smoother Boltzmann ensembles (conformational flexibility during translation). These results confirm optimizing EFE as a valuable alternative to minimizing MFE, and invite future wet lab studies to test the biological relevance.

## Author contributions

L.H. conceived the idea and implemented the Python prototype with Nussinov energy model. N.D. implemented the C++ version with Turner energy model by marrying the LinearDesign and LinearPartition codebases, conducted all empirical studies, and wrote the paper. T.Z. wrote projected gradient descent in Python, and contributed to the data analysis and baselines. W.Y.T. contributed to visualizations. D.H.M. provided the biological insights. All authors contributed to the writing.

## Supplementary data

Supplementary data are available at *Bioinformatics* online.

Conflict of interest: None declared.

## Funding

This work was supported in part by NSF [grants 2330737 to L.H. and D.H.M.] and [2009071 to L.H.].

## Supplementary Material

btaf245_Supplementary_Data

## Data Availability

The data underlying this article are available at https://github.com/LinearFold/EnsembleDesign.
