# Supplementary material for: EnsembleDesign: messenger RNA design minimizing ensemble free energy via probabilistic lattice parsing
Source: Bioinformatics. 2025 Jul 15;41(Suppl 1):i391–400. doi: 10.1093/bioinformatics/btaf245 (PMC12261492; doi:10.1093/bioinformatics/btaf245)
Supplement: btaf245_Supplementary_Data [file btaf245_supplementary_data.pdf]

Supporting Information

Messenger RNA Design for Ensemble Free Energy via Probabilistic Lattice Parsing

Ning Dai, Tianshuo Zhou, Wei Yu Tang, David H. Mathews, Liang Huang

function PARTITION($\boldsymbol{x}$)
  $n \leftarrow$ length of $\boldsymbol{x}$
  $Q \leftarrow$ hash() ▷ hash table: from span $[i, j]$ to $Q_{i,j}$
  $Q_{j,j-1} \leftarrow 1$ for all j in $1 \ldots n$ ▷ base cases
  for $j = 1$ to n **do**
    for all i such that $[i, j-1]$ in Q **do**
      $Q_{i,j} \mathrel{+}= Q_{i,j-1} \cdot e^{-\frac{\delta(x_j)}{RT}}$
      if $x_{i-1}x_j$ in {AU, UA, CG, GC, GU, UG} **then**
        for all k such that $[k, i-2]$ in Q **do**
          $Q_{k,j} \mathrel{+}= Q_{k,i-2} \cdot Q_{i,j-1} \cdot e^{-\frac{\xi(x_{i-1},x_j)}{RT}}$
  return Q ▷ Partition function $Q(\boldsymbol{x}) = Q_{1,n}$

function EXPARTITION($\mathbb{D} = \prod_{i=1}^{n} \mathbb{D}_i$) ▷ $\mathbb{D}$ is a distribution
  $\widetilde{Q} \leftarrow$ hash() ▷ hash table: from span $[i, j]$ to $\widetilde{Q}_{i,j}$
  $\widetilde{Q}_{j,j-1} \leftarrow 1$ for all j in $1 \ldots n$ ▷ base cases
  for $j = 1$ to n **do**
    for all i such that $[i, j-1]$ in $\widetilde{Q}$ **do**
      for all c in {A, C, G, U} **do** ▷ all nucs at position j
        $\widetilde{Q}_{i,j} \mathrel{+}= \mathbb{D}_j(c) \cdot \widetilde{Q}_{i,j-1} \cdot e^{-\frac{\delta(c)}{RT}}$
      for all a, b in {AU, UA, CG, GC, GU, UG} **do** ▷ all pairs
        for all k such that $[k, i-2]$ in $\widetilde{Q}$ **do**
          $\widetilde{Q}_{k,j} \mathrel{+}= \mathbb{D}_{i-1}(a) \cdot \mathbb{D}_j(b) \cdot \widetilde{Q}_{k,i-2} \cdot \widetilde{Q}_{i,j-1} \cdot e^{-\frac{\xi(a,b)}{RT}}$
  return $\widetilde{Q}$ ▷ Expected partition function $\widetilde{Q}(\mathbb{D}) = \widetilde{Q}_{1,n}$

Fig. S1. Pseudocode for computing the classical partition function $Q(\boldsymbol{x})$ for a single sequence $\boldsymbol{x}$ (top, from LinearPartition (Zhang *et al.*, 2020)) and our expected partition function $\widetilde{Q}(\mathbb{D})$ for a distribution $\mathbb{D}$ over sequences (bottom), using the Nussinov-Jacobson energy model for simplicity of presentation. The former can be viewed as a special case of the latter when $\mathbb{D}$ is a one-hot distribution. The changes in the latter are colored in red. For the Turner energy model, besides different nonterminals as in LinearFold (Huang *et al.*, 2019) and LinearPartition (e.g., hairpin candidates $H_{i,j}$, pairs $P_{i,j}$, etc.), we also need to extend $P_{i,j}$ to $P_{i,j,t}$ where $t \in \{\texttt{AU}, \texttt{UA}, \texttt{CG}, \texttt{GC}, \texttt{GU}, \texttt{UG}\}$ is the pair type of (i, j), following LinearDesign (Zhang *et al.*, 2023).

Appendix A: Probabilistic mRNA DFAs

In the context of mRNA design challenges, our objective is to derive an mRNA sequence, $\boldsymbol{x} = (x_1, x_2, \ldots, x_{3n})$, where x_i represents the i-th nucleotide, corresponding to a given target protein sequence, $\boldsymbol{p} = (p_1, p_2, \ldots, p_n)$ with p_i indicating the i-th amino acid residue. This mRNA sequence must be capable of being translated into the target protein. Following the framework proposed in LinearDesign (Zhang *et al.*, 2023), we can model all feasible candidate mRNA sequences through a representation known as an mRNA Deterministic Finite Automaton (DFA).

A DFA is formally defined as a 5-tuple $D = \langle Q, \Sigma, \delta, q_0, F \rangle$:

- Q denotes the set of states,
- Σ is the alphabet, for our purposes $\Sigma = \{\text{A}, \text{C}, \text{G}, \text{U}\}$,
- q_0 is the initial state, denoted as state$(0, 0)$ in our analysis,
- F represents the set of final states, which is unique in this context,
- δ is the transition function that maps a state q and a symbol $a \in \Sigma$ to a subsequent state q', symbolically, $\delta(q, a) = q'$, which is represented as a labeled edge from q to q' with label a.

To construct the mRNA DFA, we begin by creating individual DFAs for each amino acid. These are then concatenated to form a single comprehensive DFA, $D(\boldsymbol{p})$, for the entire protein sequence $\boldsymbol{p}$. This collective DFA represents all possible mRNA sequences that could translate into the protein:

$$D(\boldsymbol{p}) = D(p_0) \circ D(p_1) \circ \cdots \circ D(p_{|\boldsymbol{p}|-1}) \circ D(\text{stop})$$

where the end state of the complete mRNA DFA is state$(3|\boldsymbol{p}| + 3, 0)$, and the length of the DFA is defined as $3|\boldsymbol{p}| + 3$.

Extending this framework, we introduce a probabilistic DFA (pDFA) defined by a 6-tuple $\mathbb{D} = \langle D, \tau \rangle = \langle Q, \Sigma, \delta, q_0, F, \tau \rangle$, where the initial five components are identical to those in D, and τ represents the probability function. This function assigns to each transition from state $q \in Q$ using symbol $a \in \Sigma$ a probability within the range $[0, 1]$. Specifically, for any state q, the probability distribution is given by:

$$\tau(q, \cdot) : \mathsf{N}(q) \mapsto [0, 1], \text{ s.t. } \textstyle\sum_{x \in \mathsf{N}(q)} \tau(q, x) = 1.$$

function BEAMPRUNE(Q^S, Q^X, j, b)
  $cands \leftarrow$ hash() ▷ hash table: from q_i to score $Q^S_{q_0,q_i} + Q^X_{q_i,q_j}$
  for $q_j \in nodes(j)$ **dos**
    for $(q_i, q_j) \in Q^X$ **do**
      $cands[q_i] \leftarrow Q^S_{q_0,q_i} + Q^X_{q_i,q_j}$ ▷ $Q^S_{q_0,q_i}$ as prefix score
  $cands \leftarrow$ SELECTTOPB($cands, b$) ▷ select top-b by score
  for $(q_i, q_j) \in Q^X$ **do**
    if $q_i \notin cands$ **then**
      delete (q_i, q_j) in Q^X ▷ prune out low-scoring states

Fig. S2. The pseudocode of beam pruning.

where $\mathsf{N}(q)$ denotes the set of nucleotides allowed from state q, as determined by the transition function δ, defining the valid transitions from q with nucleotide a. Thus, each path through $\mathbb{D}$ is associated with a probability defined by τ, making $\mathbb{D}$ a distribution over possible mRNA sequences.

In the pDFA $\mathbb{D}$, the probability of an mRNA sequence $\boldsymbol{x}$ within the distribution defined by it is conceptualized as the product of probabilities across each node along the sequence's path:

$$\mathbb{D}(\boldsymbol{x}) = \prod_i \tau(q_i, x_i), \text{ given } q_0 = (0, 0) \text{ and } q_{i+1} = \delta(q_i, x_i),$$

where $q_0 = \text{state}(0, 0)$ is the starting node, and $q_{i+1} = \delta(q_i, x_i)$ is the next node reached by following the edge corresponding to nucleotide x_i.

For the sake of clarity in pseudocode representation, we define $\text{nodes}(D, j) = \{(j, 0), (j, 1)\}$ as the set of nodes at position j, $\text{in\_edges}(q) = \{\text{edge}(q', a, q) \mid \delta(q', a) = q\}$ to denote the set of incoming edges to state q, and $\text{out\_edges}(q) = \{\text{edge}(q, a, q') \mid \delta(q, a) = q'\}$ to denote the set of outgoing edges from state q.

Appendix B: Extra Tables and Figures

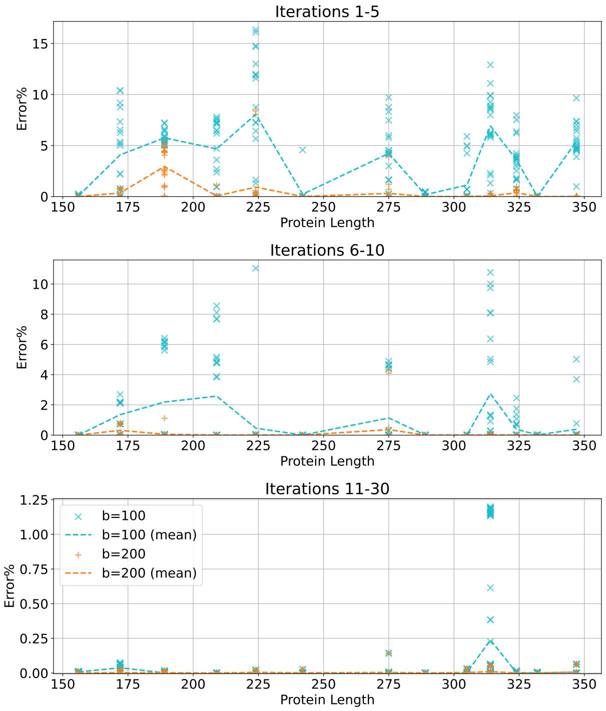

Fig. S4. Beam Pruning Search Error Relative to Protein Length. This figure illustrates the relative search error for proteins exceeding 150 amino acids, derived from 5 independent runs using varying beam sizes. Each run comprised 30 optimization steps. We categorized the iterations into three groups: 1-5, 6-10, and 11-30, to highlight the evolution of error across different optimization stages. Initially, search errors are higher, reflecting early-stage optimization challenges. Notably, the error does not escalate with increasing protein length, indicating the pruning's effectiveness and stability across varying sequence lengths.

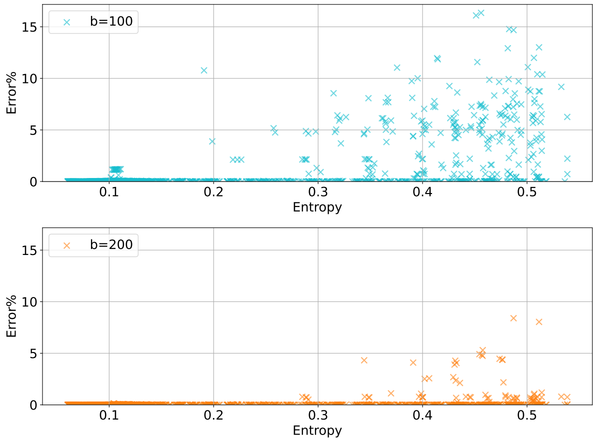

(a) Search Error% vs. Entropy

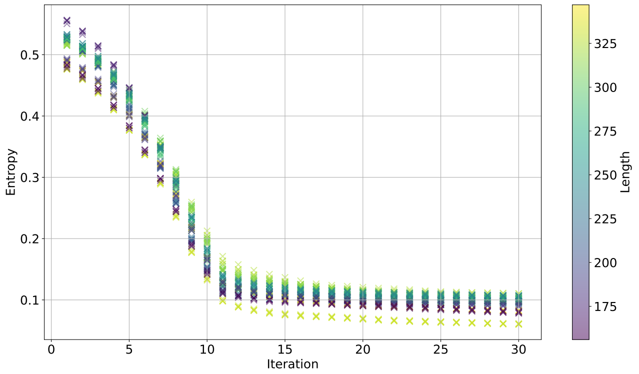

(b) Entropy vs. Iteration

Fig. S5. The interplay between Entropy and Search Error during the Optimization Process. On the left, the correlation between search error and entropy for a fixed beam size is shown (Fig. S5a). On the right, the progression of entropy values through different iterations is displayed (Fig. S5b), demonstrating a general decrease in entropy as optimization advances, irrespective of protein length.

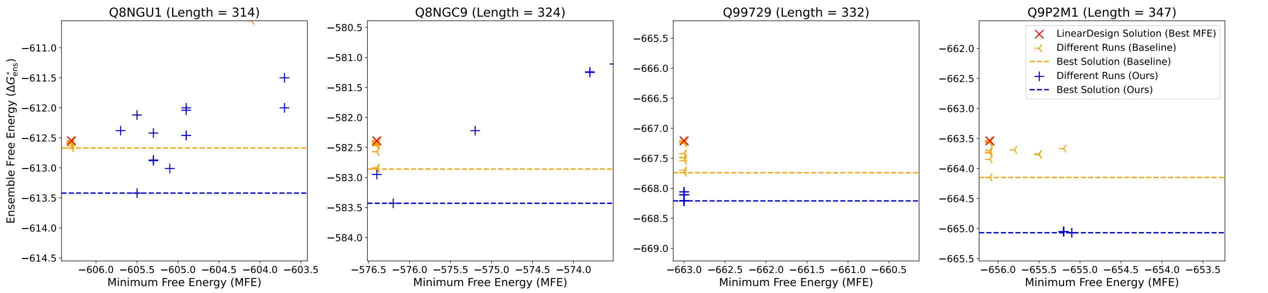

Fig. S3. Comparative 2D plots of Minimum Free Energy (MFE) and Ensemble Free Energy (EFE) values for the final solutions from 20 runs for each of the four longest protein sequences in the dataset. The focus is on the region near the MFE solution, where distinct points represent unique solutions. Some points overlap due to identical solutions found across multiple runs, while others may not be visible if they fall outside the zoomed-in area, usually indicating a higher Ensemble Free Energy compared to the MFE solution.

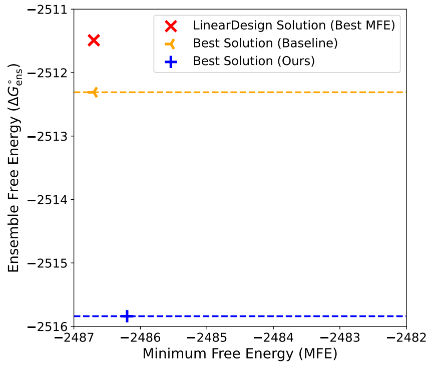

Fig. S6. Comparison of Minimum Free Energy (MFE) and Ensemble Free Energy (EFE) for the best solutions from LinearDesign, Baseline, and Our Method on the SARS-CoV-2 Spike Protein.

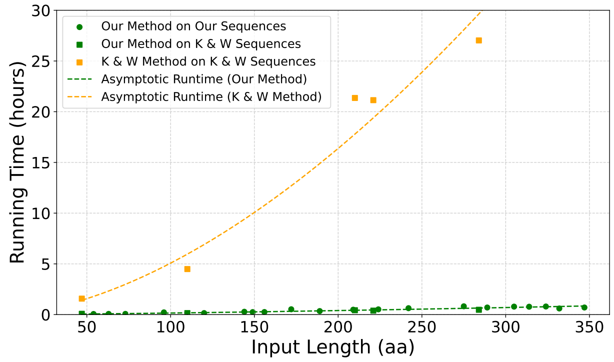

Fig. S7. Comparison of running times for Krueger and Ward (2024) (K & W) and our method across different protein sequences. Our method demonstrates a significant speed advantage due to its use of lattice parsing and beam pruning, making it highly efficient for longer protein sequences.

Table S1. Comparison of mRNA sequences designed by LinearDesign (LD), Krueger and Ward (2024) (K & W, "Reported": numbers from their paper; "Rerun": results from running their code), and EnsembleDesign (ours). Values are ΔG°_{ens} (`kcal/mol`).

	length	LD	K & W		**EnsembleDesign**
			Reported	Rerun	
MEV	47 aa	-114.84	**-114.92**	**-114.92**	**-114.92**
Mini-GFP	110 aa	-207.65	**-208.59**	-208.45	-208.53
spike RBD	210 aa	-411.55	**-412.59**	**-412.59**	-412.58
Nanoluciferase	221 aa	-452.34	-452.38	-452.38	**-452.98**
eGFP + degron	284 aa	-546.92	-547.71	**-547.96**	-547.73
average	174.4 aa	-346.66	-347.24	-347.26	**-347.35**

UniProt Proteins												
ID	Length	LinearDesign MFE solution		Random Walk (100 steps)			Random Walk (200 steps)			Random Walk (300 steps)		
	protein / mRNA	ΔG°	$\Delta G^\circ_{\text{ens}}$	$\Delta\Delta G^\circ$	$\Delta\Delta G^\circ_{\text{ens}}$	$\Delta^{\%}_{\text{codon}}$	$\Delta\Delta G^\circ$	$\Delta\Delta G^\circ_{\text{ens}}$	$\Delta^{\%}_{\text{codon}}$	$\Delta\Delta G^\circ$	$\Delta\Delta G^\circ_{\text{ens}}$	$\Delta^{\%}_{\text{codon}}$
Q13794	54 *aa* / 162 *nt*	-112.20	-113.39	0.20	**-0.19**	3.7	0.20	**-0.19**	3.7	0.20	**-0.19**	3.7
Q9UI25	63 *aa* / 189 *nt*	-124.40	-126.07	0.00	**-0.16**	7.9	0.00	**-0.16**	7.9	0.00	**-0.16**	7.9
Q9BZL1	73 *aa* / 219 *nt*	-113.00	-114.87	0.00	-0.25	6.8	0.00	-0.30	8.2	0.00	**-0.38**	9.6
P60468	96 *aa* / 288 *nt*	-232.60	-234.36	0.00	-0.14	2.1	0.00	**-0.20**	3.1	0.00	**-0.20**	3.1
Q9NWD9	120 *aa* / 360 *nt*	-223.90	-226.36	0.00	**0.00**	0.8	0.00	**0.00**	0.8	0.00	**0.00**	0.8
P14555	144 *aa* / 432 *nt*	-273.60	-275.06	0.00	0.00	0.0	0.20	**-0.41**	2.1	0.20	**-0.41**	2.8
Q8N111	149 *aa* / 447 *nt*	-334.00	-335.89	0.80	-0.05	0.7	0.00	**-0.15**	2.0	0.00	**-0.15**	2.0
P63125	156 *aa* / 468 *nt*	-296.20	-299.18	0.00	-0.42	3.2	0.00	-0.48	3.2	0.00	**-0.57**	3.8
Q6XD76	172 *aa* / 516 *nt*	-424.40	-427.19	0.00	-0.31	1.2	0.00	-0.61	2.3	0.00	**-0.63**	2.9
P0DMU9	189 *aa* / 567 *nt*	-359.40	-361.48	0.00	-0.62	2.6	0.00	**-0.64**	2.1	0.00	**-0.64**	2.1
P0DPF6	209 *aa* / 627 *nt*	-542.90	-545.55	0.00	-0.42	1.0	0.00	**-0.52**	1.4	0.00	**-0.52**	1.4
Q9HD15	224 *aa* / 672 *nt*	-530.00	-532.98	0.00	-0.21	2.2	0.00	**-0.33**	3.6	0.00	**-0.33**	4.0
Q6T310	242 *aa* / 726 *nt*	-500.90	-504.29	0.00	-0.24	1.2	0.00	-0.25	1.7	0.00	**-0.27**	2.5
Q9BRP0	275 *aa* / 825 *nt*	-583.70	-586.50	0.00	-0.34	0.7	0.00	-0.36	1.8	0.00	**-0.64**	2.5
P56178	289 *aa* / 867 *nt*	-602.80	-606.58	0.00	-0.46	0.3	0.00	-0.46	0.3	0.00	**-0.53**	1.4
Q8NH87	305 *aa* / 915 *nt*	-564.10	-572.37	0.20	-0.46	1.0	0.20	-0.67	1.6	0.20	**-0.81**	2.0
Q8NGU1	314 *aa* / 942 *nt*	-606.30	-612.55	0.00	-0.12	1.3	0.00	**-0.34**	3.2	0.00	**-0.34**	3.5
Q8NGC9	324 *aa* / 972 *nt*	-576.40	-582.39	0.00	-0.47	1.9	0.00	-0.49	1.2	0.00	**-0.56**	2.5
Q99729	332 *aa* / 996 *nt*	-663.00	-667.21	0.00	-0.53	0.6	0.00	-0.68	1.2	0.00	**-0.89**	1.5
Q9P2M1	347 *aa* / 1041 *nt*	-656.10	-663.54	0.00	-0.61	0.9	0.00	-0.85	1.7	0.00	**-1.01**	2.0
SARS-CoV-2 Spike Protein												
		LinearDesign		Random Walk (100 steps)			Random Walk (200 steps)			Random Walk (300 steps)		
SPIKE	1273 *aa* / 3819*nt*	-2486.70	-2511.49	0.00	-0.82	0.2	0.00	**-1.24**	0.4	0.00	**-1.24**	0.6

Table S2. Results of Random Walk by initializing from LinearDesign's MFE solutions with different numbers of steps. The 100-step results are the same as those in Tab. 2. For each protein, we report key evaluation metrics: the Minimum Free Energy (ΔG°) and the Ensemble Free Energy ($\Delta G^\circ_{\text{ens}}$). The table presents both the *change* in ΔG° (as $\Delta\Delta G^\circ$) and the *change* in $\Delta G^\circ_{\text{ens}}$ (as $\Delta\Delta G^\circ_{\text{ens}}$), each computed relative to the MFE solution from LinearDesign.

UniProt Proteins												
ID	Length	EnsembleDesign best solution		Random Walk (100 steps)			Random Walk (200 steps)			Random Walk (300 steps)		
	protein / mRNA	ΔG°	$\Delta G^\circ_{\text{ens}}$	$\Delta\Delta G^\circ$	$\Delta\Delta G^\circ_{\text{ens}}$	$\Delta^{\%}_{\text{codon}}$	$\Delta\Delta G^\circ$	$\Delta\Delta G^\circ_{\text{ens}}$	$\Delta^{\%}_{\text{codon}}$	$\Delta\Delta G^\circ$	$\Delta\Delta G^\circ_{\text{ens}}$	$\Delta^{\%}_{\text{codon}}$
Q13794	54 *aa* / 162 *nt*	-112.00	-113.58	0.00	**-0.06**	1.9	0.00	**-0.06**	1.9	0.00	**-0.06**	1.9
Q9UI25	63 *aa* / 189 *nt*	-124.40	-126.23	0.00	**0.00**	1.6	0.00	**0.00**	1.6	0.00	**0.00**	1.6
Q9BZL1	73 *aa* / 219 *nt*	-113.00	-115.25	0.00	**0.00**	1.4	0.00	**0.00**	1.4	0.00	**0.00**	1.4
P60468	96 *aa* / 288 *nt*	-232.40	-234.74	0.00	**-0.07**	3.1	0.00	**-0.07**	2.1	0.00	**-0.07**	2.1
Q9NWD9	120 *aa* / 360 *nt*	-223.80	-227.03	0.00	**0.00**	0.0	0.00	**0.00**	0.8	0.00	**0.00**	0.8
P14555	144 *aa* / 432 *nt*	-273.40	-275.47	0.00	**0.00**	0.7	0.00	**0.00**	0.7	0.00	**0.00**	0.7
Q8N111	149 *aa* / 447 *nt*	-333.20	-336.40	0.00	**0.00**	0.0	0.00	**0.00**	0.0	0.00	**0.00**	0.0
P63125	156 *aa* / 468 *nt*	-295.70	-299.59	-0.50	**-0.16**	1.3	-0.50	**-0.16**	1.3	-0.50	**-0.16**	1.3
Q6XD76	172 *aa* / 516 *nt*	-424.40	-427.82	0.00	**0.00**	0.0	0.00	**0.00**	0.0	0.00	**0.00**	0.0
P0DMU9	189 *aa* / 567 *nt*	-359.40	-362.12	0.00	**0.00**	0.0	0.00	**0.00**	0.0	0.00	**0.00**	0.0
P0DPF6	209 *aa* / 627 *nt*	-542.90	-546.07	0.00	**0.00**	0.5	0.00	**0.00**	1.0	0.00	**0.00**	1.4
Q9HD15	224 *aa* / 672 *nt*	-530.00	-533.30	0.00	-0.02	0.9	0.00	**-0.04**	2.2	0.00	**-0.04**	2.2
Q6T310	242 *aa* / 726 *nt*	-500.10	-504.82	0.00	**0.00**	0.0	0.00	**0.00**	0.0	0.00	**0.00**	0.0
Q9BRP0	275 *aa* / 825 *nt*	-583.20	-587.49	0.00	**-0.06**	0.4	0.00	**-0.06**	0.7	0.00	**-0.06**	0.4
P56178	289 *aa* / 867 *nt*	-602.80	-607.50	0.00	**0.00**	0.0	0.00	**0.00**	0.0	0.00	**0.00**	0.0
Q8NH87	305 *aa* / 915 *nt*	-563.90	-573.43	0.00	**-0.02**	0.3	0.00	**-0.02**	0.3	0.00	**-0.02**	0.7
Q8NGU1	314 *aa* / 942 *nt*	-605.50	-613.42	0.00	-0.05	0.6	0.00	-0.05	1.0	0.00	**-0.06**	1.3
Q8NGC9	324 *aa* / 972 *nt*	-576.20	-583.43	0.00	-0.06	0.9	-0.10	**-0.12**	2.2	-0.10	**-0.12**	2.2
Q99729	332 *aa* / 996 *nt*	-663.00	-668.21	0.00	**0.00**	0.0	0.00	**0.00**	0.0	0.00	**0.00**	0.0
Q9P2M1	347 *aa* / 1041 *nt*	-655.10	-665.07	0.00	**-0.01**	0.3	0.00	**-0.01**	0.3	0.00	**-0.01**	0.3
SARS-CoV-2 Spike Protein												
		EnsembleDesign		Random Walk (100 steps)			Random Walk (200 steps)			Random Walk (300 steps)		
SPIKE	1273 *aa* / 3819*nt*	-2486.20	-2515.84	0.20	-0.04	0.2	-0.10	-0.05	0.3	-0.10	**-0.06**	0.5

Table S3. Results of Random Walk by initializing from EnsembleDesign's best solutions with different numbers of steps. For each protein, we report key evaluation metrics: the Minimum Free Energy (ΔG°) and the Ensemble Free Energy ($\Delta G^\circ_{\text{ens}}$). The table presents both the *change* in ΔG° (as $\Delta\Delta G^\circ$) and the *change* in $\Delta G^\circ_{\text{ens}}$ (as $\Delta\Delta G^\circ_{\text{ens}}$), each computed relative to the best solution from our EnsembleDesign.

Appendix C: Experiment Protein Sequences and Results

ID	Protein Sequence
Q13794	MPGKKARKNAQPSPARAPAELEVECATQLRRFGDKLNFRQKLLNLISKLFCSGT
Q9UI25	MEEMSYGENSGTHVGSFSCSPQPSQQMKVLFVGNSFLLTPVLHRQPHLQPCNFGPEVVAPQRL
Q9BZL1	MIEVVCNDRLGKKVRVKCNTDDTIGDLKKLIAAQTGTRWNKIVLKKWYTIFKDHVSLGDYEIHDGMNLELYYQ
P60468	MPGPTPSGTNVGSSGRSPSKAVAARAAGSTVRQRKNASCGTRSAGRTTSAGTGGMWRFYTEDSPGLKVGPVPVLVMSLLFIASVFMLHIWGKYTRS
Q9NWD9	MESKEELAANNLNGENAQQENEGGEQAPTQNEEESRHLGGGEGQKPGGNIRRGRVRRLVPNFRWAIPNRHIEHNEARDDVERFVGQMMEIKRKTREQQMRHYMRFQTPEPDNHYDFCLIP
P14555	MKTLLLLAVIMIFGLLQAHGNLVNFHRMIKLTTGKEAALSYGFYGCHCGVGGRGSPKDATDRCCVTHDCCYKRLEKRGCGTKFLSYKFSNSGSRITCAKQDSCRSQLCECDKAAATCFARNKTTYNKKYQYYSNKHCRGSTPRC
Q8N111	MESRGKSASSPKPDTKVPQVTTEAKVPPAADGKAPLTKPSKKEAPAEKQQPPAAPTTAPAKKTSAKADPALLNNHSNLKPAPTVPSSPDATPEPKGPGDGAEEDEAASGGPGGRGPWSCENFNPLLVAGGVAVAAIALILGVAFLVRKK
P63125	WASQVSENRPVCKAIIQGKQFEGLVDTGADVSIIALNQWPKNWPKQKAVTGLVGVGTASEVYQSTEILHCLGPDNQESTVQPMITSIPLNLWGRDLLQQWGAEITMPAPLYSPTSQKIMTKMGYIPGKGLGKNEDGIKIPVEAKINQKREGIGYPF
Q6XD76	METRKPAERLALPYSLRTAPLGVPGTLPGLPRRDPLRVALRLDAACWEWARSGCARGWQYLPVPLDSAFEPAFLRKRNERERQRVRCVNEGYARLRDHLPRELADKRLSKVETLRAAIDYIKHLQELLERQAWGLEGAAGAVPQRRAECNSDGESKASSAPSPSSEPEEGGS
P0DMU9	MTDKTEKVAVDPETVFKRPRECDSPSYQKRQRMALLARKQGAGDSLIAGSAMSKEKKLMTGHAIPPSQLDSQIDDFTGFSKDGMMQKPGSNAPVGGNVTSNFSGDDLECRGIASSPKSQQEINADIKCQVVKEIRCLGRKYEKIFEMLEGVQGPTAVRKRFFESIIKEAARCMRRDFVKHLKKKLKRMI
P0DPF6	MFVRPESGEQGPETLAPASGAEIQRFPVPAVEPVPAPGADSPPGTALELEEAPEPSCRCPGTAQDQPSEELPDFMAPPVEPPASALELKVWLELEVAERGGQHSSSQQLPHCSQSWAQWKLWRQRPGFAIWAPLPHWRGTSLIQQSSSPAAEGPAATAAGAVCLPAGGAGEQEKEPVSRGSSRSSCSQRRPPPPGMEVCPQLGIWAICP
Q9HD15	MAELYVKPGNKERGWNDPPQFSYGLQTQAGGPRRSLLTKRVAAPQDGSPRVPASETSPGPPPMGPPPPSSKAPRSPPVGSGPASGVEPTSFPVESEAVMEDVLRPLEQALEDCRGHTRKQVCDDISRRLALLQEQWAGGKLSIPVKKRMALLVQELSSHRWDAADDIHRSLMVDHVTEVSQWMVGVKRLIAEKRSLFSEEAANEEKSAATAEKNHTIPGFQQAS
Q6T310	MRPLSMSGHFLLAPIPESSSDYLLPKDIKLAVLGAGRVGKSAMIVRFLTKRFIGDYEPNTGKLYSRLVYVEGDQLSLQIQDTPGGVQIQDSLPQVVDSLSKCVQWAEGFLLVYSITDYDSYLSIRPLYQHIRKVHPDSKAPVIIVGNKGDLLHARQVQTQDGIQLANELGSLFLEISTSENYEDVCDVFQHLCKEVSKMHGLSGERRRASIIPRPRSPNMQDLKRRFKQALSPKVKAPSALG
Q9BRP0	MPKVFLVKRRSLGVSVRSWDELPDEKRADTYIPVGLGRLLHDPPEDCRSDGGSSSGSGSSSAGEPGGAESSSSPHAPESETPEPGDAEGPDGHLATKQRPVARSKIKFTTGTCSDSVVHSCDLCGKGFRLQRMLNRHLKCHNQVKRHLCTFCGKGFNDTFDLKRHVRTHTGIRPYKCNVCNKAFTQRCSLESHLKKIHGVQQQYAYKQRRDKLYVCEDCGYTGPTQEDLYLHVNSAHPGSSFLKKTSKKLAALLQGKLTSAHQENTSLSEEEERK
P56178	MTGVFDRRVPSIRSGDFQAPFQTSAAMHHPSQESPTLPESSATDSDYYSPTGGAPHGYCSPTSASYGKALNPYQYQYHGVNGSAGSYPAKAYADYSYASSYHQYGGAYNRVPSATNQPEKEVTEPEVRMVNGKPKKVRKPRTIYSSFQLAALQRRFQKTQYLALPERAELAASLGLTQTQVKIWFQNKRSKIKKIMKNGEMPPEHSPSSSDPMACNSPQSPAVWEPQGSSRSLSHHPHAHPPTSNQSPASSYLENSASWYTSAASSINSHLPPPGSLQHPLALASGTLY
Q8NH87	MQRSNHTVTEFILLGFTTDPGMQLGLFVVFLGVYSLTVVGNSTLIVLICNDSCLHTPMYFFTGNLSFLDLWYSSVYTPKILVTCISEDKSISFAGCLCQFFFSAGLAYSECYLLAAVAYDRYVAISKPLLYAQAMSIKLCALLVAVSYCGGFINSSIITKKTFSFNFCRENIIDDFFCDLLPLVELACGEKGGYKIMMYFLLASNVICPAVLILASYLFIITSVLRISSSKGYLKAFSTCSSHLTSVTLYYGSILYIYALPRSSYSFDMDKIVSTFYTVVFPMLNLMIYSLRNKDVKEALKKLLP
Q8NGU1	MLGNYSSATEFFLLGFPGSQEVCRILFATFFLLYAVTVMGNVVIIITVCVDKCLQSPIYFFLGHLCVLEILITSTAVPFMLWGLLLPSTQIMSLTACAAQLYLYLSLGTLELALMGVMAVDRYVAVCNPLRYNIIMNSSTFIWVIIVSWVLGFLSEIWPVYATFQLTFCKSSVLDHFYCDRGQLLKVSCEDTLFREFILFLMAVFIIIGSLIPTIVSYTYIISTNLKIPSASGWRKSFSTCASHFTYVVIGYGSCLFLYVKPKQTQAAEYNRVVSLLVLVVTPFLNPFIFTLRNDKFIQAFGDGMKHCYKLLKN
Q8NGC9	MSFFFVDLRPMNRSATHIVTEFILLGFPGCWKIQIFLFSLFLVIYVLTLLGNGAIIYAVRCNPLLHTPMYFLLGNFAFLEIWYVSSTIPNMLVNILSKTKAISFSGCFLQFYFFFSLGTTECLFLAVMAYDRYLAICHPLQYPAIMTVRFCGKLVSFCWLIGFLGYPIPIFYISQLPFCGPNIIDHFLCDMDPLMALSCAPAPITECIFYTQSSLVLFFTSMYILRSYILLLTAVFQVPSAAGRRKAFSTCGSHLVVVSLFYGTVMVMYVSPTYGIPTLLQKILTLVYSVTTPLFNPLIYTLRNKDMKLALRNVLFGMRIRQNS
Q99729	MSEAGEEQPMETTGATENGHEAVPEASRGRGWTGAAAGAGGATAAPPSGNQNGAEGDQINASKNEEDAGKMFVGGLSWDTSKKDLKDYFTKFGEVVDCTIKMDPNTGRSRGFGFILFKDAASVEKVLDQKEHRLDGRVIDPKKAMAMKKDPVKKIFVGGLNPESPTEEKIREYFGEFGEIEAIELPMDPKLNKRRGFVFITFKEEEPVKKVLEKKFHTVSGSKCEIKVAQPKEVYQQQQYGSGGRGNRNRGNRGSGGGGGGGGQSQSWNQGYGNYWNQGYGYQQGYGPGYGGYDYSPYGYYGYGPGYDYSQGSTNYGKSQRRGGHQNNYKPY
Q9P2M1	MKLTSEKLPKNPFYASVSQYAAKNQKFFQWKKEKTDYTHANLVDKALQLLKERILKGDTLAYFLRGQLYFEEGWYEEALEQFEEIKEKDHQATYQLGVMYYDGLGTTLDAEKGVDYMKKILDSPCPKARHLKFAAAYNLGRAYYEGKGVKRSNEEAERLWLIAADNGNPKASVKAQSMLGLYYSTKEPKELEKAFYWHSEACGNGNLESQGALGLMYLYGQGIRQDTEAALQCLREAAERGNVYAQGNLVEYYYKMKFFTKCVAFSKRIADYDEVHDIPMIAQVTDCLPEFIGRGMAMASFYHARCLQLGLGITRDETTAKHYYSKACRLNPALADELHSLLIRQRI
SARS-CoV-2 SPIKE	MFVFLVLLPLVSSQCVNLTTRTQLPPAYTNSFTRGVYYPDKVFRSSVLHSTQDLFLPFFSNVTWFHAIHVSGTNGTKRFDNPVLPFNDGVYFASTEKSNIIRGWIFGTTLDSKTQSLLIVNNATNVVIKVCEFQFCNDPFLGVYYHKNNKSWMESEFRVYSSANNCTFEYVSQPFLMDLEGKQGNFKNLREFVFKNIDGYFKIYSKHTPINLVRDLPQGFSALEPLVDLPIGINITRFQTLLALHRSYLTPGDSSSGWTAGAAAYYVGYLQPRTFLLKYNENGTITDAVDCALDPLSETKCTLKSFTVEKGIYQTSNFRVQPTESIVRFPNITNLCPFGEVFNATRFASVYAWNRKRISNCVADYSVLYNSASFSTFKCYGVSPTKLNDLCFTNVYADSFVIRGDEVRQIAPGQTGKIADYNYKLPDDFTGCVIAWNSNNLDSKVGGNYNYLYRLFRKSNLKPFERDISTEIYQAGSTPCNGVEGFNCYFPLQSYGFQPTNGVGYQPYRVVVLSFELLHAPATVCGPKKSTNLVKNKCVNFNFNGLTGTGVLTESNKKFLPFQQFGRDIADTTDAVRDPQTLEILDITPCSFGGVSVITPGTNTSNQVAVLYQDVNCTEVPVAIHADQLTPTWRVYSTGSNVFQTRAGCLIGAEHVNNSYECDIPIGAGICASYQTQTNSPRRARSVASQSIIAYTMSLGAENSVAYSNNSIAIPTNFTISVTTEILPVSMTKTSVDCTMYICGDSTECSNLLLQYGSFCTQLNRALTGIAVEQDKNTQEVFAQVKQIYKTPPIKDFGGFNFSQILPDPSKPSKRSFIEDLLFNKVTLADAGFIKQYGDCLGDIAARDLICAQKFNGLTVLPPLLTDEMIAQYTSALLAGTITSGWTFGAGAALQIPFAMQMAYRFNGIGVTQNVLYENQKLIANQFNSAIGKIQDSLSSTASALGKLQDVVNQNAQALNTLVKQLSSNFGAISSVLNDILSRLDKVEAEVQIDRLITGRLQSLQTYVTQQLIRAAEIRASANLAATKMSECVLGQSKRVDFCGKGYHLMSFPQSAPHGVVFLHVTYVPAQEKNFTTAPAICHDGKAHFPREGVFVSNGTHWFVTQRNFYEPQIITTDNTFVSGNCDVVIGIVNNTVYDPLQPELDSFKEELDKYFKNHTSPDVDLGDISGINASVVNIQKEIDRLNEVAKNLNESLIDLQELGKYEQYIKWPWYIWLGFIAGLIAIVMVTIMLCCMTSCCSCLKGCCSCGSCCKFDEDDSEPVLKGVKLHYT

Table S4. Protein sequences used in our study: 20 from UniProt and the final one being the SARS-CoV-2 Spike Protein.

Table S5: The best mRNA designs found by LinearDesign, the Random Walk baseline, and our algorithm across all protein sequences, with the changed codons highlighted in both the baseline and our results when compared to the MFE solution from LinearDesign. We also listed the ensemble free energy (evaluated by `linearpartition -V -p -d0 -b0`) and the minimum free energy (evaluated by `linearfold -V -d0 -b0`) for each sequence. Ensemble free energy values are shown in `typewriter` font, while minimum free energy values are shown in *italic*.

ID	Method	mRNA Design
	LinearDesign<br>`-113.39 kcal/mol`<br>*-112.20 kcal/mol*	AUGCCGGGGAAGAAGGCGCGGAAGAACGCGCAGCCUUCCCCGGCACGUGCCCCAGCAGAGCUGGAGGUCGAGUGUGCAACUCAGCUCCGGAGGUUCGGCGA<br>CAAGCUGAACUUCCGGCAGAAGUUGCUCAACUUGAUCUCCAAGCUGUUCUGCUCGGGCACG
Q13794	Random Walk<br>`-113.58 kcal/mol`<br>*-112.00 kcal/mol*	AUGCCGGGGAAGAAGGCGCGGAAGAACGCGCAGCCUUCCCCGGCACGUGCCCCAGCAGAGCUGGAGGUCGAGUGUGCAACUCAGCUGCGGAGGUUCGGCGA<br>CAAGCUGAACUUCCGCCAGAAGUUGCUCAACUUGAUCUCCAAGCUGUUCUGCUCGGGCACG
	Ours<br>`-113.58 kcal/mol`<br>*-112.00 kcal/mol*	AUGCCGGGGAAGAAGGCGCGUAAGAACGCGCAGCCUUCCCCGGCACGUGCCCCAGCAGAGCUGGAGGUCGAGUGUGCAACUCAGCUGCGGAGGUUCGGCGA<br>CAAGCUGAACUUCCGGCAGAAGUUGCUCAACUUGAUCUCCAAGCUGUUCUGCUCGGGCACG
	LinearDesign<br>`-126.07 kcal/mol`<br>*-124.40 kcal/mol*	AUGGAGGAGAUGUCCUACGGAGAGAACUCUGGGACGCAUGUGGGGAGCUUCAGCUGCUCCCCACAGCCGUCCCAGCAGAUGAAGGUUCUCUUCGUAGGAAA<br>CAGUUUCCUCCUGACGCCGGUGCUGCACCGGCAGCCCCACCUCCAGCCCUGCAACUUUGGGCCGGAGGUGGUGGCGCCGCAGCGCCUG
Q9UI25	Random Walk<br>`-126.23 kcal/mol`<br>*-124.40 kcal/mol*	AUGGAGGAGAUGUCCUACGGAGAGAACUCUGGGACGCAUGUGGGGAGCUUCUCGUGCUCCCCACAGCCGUCCCAGCAGAUGAAGGUUCUCUUCGUAGGAAA<br>CAGUUUCCUCCUUACACCGGUGCUGCACCGGCAGCCCCACCUCCAGCCCUGUAAUUUUGGGCCGGAGGUGGUGGCGCCGCAGCGCCUG
	Ours<br>`-126.23 kcal/mol`<br>*-124.40 kcal/mol*	AUGGAGGAGAUGUCCUACGGAGAGAACUCUGGGACGCAUGUGGGGAGCUUCUCGUGCUCCCCACAGCCGUCCCAGCAGAUGAAGGUUCUCUUCGUAGGAAA<br>CAGUUUCCUCCUUACACCGGUGCUGCACCGGCAGCCCCACCUCCAGCCCUGUAAUUUUGGGCCGGAGGUGGUGGCGCCGCAGCGCCUG
	LinearDesign<br>`-114.87 kcal/mol`<br>*-113.00 kcal/mol*	AUGAUCGAGGUGGUCUGCAAUGACCGCCUCGGUAAGAAGGUGAGGGUGAAGUGCAACACAGAUGAUACCAUCGGGGAUCUCAAGAAGCUCAUCGCGGCCCA<br>GACGGGCACGCGAUGGAACAAGAUAGUCCUCAAGAAGUGGUACACGAUCUUCAAGGAUCAUGUAUCACUUGGGGACUAUGAGAUCCACGAUGGUAUGAAUC<br>UGGAGUUGUACUACCAG
Q9BZL1	Random Walk<br>`-115.12 kcal/mol`<br>*-113.00 kcal/mol*	AUGAUCGAGGUGGUCUGUAAUGACCGCCUCGGUAAGAAGGUUCGGGUGAAGUGCAACACAGAUGAUACCAUCGGGGAUCUCAAGAAGCUCAUCGCGGCCCA<br>GACGGGCACGCGAUGGAAUAAGAUAGUCCUCAAGAAGUGGUACACGAUCUUUAAGGAUCAUGUAUCACUUGGGGACUAUGAGAUCCACGAUGGUAUGAAUC<br>UGGAGUUGUACUACCAG
	Ours<br>`-115.25 kcal/mol`<br>*-113.00 kcal/mol*	AUGAUCGAGGUGGUCUGCAAUGACCGCCUCGGUAAGAAGGUCCGGGUAAAGUGCAACACAGAUGAUACCAUCGGGGAUCUCAAGAAGCUCAUCGCGGCCCA<br>GACGGGCACGCGAUGGAAUAAGAUAGUCCUCAAGAAGUGGUACACGAUCUUUAAGGAUCAUGUAUCACUUGGGGACUAUGAGAUCCACGAUGGUAUGAAUC<br>UGGAGUUGUACUACCAG
	LinearDesign<br>`-232.60 kcal/mol`<br>*-234.36 kcal/mol*	AUGCCCGGCCCGACGCCCAGCGGGACCAACGUUGGGUCGUCGGGCCGGUCCCCGUCGAAGGCGGUCGCGGCCCGCGCUGCGGGUUCCACAGUGAGGCAGCG<br>GAAGAAUGCCUCCUGUGGAACCCGCAGCGCGGGCCGGACCACCUCGGCGGGGACAGGAGGGAUGUGGCGAUUCUACACCGAGGACUCGCCCGGCCUGAAGG<br>UCGGGCCAGUCCCGGUGCUCGUCAUGUCCCUCCUGUUCAUUGCGAGUGUGUUCAUGCUCCAUAUUUGGGGCAAAUACACUCGCAGU
P60468	Random Walk<br>`-234.50 kcal/mol`<br>*-232.60 kcal/mol*	AUGCCCGGCCCGACGCCCAGCGGGACCAACGUUGGGUCGUCGGGCCGGUCCCCGUCGAAGGCGGUCGCGGCCCGCGCUGCGGGUUCCACAGUGAGGCAGCG<br>UAAGAAUGCCUCCUGUGGAACCCGCAGCGCGGGCCGGACCACCUCGGCGGGGACAGGAGGGAUGUGGCGAUUCUACACCGAGGACUCGCCCGGCCUGAAGG<br>UCGGGCCAGUCCCGGUGCUCGUCAUGUCCCUCCUGUUCAUUGCGAGUGUGUUUAUGCUCCAUAUUUGGGGCAAAUACACUCGCAGU
	Ours<br>`-234.74 kcal/mol`<br>*-232.40 kcal/mol*	AUGCCCGGCCCGACGCCCAGCGGGACCAACGUUGGGUCGUCGGGCCGGUCCCCGUCGAAGGCGGUGGCGGCCCGCGCUGCGGGUUCCACAGUGAGGCAGCG<br>GAAGAAUGCCUCCUGUGGAACCCGCAGCGCGGGCCGCACCACCUCGGCGGGGACAGGAGGGAUGUGGCGAUUCUACACCGAGGACUCGCCCGGCCUGAAGG<br>UCGGGCCAGUCCCGGUGCUCGUCAUGUCCCUCCUGUUCAUUGCGAGUGUAUUUAUGCUCCAUAUUUGGGGCAAAUACACUCGCAGU
	LinearDesign<br>`-226.36 kcal/mol`<br>*-223.90 kcal/mol*	AUGGAGUCAAAGGAGGAGCUCGCCGCCAACAAUCUCAAUGGUGAGAACGCUCAGCAGGAGAAUGAGGGCGGCGAGCAGGCCCCCACCCAGAAUGAGGAGGA<br>GUCUCGUCAUCUGGGUGGGGGCGAGGGGCAGAAGCCGGGUGGUAAUAUCCGGCGCGGGCGUGUGAGACGGCUAGUGCCGAAUUUCCGCUGGGCGAUUCCGA<br>AUCGCCACAUCGAGCACAACGAAGCGCGCGACGACGUCGAGCGCUUCGUUGGCCAGAUGAUGGAGAUUAAGCGGAAGACGCGCGAGCAGCAGAUGCGGCAC<br>UACAUGCGGUUUCAGACGCCCGAGCCGGAUAACCACUACGACUUCUGCCUGAUUCCA
Q9NWD9	Random Walk<br>`-226.36 kcal/mol`<br>*-223.90 kcal/mol*	AUGGAGUCAAAGGAGGAGCUCGCCGCCAACAAUCUCAAUGGUGAGAACGCUCAGCAGGAGAAUGAGGGCGGCGAGCAGGCCCCCACCCAGAAUGAGGAGGA<br>GUCUCGUCAUCUGGGUGGGGGCGAGGGGCAGAAGCCGGGUGGUAAUAUCCGGCGCGGGCGUGUGAGACGGCUAGUGCCGAAUUUCCGCUGGGCGAUUCCGA<br>AUCGCCACAUCGAGCACAACGAAGCGCGCGACGACGUCGAGCGCUUCGUUGGCCAGAUGAUGGAGAUUAAGCGGAAGACGCGCGAGCAGCAGAUGCGGCAC<br>UAUAUGCGGUUUCAGACGCCCGAGCCGGAUAACCACUACGACUUCUGCCUGAUUCCA
	Ours<br>`-227.03 kcal/mol`<br>*-223.80 kcal/mol*	AUGGAAUCAAAGGAGGAGCUCGCCGCCAACAAUCUCAAUGGUGAGAACGCUCAGCAGGAGAAUGAGGGCGGCGAGCAGGCCCCCACCCAGAAUGAGGAGGA<br>GUCUCGUCAUCUGGGUGGGGGCGAGGGGCAGAAGCCGGGUGGUAAUAUCCGGCGCGGGCGUGUGAGGCGCUUAGUGCCGAAUUUCCGCUGGGCGAUUCCGA<br>AUCGCCACAUCGAGCACAACGAAGCGCGCGACGACGUCGAGCGCUUCGUUGGCCAGAUGAUGGAGAUUAAGCGGAAGACGCGCGAGCAGCAGAUGCGGCAC<br>UACAUGCGCUUUCAGACGCCCGAGCCGGAUAACCACUACGACUUCUGCCUGAUUCCA

Continued on next page

Table S5 – continued from previous page

ID	Method	mRNA Design
P14555	LinearDesign<br>-275.06 kcal/mol<br>*-273.60 kcal/mol*	AUGAAGACAUUGUUACUGCUGGCAGUGAUAAUGAUCUUCGGGCUCCUCCAGGCCCAUGGCAACUUGGUCAACUUUCAUAGGAUGAUCAAGUUGACCACGGG<br>CAAGGAGGCAGCCCUCUCGUACGGCUUCUACGGCUGUCACUGCGGCGUAGGUGGUCGCGGGAGCCCGAAGGACGCAACAGACCGCUGUUGCGUCACUCACG<br>ACUGCUGCUACAAGCGGUUGGAGAAGAGAGGUUGUGGGACAAAGUUUUUGUCCUACAAGUUCUCUAAUUCGGGCUCCCGGAUCACCUGCGCCAAGCAGGAC<br>AGCUGUAGAAGCCAGCUGUGCGAGUGCGACAAGGCGGCUGCCACCUGCUUUGCUCGGAAUAAGACUACGUACAACAAGAAGUACCAGUAUUAUUCCAACAA<br>GCAUUGCAGGGGCAGCACGCCUCGUUGC
	Random Walk<br>-275.06 kcal/mol<br>*-273.60 kcal/mol*	AUGAAGACAUUGUUACUGCUGGCAGUGAUAAUGAUCUUCGGGCUCCUCCAGGCCCAUGGCAACUUGGUCAACUUUCAUAGGAUGAUCAAGUUGACCACGGG<br>CAAGGAGGCAGCCCUCUCGUACGGCUUCUACGGCUGUCACUGCGGCGUAGGUGGUCGCGGGAGCCCGAAGGACGCAACAGACCGCUGUUGCGUCACUCACG<br>ACUGCUGCUACAAGCGGUUGGAGAAGAGAGGUUGUGGGACAAAGUUUUUGUCCUACAAGUUCUCUAAUUCGGGCUCCCGGAUCACCUGCGCCAAGCAGGAC<br>AGCUGUAGAAGCCAGCUGUGCGAGUGCGACAAGGCGGCUGCCACCUGCUUUGCUCGGAAUAAGACUACGUACAACAAGAAGUACCAGUAUUAUUCCAACAA<br>GCAUUGCAGGGGCAGCACGCCUCGUUGC
	Ours<br>-275.47 kcal/mol<br>*-273.40 kcal/mol*	AUGAAGACAUUGUUACUGUUAGCAGUGAUAAUGAUCUUCGGGCUGCUCCAGGCCCAUGGCAACUUGGUCAACUUUCAUAGGAUGAUCAAGUUGACCACGGG<br>CAAGGAGGCAGCCCUCUCGUACGGCUUCUACGGCUGUCACUGCGGCGUAGGUGGUCGCGGGAGCCCGAAGGACGCAACAGACCGCUGUUGCGUCACUCACG<br>ACUGCUGCUACAAGCGGUUGGAGAAGAGAGGUUGUGGGACAAAGUUUUUGUCCUACAAGUUCUCUAAUUCGGGCUCCCGGAUCACCUGCGCCAAGCAGGAC<br>AGCUGUAGAAGCCAGCUGUGCGAGUGCGACAAGGCGGCUGCCACCUGCUUUGCUAGGAAUAAGACUACGUACAAUAAGAAGUACCAGUAUUAUUCCAAUAA<br>GCAUUGCAGGGGCAGCACGCCUCGUUGC
Q8N111	LinearDesign<br>-335.89 kcal/mol<br>*-334.00 kcal/mol*	AUGGAGAGCCGGGGGAAGUCUGCGUCGUCACCUAAGCCGGACACGAAAGUCCCGCAGGUGACGACGGAGGCUAAGGUUCCCCCGGCUGCGGACGGGAAGGC<br>CCCCCUGACGAAGCCUUCUAAGAAGGAGGCUCCAGCGGAAAAGCAGCAGCCGCCCGCCGCCCCCACCACAGCGCCGGCGAAGAAGACCUCCGCGAAAGCGG<br>ACCCCGCCCUCCUGAACAACCACAGCAACCUGAAGCCCGCCCCGACGGUGCCGUCCUCCCCCGACGCCACCCCCGAGCCCAAGGGGCCGGGGGAUGGCGCG<br>GAGGAGGACGAGGCAGCGUCGGGGGGGCCAGGAGGGCGGGGUCCGUGGUCGUGCGAGAACUUCAACCCGCUGCUGGUGGCGGGCGGCGUGGCGGUUGCUGC<br>UAUUGCGCUGAUCUUAGGGGUGGCCUUCCUCGUCCGCAAGAAG
	Random Walk<br>-335.94 kcal/mol<br>*-333.20 kcal/mol*	AUGGAGAGCCGGGGGAAGUCUGCGUCGUCACCUAAGCCGGACACGAAAGUCCCGCAGGUGACGACGGAGGCUAAGGUUCCCCCGGCUGCGGACGGGAAGGC<br>CCCCCUGACGAAGCCUUCUAAGAAGGAGGCUCCAGCGGAAAAGCAGCAGCCGCCCGCCGCCCCCACCACAGCGCCGGCGAAGAAGACCUCCGCGAAAGCGG<br>ACCCCGCCCUCCUGAACAACCACAGCAACCUGAAGCCCGCCCCGACGGUGCCGUCCUCCCCCGACGCCACCCCCGAGCCCAAGGGGCCGGGGGAUGGCGCG<br>GAGGAGGACGAGGCAGCGUCGGGCGGGCCAGGAGGGCGGGGUCCGUGGUCGUGCGAGAACUUCAACCCGCUGCUGGUGGCGGGCGGCGUGGCGGUUGCUGC<br>UAUUGCGCUGAUCUUAGGGGUGGCCUUCCUCGUCCGCAAGAAG
	Ours<br>-336.40 kcal/mol<br>*-333.20 kcal/mol*	AUGGAGAGCCGGGGGAAGUCUGCGUCGUCACCUAAGCCGGACACGAAAGUCCCGCAGGUGACGACGGAGGCUAAGGUUCCCCCGGCUGCGGACGGGAAGGC<br>CCCCCUGACGAAGCCUUCUAAGAAGGAGGCUCCAGCGGAAAAGCAGCAGCCGCCCGCCGCCCCCACCACAGCGCCGGCGAAGAAGACCUCCGCGAAAGCGG<br>ACCCCGCCCUCCUGAACAAUCAUUCAAAUUUGAAGCCCGCCCCGACGGUGCCGUCCUCCCCCGACGCCACCCCCGAGCCCAAGGGGCCGGGGGAUGGCGCG<br>GAGGAGGACGAGGCAGCGUCGGGCGGGCCAGGAGGGCGGGGUCCGUGGUCGUGCGAGAACUUCAACCCGCUGUUGGUGGCGGGCGGCGUGGCGGUUGCUGC<br>UAUUGCGCUGAUCUUAGGGGUGGCCUUCCUCGUCCGCAAGAAG
P63125	LinearDesign<br>-299.18 kcal/mol<br>*-296.20 kcal/mol*	UGGGCCAGUCAGGUCUCUGAGAAUCGGCCAGUUUGCAAGGCCAUUAUUCAGGGCAAGCAGUUCGAGGGGUUGGUCGACACCGGGGCCGACGUCUCGAUCAU<br>UGCCCUGAAUCAGUGGCCUAAGAACUGGCCGAAGCAGAAGGCCGUGACUGGCCUUGUUGGAGUAGGCACGGCCUCAGAGGUUUAUCAGUCGACGGAGAUCC<br>UGCACUGUCUGGGUCCUGACAAUCAGGAGUCGACAGUGCAGCCCAUGAUCACGUCGAUUCCGUUGAACCUCUGGGGCCGUGACCUGCUCCAGCAGUGGGGA<br>GCGGAGAUCACGAUGCCCGCCCCGCUGUAUAGCCCAACUUCGCAGAAGAUCAUGACGAAGAUGGGCUAUAUCCCUGGUAAGGGUCUUGGCAAGAACGAGGA<br>CGGGAUCAAGAUUCCCGUCGAGGCCAAGAUCAACCAGAAGCGGGAGGGCAUCGGAUAUCCGUUC
	Random Walk<br>-299.60 kcal/mol<br>*-296.20 kcal/mol*	UGGGCCAGUCAGGUCUCUGAGAAUCGGCCAGUUUGUAAGGCCAUUAUUCAGGGCAAGCAGUUCGAGGGGUUGGUCGACACCGGGGCCGACGUCUCGAUCAU<br>UGCCCUGAAUCAGUGGCCUAAGAACUGGCCGAAGCAGAAGGCCGUGACUGGCCUUGUUGGAGUAGGCACGGCCUCAGAGGUUUAUCAGUCGACGGAGAUCC<br>UGCACUGUCUGGGUCCUGAUAAUCAGGAGUCGACAGUGCAGCCGAUGAUCACGUCGAUUCCGUUGAACCUCUGGGGCCGUGACCUGCUCCAGCAGUGGGGA<br>GCGGAGAUCACGAUGCCCGCCCCGCUGUAUAGCCCAACUUCGCAGAAGAUCAUGACGAAGAUGGGCUAUAUACCUGGUAAGGGUCUUGGCAAGAACGAGGA<br>CGGGAUUAAGAUUCCCGUCGAGGCCAAGAUCAACCAGAAGCGGGAGGGCAUCGGAUAUCCGUUC
	Ours<br>-299.59 kcal/mol<br>*-295.70 kcal/mol*	UGGGCCAGUCAGGUCUCUGAGAAUCGGCCAGUUUGUAAGGCCAUUAUUCAGGGCAAGCAGUUCGAGGGGUUGGUCGACACCGGGGCCGACGUCUCGAUCAU<br>UGCCCUGAAUCAGUGGCCUAAGAACUGGCCGAAGCAGAAGGCCGUGACUGGCCUUGUUGGAGUAGGCACGGCCUCAGAGGUUUACCAGUCGACGGAGAUCC<br>UGCACUGUCUGGGUCCUGAUAAUCAGGAGUCGACAGUGCAGCCGAUGAUCACGUCGAUUCCGUUGAACCUCUGGGGCCGUGACCUGCUCCAGCAAUGGGGA<br>GCGGAGAUCACGAUGCCCGCCCCGCUGUAUAGCCCAACUUCGCAGAAGAUCAUGACGAAGAUGGGCUAUAUACCUGGUAAGGGUCUUGGCAAGAACGAAGA<br>CGGGAUAAAGAUUCCCGUCGAGGCCAAGAUCAACCAGAAGCGGGAGGGCAUCGGAUAUCCGUUC
Q6XD76	LinearDesign<br>-427.19 kcal/mol<br>*-424.40 kcal/mol*	AUGGAGACUCGGAAGCCGGCGGAGCGCCUUGCUCUCCCCUACAGUCUGAGGACCGCUCCGCUCGGCGUUCCGGGGACACUGCCCGGGCUGCCCCGUCGAGA<br>CCCCCUGCGCGUGGCGCUCCGGCUCGAUGCCGCUUGCUGGGAGUGGGCUCGAUCGGGCUGUGCGCGGGGCUGGCAGUACCUGCCAGUCCCGCUGGACAGCG<br>CGUUCGAGCCCGCGUUCCUACGCAAGCGGAACGAGCGGGAGCGCCAGCGGGUGCGUUGUGUGAACGAGGGCUACGCCCGCCUGCGUGACCACCUGCCUCGA<br>GAGCUCGCUGACAAGCGGCUCUCGAAGGUGGAGACGCUGCGGGCGGCCAUCGAUUACAUAAAGCACCUGCAGGAGCUCCUGGAGCGCCAGGCGUGGGGUCU<br>CGAGGGGGCAGCCGGGGCAGUGCCCCAGCGCCGAGCGGAGUGCAACUCAGACGGGGAGAGCAAGGCGUCGUCGGCUCCGAGUCCCAGCUCCGAGCCCGAGG<br>AGGGCGGGAGC
	Random Walk<br>-427.50 kcal/mol<br>*-424.40 kcal/mol*	AUGGAGACUCGGAAGCCGGCGGAGCGCCUUGCUCUCCCCUACAGUCUGAGGACCGCUCCGCUCGGCGUUCCGGGGACACUGCCCGGGCUGCCCCGUCGAGA<br>CCCCCUGCGCGUGGCGCUCCGGCUCGAUGCCGCUUGCUGGGAGUGGGCUCGAUCGGGCUGUGCGCGGGGCUGGCAGUAUCUGCCAGUCCCGCUGGACAGCG<br>CGUUCGAGCCCGCUUUCCUACGCAAGCGGAACGAGCGGGAGCGCCAGCGGGUGCGUUGUGUGAACGAGGGCUACGCCCGCCUGCGUGACCACCUGCCUCGA<br>GAGCUCGCUGACAAGCGGCUCUCGAAGGUGGAGACGCUGCGGGCGGCCAUCGAUUACAUAAAGCACCUGCAGGAGCUCCUGGAGCGCCAGGCGUGGGGUCU<br>CGAGGGGGCAGCCGGGGCAGUGCCCCAGCGCCGAGCGGAGUGCAACUCAGACGGGGAGAGCAAGGCGUCGUCGGCUCCGAGUCCCAGCUCCGAGCCCGAGG<br>AGGGCGGGAGC
	Ours<br>-427.82 kcal/mol<br>*-424.40 kcal/mol*	AUGGAGACUCGGAAGCCGGCGGAGCGCCUUGCUCUCCCCUACAGUCUGAGGACCGCUCCGCUCGGCGUUCCGGGGACACUGCCCGGGCUGCCCCGUCGAGA<br>CCCCCUGCGCGUGGCGCUCCGGCUCGAUGCCGCUUGCUGGGAGUGGGCUCGAUCGGGCUGUGCGCGGGGCUGGCAGUACCUGCCAGUCCCGCUGGACAGCG<br>CGUUCGAGCCCGCUUUCCUACGCAAGCGGAACGAGCGGGAGCGCCAGCGGGUGCGUUGUGUGAACGAGGGCUACGCCCGCCUGCGUGACCACCUUCCUCGA<br>GAGCUCGCUGACAAGCGGCUCUCGAAGGUGGAGACGCUGCGGGCGGCCAUCGAUUACAUAAAGCACCUGCAGGAGCUCCUGGAGCGCCAGGCGUGGGGUCU<br>CGAGGGGGCAGCCGGGGCAGUGCCCCAGCGCCGAGCGGAGUGUAACUCAGACGGGGAGAGCAAGGCGUCGUCGGCUCCGAGUCCCAGCUCCGAGCCCGAGG<br>AGGGCGGGAGC

Continued on next page

Table S5 – continued from previous page

ID	Method	mRNA Design
	LinearDesign<br>-361.48 kcal/mol<br>*-359.40 kcal/mol*	AUGACUGAUAAGACGGAGAAGGUCGCAGUGGACCCCGAGACCGUGUUCAAACGGCCUCGGGAGUGCGACUCUCCGUCUUAUCAGAAGAGGCAGCGGAUGGC<br>GUUGCUGGCCCGCAAGCAGGGGGCCGGCGACAGCCUGAUCGCGGGGUCCGCCAUGAGCAAGGAGAAGAAGCUCAUGACGGGCCACGCGAUCCCUCCUUCAC<br>AGCUUGACAGUCAGAUCGAUGAUUUCACUGGCUUUUCGAAGGACGGCAUGAUGCAGAAGCCGGGAAGCAAUGCCCCGGUAGGCGGGAACGUCACCAGUAAC<br>UUCUCUGGUGACGAUCUCGAAUGCCGGGGCAUUGCUUCCUCCCCGAAGAGCCAGCAGGAGAUCAACGCUGACAUCAAGUGUCAAGUUGUGAAGGAGAUCCG<br>CUGCCUCGGUCGUAAGUAUGAGAAGAUUUUUGAGAUGCUUGAGGGAGUCCAGGGGCCGACUGCAGUGCGCAAGCGCUUCUUUGAGAGCAUCAUCAAAGAAG<br>CGGCGCGCUGCAUGCGGCGGGACUUCGUCAAGCAUCUCAAGAAGAAGCUCAAGCGUAUGAUC
P0DMU9	Random Walk<br>-362.10 kcal/mol<br>*-359.40 kcal/mol*	AUGACUGAUAAGACGGAGAAGGUCGCAGUCGAUCCCGAGACCGUGUUUAAACGGCCUCGGGAGUGCGACUCUCCGUCUUAUCAGAAGAGGCAGCGGAUGGC<br>GUUGCUGGCCCGCAAGCAGGGGGCCGGCGACAGCCUGAUCGCGGGGUCCGCCAUGAGCAAGGAGAAGAAGCUCAUGACGGGCCACGCGAUCCCUCCUUCAC<br>AGCUUGACAGUCAGAUCGAUGAUUUCACUGGCUUUUCGAAGGACGGCAUGAUGCAAAAGCCGGGAAGCAAUGCCCCGGUAGGCGGGAACGUCACCAGUAAU<br>UUCUCUGGUGACGAUCUCGAAUGCCGGGGCAUUGCUUCCUCCCCGAAGAGCCAGCAGGAGAUCAACGCUGACAUCAAGUGUCAAGUUGUGAAGGAGAUCCG<br>CUGCCUCGGUCGUAAGUAUGAGAAGAUUUUUGAGAUGCUUGAGGGAGUCCAGGGGCCGACUGCAGUGCGCAAGCGCUUCUUUGAGAGCAUCAUCAAAGAAG<br>CGGCGCGCUGCAUGCGGCGGGACUUCGUCAAGCAUCUCAAGAAGAAGCUCAAGCGUAUGAUC
	Ours<br>-362.12 kcal/mol<br>*-359.40 kcal/mol*	AUGACUGAUAAGACGGAGAAGGUCGCAGUCGAUCCCGAGACCGUGUUCAAACGGCCUCGGGAGUGCGACUCUCCGUCUUAUCAGAAGAGGCAGCGGAUGGC<br>GUUGCUGGCCCGCAAGCAGGGGGCCGGCGACAGCCUGAUCGCGGGGUCCGCCAUGAGCAAGGAGAAGAAGCUCAUGACGGGCCACGCGAUCCCUCCUUCAC<br>AGCUUGACAGUCAGAUCGAUGAUUUCACUGGCUUUUCGAAGGACGGCAUGAUGCAAAAGCCGGGAAGCAAUGCCCCGGUAGGCGGGAACGUCACCAGUAAU<br>UUUUCUGGUGACGAUCUCGAAUGCCGGGGCAUUGCUUCCUCCCCGAAGAGCCAGCAGGAGAUCAACGCUGACAUCAAGUGUCAAGUUGUGAAGGAGAUCCG<br>CUGCCUCGGUCGUAAGUAUGAGAAGAUUUUUGAGAUGCUUGAGGGAGUCCAGGGGCCGACUGCAGUGCGCAAGCGCUUCUUUGAGAGUAUCAUCAAAGAAG<br>CGGCGCGCUGCAUGCGGCGGGACUUCGUCAAGCAUCUCAAGAAGAAGCUCAAGCGUAUGAUC
	LinearDesign<br>-545.55 kcal/mol<br>*-542.90 kcal/mol*	AUGUUCGUGCGGCCGGAGUCCGGAGAGCAAGGCCCGGAGACGCUGGCUCCAGCGAGCGGGGCCGAGAUCCAGCGGUUCCCGGUUCCGGCGGUUGAGCCUGU<br>GCCGGCCCCGGGCGCCGACAGCCCCCCCGGGACGGCUCUGGAGCUUGAGGAAGCUCCAGAGCCGUCCUGUCGGUGCCCGGGGACGGCACAGGAUCAGCCGU<br>CGGAGGAGCUGCCGGAUUUCAUGGCCCCGCCGGUGGAGCCACCAGCUUCCGCUCUGGAGCUCAAGGUCUGGCUGGAGUUGGAAGUUGCUGAGAGGGGGGG<br>CAGCACUCCUCCUCUCAGCAGCUUCCACACUGCAGCCAGUCUUGGGCUCAGUGGAAGCUGUGGCGCCAGCGUCCGGGCUUUGCUAUCUGGGCUCCGCUGCC<br>GCACUGGCGGGGGACGAGCCUGAUCCAGCAGAGCUCGUCCCCCGCCGCAGAGGGCCCAGCUGCCACAGCUGCGGGCGCCGUCUGUCUCCCGGCGGGGGGG<br>CCGGCGAGCAGGAGAAGGAGCCCGUGAGCCGGGGGAGCUCACGGAGCUCCUGCUCCCAGCGCCGGCCCCCCCGCCGGGGAUGGAGGUGUGCCCGCAGCUG<br>GGCAUCUGGGCCAUCUGCCCG
P0DPF6	Random Walk<br>-545.97 kcal/mol<br>*-542.90 kcal/mol*	AUGUUCGUGCGGCCGGAGUCCGGAGAGCAAGGCCCGGAGACGCUGGCUCCAGCGAGCGGGGCCGAGAUCCAGCGGUUCCCGGUUCCGGCGGUUGAGCCUGU<br>GCCGGCCCCGGGCGCCGACAGCCCGCCCGGGACGGCUCUGGAGCUUGAGGAAGCUCCAGAGCCGUCCUGUCGGUGCCCGGGGACGGCACAGGAUCAGCCGU<br>CGGAGGAGCUGCCGGAUUUCAUGGCCCCGCCGGUGGAGCCACCAGCUUCCGCUCUUGAGCUCAAGGUCUGGCUGGAGUUGGAAGUUGCUGAGAGGGGGGG<br>CAGCACUCCUCCUCUCAGCAGCUUCCACACUGCAGCCAGUCUUGGGCUCAGUGGAAGCUGUGGCGCCAGCGUCCGGGCUUUGCUAUCUGGGCUCCGCUGCC<br>GCACUGGCGGGGGACGAGCCUGAUCCAGCAGAGCUCGUCCCCCGCCGCAGAGGGCCCAGCUGCCACAGCUGCGGGCGCCGUCUGUCUCCCGGCGGGGGGG<br>CCGGCGAGCAGGAGAAGGAGCCCGUGAGCCGGGGGAGCUCACGGAGCUCCUGCUCCCAGCGCCGGCCCCCCCGCCGGGGAUGGAGGUGUGCCCGCAGCUG<br>GGCAUCUGGGCCAUCUGCCCG
	Ours<br>-546.07 kcal/mol<br>*-542.90 kcal/mol*	AUGUUCGUGCGGCCGGAGUCCGGAGAGCAAGGCCCGGAGACGCUGGCUCCAGCGAGCGGGGCCGAGAUCCAGCGGUUCCCGGUUCCGGCGGUUGAGCCUGU<br>GCCGGCCCCGGGCGCCGACAGCCCCCCCGGGACGGCUCUGGAGCUUGAGGAAGCUCCAGAGCCGUCCUGUCGGUGCCCGGGGACGGCACAGGAUCAGCCGU<br>CGGAGGAACUGCCGGAUUUCAUGGCCCCGCCGGUGGAGCCACCAGCUUCCGCUCUUGAGCUCAAGGUCUGGCUGGAGUUGGAAGUUGCUGAGAGGGGGGG<br>CAACACUCCUCCUCUCAGCAGCUUCCACACUGCAGCCAGUCUUGGGCUCAGUGGAAGCUGUGGCGCCAGCGUCCGGGCUUUGCUAUCUGGGCUCCGCUGCC<br>GCACUGGCGGGGGACGAGCCUGAUCCAGCAGAGCUCGUCCCCGCGGCAGAGGGCCCAGCUGCCACAGCUGCGGGCGCCGUCUGUCUCCCGGCGGGGGGG<br>CCGGCGAGCAGGAGAAGGAGCCCGUGAGCCGGGGGAGCUCACGGAGCUCCUGCUCCCAGCGCCGGCCCCCCCGCCGGGGAUGGAGGUGUGCCCGCAGCUG<br>GGCAUCUGGGCCAUCUGCCCG
	LinearDesign<br>-532.98 kcal/mol<br>*-530.00 kcal/mol*	AUGGCCGAGCUGUACGUGAAGCCCGGGAACAAGGAGCGGGGGUGGAACGAUCCACCCCAGUUCUCGUAUGGUCUGCAGACGCAGGCCGGCGGUCCUCGUCG<br>CAGCCUCCUCACUAAGCGGGUGGCCGCUCCUCAGGAUGGGUCUCCGCGUGUGCCCGCGAGCGAGACUUCUCCAGGGCCUCCUCCAAUGGGGCCGCCGCCGC<br>CGUCCUCCAAGGCCCCUCGCUCUCCACCGGUAGGAAGUGGGCCCGCCUCGGGGGUGGAGCCCACUUCCUUCCCGGUGGAGAGCGAGGCGGUCAUGGAGGAC<br>GUGCUGCGGCCCCUGGAGCAGGCCCUGGAGGAUUGCCGCGGGCACACGCGGAAGCAGGUGUGCGACGACAUUUCCCGCCGGCUCGCGCUCCUGCAGGAGCA<br>GUGGGCCGGCGGGAAGUUGUCCAUACCUGUCAAGAAGCGCAUGGCGCUUCUUGUGCAGGAGCUUAGCUCCCACCGCUGGGACGCCGCCGAUGACAUCCACC<br>GUAGCCUUAUGGUGGACCAUGUCACGGAGGUGUCCCAGUGGAUGGUGGGAGUUAAGCGACUCAUCGCUGAGAAGCGGUCCCUGUUUAGUGAGGAGGCUGCG<br>AACGAGGAGAAGUCGGCCGCGACUGCAGAGAAGAACCAUACGAUCCCGGGCUUCCAGCAGGCCAGC
Q9HD15	Random Walk<br>-533.19 kcal/mol<br>*-530.00 kcal/mol*	AUGGCCGAGCUGUACGUGAAGCCCGGGAACAAGGAGCGGGGGUGGAAUGAUCCACCCCAGUUCUCGUAUGGUCUGCAGACGCAGGCCGGCGGUCCUCGUCG<br>CAGCCUCCUCACUAAGCGGGUGGCCGCUCCUCAGGAUGGGUCUCCGCGUGUGCCCGCGAGCGAGACUUCUCCAGGGCCUCCUCCAAUGGGGCCGCCGCCGC<br>CGUCCUCCAAGGCCCCUCGCUCUCCACCGGUGGGAAGUGGGCCCGCCUCAGGGUGGAGCCCACUUCCUUCCCGGUGGAGAGCGAGGCGGUCAUGGAGGAC<br>GUGCUGCGGCCCCUGGAGCAGGCCCUGGAGGAUUGCCGCGGGCACACGCGGAAGCAGGUGUGCGACGACAUUUCCCGCCGGCUCGCGCUCCUGCAGGAGCA<br>GUGGGCCGGCGGGAAGUUGUCCAUACCUGUCAAGAAGCGCAUGGCGCUUCUUGUCCAGGAGCUUAGCUCCCACCGCUGGGACGCCGCCGAUGACAUCCACC<br>GUAGCCUUAUGGUGGAUCAUGUCACGGAGGUGUCCCAGUGGAUGGUGGGAGUUAAGCGACUCAUCGCUGAGAAGCGGUCCCUGUUUAGUGAGGAGGCUGCG<br>AACGAGGAGAAGUCGGCCGCGACUGCAGAGAAGAACCAUACGAUCCCGGGCUUCCAGCAGGCCAGC
	Ours<br>-533.30 kcal/mol<br>*-530.00 kcal/mol*	AUGGCCGAGCUGUAUGUGAAGCCCGGGAAUAAGGAACGGGGGUGGAACGAUCCACCCCAGUUCUCGUAUGGUCUGCAGACGCAGGCCGGCGGUCCUCGUCG<br>CAGCCUCCUCACUAAACGGGUGGCCGCUCCUCAGGAUGGGUCUCCGCGUGUGCCCGCGAGCGAGACUUCUCCAGGGCCUCCUCCAAUGGGGCCGCCGCCAC<br>CGUCCUCCAAGGCCCCUCGCUCUCCACCGGUAGGAAGUGGGCCCGCCUCGGGGGUGGAGCCCACUUCCUUCCCGGUGGAGAGCGAGGCGGUCAUGGAGGAC<br>GUGCUGCGGCCCCUGGAGCAGGCCCUGGAGGAUUGCCGCGGGCACACGCGGAAGCAGGUGUGCGACGACAUUUCCCGCCGGCUCGCGCUCCUGCAGGAGCA<br>GUGGGCCGGCGGGAAGUUGUCCAUACCUGUCAAGAAGCGCAUGGCGCUUCUUGUGCAGGAGCUUAGCUCCCACCGCUGGGACGCCGCCGAUGACAUCCACC<br>GUAGUCUUAUGGUGGAUCAUGUCACGGAGGUGUCCCAGUGGAUGGUGGGAGUUAAGCGACUCAUCGCUGAGAAGCGGUCCCUGUUUAGUGAGGAGGCUGCG<br>AACGAGGAGAAGUCGGCCGCGACUGCAGAGAAGAACCAUACGAUCCCGGGCUUCCAGCAGGCCAGU

Continued on next page

Table S5 – continued from previous page

ID	Method	mRNA Design
Q6T310	LinearDesign<br>-504.29 kcal/mol<br>*-500.90 kcal/mol*	AUGCGGCCGCUCUCCAUGAGCGGACAUUUCCUGCUCGCCCCGAUCCCCGAGAGCUCCAGCGAUUACCUUCUGCCGAAGGAUAUCAAGCUGGCAGUUCUCGG<br>GGCGGGGCGAGUAGGAAAGUCCGCCAUGAUCGUACGUUUUCUGACGAAGAGAUUUAUCGGGGACUACGAGCCCAACACGGGCAAGCUGUAUUCCCGUCUUG<br>UGUAUGUCGAGGGUGAUCAGCUGAGCCUUCAGAUUCAGGAUACACCUGGGGGUGUGCAGAUACAGGACAGCCUGCCUCAGGUAGUUGACUCGUUGUCCAAG<br>UGCGUGCAGUGGGCCGAGGGUUUUCUCUUGGUCUACAGCAUCACGGACUACGACAGCUACCUGAGCAUCAGGCCCCUGUAUCAGCACAUCCGCAAGGUGCA<br>UCCUGAUUCGAAGGCUCCGGUGAUCAUCGUCGGCAACAAGGGGGACCUGCUGCACGCGCGGCAGGUCCAGACACAAGACGGGAUACAGCUUGCCAACGAGU<br>UGGGCUCGUUGUUCCUGGAGAUCUCUACGUCAGAGAACUACGAGGACGUCUGUGACGUCUUCCAGCACCUCUGCAAAGAGGUGUCGAAGAUGCACGGACUG<br>UCCGGAGAGCGGCGGCGUGCGUCGAUCAUCCCAAGGCCGCGGAGCCCUAACAUGCAGGACUUGAAGCGCCGCUUCAAGCAGGCGCUGAGUCCUAAGGUUAA<br>GGCUCCGUCGGCCUUGGGA
	Random Walk<br>-504.53 kcal/mol<br>*-500.90 kcal/mol*	AUGCGGCCGCUCUCCAUGAGCGGACAUUUCCUGCUCGCCCCGAUCCCCGAGAGCUCCAGCGAUUACCUUCUGCCGAAGGAUAUCAAGCUGGCAGUUCUCGG<br>GGCGGGGCGAGUAGGAAAGUCCGCCAUGAUCGUACGUUUUCUGACGAAGAGAUUUAUCGGGGACUACGAGCCCAACACGGGCAAGCUGUAUUCCCGUCUUG<br>UGUAUGUCGAGGGUGAUCAGCUGAGCCUUCAGAUUCAGGAUACACCUGGGGGUGUGCAGAUACAGGACAGCCUGCCUCAGGUAGUUGACUCGUUGUCCAAG<br>UGUGUGCAGUGGGCCGAGGGUUUUCUCUUGGUCUACAGCAUCACGGACUACGACAGCUACCUGAGCAUCAGGCCCCUGUAUCAGCACAUCCGCAAGGUGCA<br>UCCUGAUUCGAAGGCUCCGGUGAUCAUCGUCGGCAACAAGGGGGACCUGCUGCACGCGCGGCAGGUCCAGACACAAGACGGGAUACAGCUUGCCAACGAGU<br>UGGGCUCGUUGUUCCUGGAGAUCUCUACGUCAGAGAACUACGAGGACGUCUGUGACGUCUUCCAGCACCUCUGCAAAGAGGUGUCGAAGAUGCACGGACUG<br>UCCGGAGAGCGGCGGCGUGCGAGCAUCAUCCCAAGGCCGCGGAGCCCUAACAUGCAGGACUUAAAGCGCCGCUUCAAGCAGGCGCUGAGUCCUAAGGUUAA<br>GGCUCCGUCGGCCUUGGGA
	Ours<br>-504.82 kcal/mol<br>*-500.10 kcal/mol*	AUGCGGCCGCUCUCCAUGAGCGGACAUUUCCUGCUCGCCCCGAUCCCCGAGAGCAGCAGCGAUUACCUUCUGCCGAAGGAUAUCAAGCUGGCUGUUCUCGG<br>GGCGGGGCGAGUAGGAAAGUCCGCCAUGAUCGUACGUUUUCUGACGAAGAGAUUUAUCGGGGACUACGAGCCCAACACGGGCAAGCUGUAUUCCCGUCUUG<br>UGUAUGUCGAGGGUGAUCAGCUGAGCCUUCAGAUUCAGGAUACACCUGGGGGUGUGCAGAUACAGGACAGCCUGCCUCAGGUAGUUGAUUCGUUGUCCAAG<br>UGUGUGCAGUGGGCCGAGGGUUUUCUCUUGGUCUACAGCAUCACGGACUACGACAGCUACCUGAGCAUCAGGCCCCUGUAUCAGCACAUCCGCAAGGUGCA<br>UCCUGAUUCGAAGGCUCCGGUGAUCAUCGUCGGCAAUAAGGGGGACCUGCUGCACGCGCGGCAGGUCCAGACACAAGACGGGAUACAGCUUGCCAACGAGU<br>UGGGCUCGUUGUUCCUGGAGAUCUCUACGUCAGAGAACUACGAGGACGUCUGUGACGUCUUCCAGCACCUCUGCAAAGAGGUGUCGAAGAUGCACGGACUG<br>UCCGGAGAGCGGCGGCGUGCGAGCAUCAUCCCAAGGCCGCGGAGCCCUAACAUGCAGGACUUGAAGCGCCGCUUUAAGCAGGCGCUCAGUCCUAAGGUUAA<br>GGCUCCGUCGGCCUUGGGA
Q9BRP0	LinearDesign<br>-586.50 kcal/mol<br>*-583.70 kcal/mol*	AUGCCGAAGGUCUUCCUCGUUAAGCGACGUAGUCUCGGGGUGAGCGUUCGGAGCUGGGACGAGCUCCCCGACGAGAAGAGGGCCGACACGUACAUUCCGGU<br>AGGCCUCGGGCGGCUCUUGCAUGACCCGCCCGAGGACUGCCGGAGUGACGGUGGGUCCUCUUCUGGGUCGGGGAGCUCGUCCGCGGGGGAGCCCGGGGGGG<br>CCGAGUCGUCCUCGUCCCCCCACGCUCCGGAGUCGGAGACUCCGGAGCCGGGGGACGCGGAGGGCCCCGAUGGCCACCUCGCCACGAAGCAGCGUCCCGUC<br>GCCCGUAGUAAAAUCAAGUUUACUACGGGUACGUGUAGUGACUCUGUUGUACACUCGUGCGAUCUUUGUGGGAAGGGAUUCCGGCUGCAGCGCAUGCUGAA<br>CAGGCACUUGAAGUGCCACAAUCAGGUCAAGCGUCAUCUUUGCACCUUUUGCGGCAAAGGGUUCAAUGAUACGUUUGACCUGAAGCGACACGUUCGCACUC<br>AUACGGGCAUCCGCCCGUAUAAGUGCAACGUGUGCAACAAGGCGUUCACGCAGCGCUGCAGCCUGGAGUCCCAUCUCAAGAAGAUCCACGGUGUGCAGCAG<br>CAGUACGCGUACAAGCAGCGACGGGACAAGCUGUACGUGUGCGAGGACUGUGGCUAUACGGGGCCCACACAGGAGGACCUGUACCUCCAUGUGAACUCGGC<br>CCACCCGGGCUCCUCCUUCCUGAAGAAGACCAGCAAGAAGCUUGCUGCUCUUCUUCAGGGAAAGCUCACGAGCGCUCACCAAGAGAAUACGUCGCUUAGCG<br>AGGAAGAGGAGCGGAAG
	Random Walk<br>-586.84 kcal/mol<br>*-583.70 kcal/mol*	AUGCCGAAGGUCUUCCUCGUUAAGCGACGUAGUCUCGGGGUGAGCGUUCGGAGCUGGGACGAGCUCCCCGACGAGAAGAGGGCCGACACGUACAUUCCGGU<br>AGGCCUCGGGCGGCUCUUGCAUGACCCGCCCGAGGACUGCCGGAGUGACGGUGGGUCCUCUUCUGGGUCGGGGAGCUCGUCCGCGGGGGAGCCCGGGGGGG<br>CCGAGUCCUCCUCGUCCCCCCACGCUCCGGAGUCGGAGACUCCGGAGCCGGGGGACGCGGAGGGCCCCGAUGGCCACCUCGCCACGAAGCAGCGUCCCGUC<br>GCCCGUAGUAAAAUCAAGUUUACUACGGGUACGUGUAGUGACUCUGUUGUACACUCGUGCGAUCUUUGUGGGAAGGGAUUCCGGCUGCAGCGCAUGCUGAA<br>CAGGCACUUGAAGUGCCACAAUCAGGUCAAGCGUCAUCUUUGCACCUUUUGCGGCAAAGGGUUCAAUGAUACGUUUGACCUGAAGCGACACGUUCGCACUC<br>AUACGGGCAUCCGCCCGUAUAAGUGCAACGUGUGCAACAAGGCGUUCACGCAGCGCUGCAGCCUGGAGUCCCAUCUCAAGAAGAUCCACGGUGUGCAGCAG<br>CAGUACGCGUACAAGCAGCGACGGGACAAGCUGUACGUGUGCGAGGACUGUGGCUAUACGGGGCCCACACAGGAGGAUCUGUACCUCCAUGUGAACUCGGC<br>CCACCCGGGCUCCUCCUUCCUGAAGAAGACCAGCAAGAAGCUUGCUGCUCUUCUUCAGGGAAAGCUCACGAGCGCUCACCAAGAGAAUACGUCGCUUAGCG<br>AGGAAGAGGAGCGGAAG
	Ours<br>-583.20 kcal/mol<br>*-583.20 kcal/mol*	AUGCCGAAGGUCUUCCUCGUUAAGCGACGUAGUCUCGGGGUGAGCGUUCGGAGCUGGGACGAGCUCCCCGACGAGAAGAGGGCCGACACGUACAUUCCGGU<br>AGGCCUCGGGCGGUUGUUGCACGACCCGCCCGAGGACUGCCGGAGUGACGGUGGGUCCUCUUCUGGGUCGGGGAGCUCGUCCGCGGGGGAGCCCGGGGGGG<br>CCGAGUCCUCCUCGUCCCCCCACGCUCCGGAGUCGGAGACUCCGGAGCCGGGGGACGCGGAGGGCCCCGAUGGCCACCUCGCCACGAAGCAGCGUCCCGUC<br>GCCCGUAGUAAAAUCAAGUUUACUACGGGUACGUGUAGUGACUCUGUUGUACACUCGUGUGAUCUUUGUGGGAAGGGAUUCCGGCUGCAGCGCAUGCUGAA<br>CCGGCACUUGAAGUGCCAUAAUCAGGUCAAGCGUCAUCUUUGUACCUUUUGCGGCAAAGGGUUCAAUGAUACGUUUGACCUGAAGCGACACGUUCGCACUC<br>AUACGGGCAUCCGCCCGUAUAAGUGCAACGUGUGCAACAAGGCGUUCACGCAGCGCUGCAGCCUGGAGUCCCAUCUCAAGAAGAUCCACGGUGUGCAGCAG<br>CAGUACGCGUACAAGCAGCGACGGGACAAGCUGUACGUGUGCGAGGAUUGUGGCUAUACGGGGCCCACACAGGAGGAUUUAUACCUCCAUGUGAACUCGGC<br>CCACCCGGGCUCCUCCUUCCUGAAGAAGACCAGCAAGAAGCUUGCUGCUCUUCUUCAGGGAAAGCUCACGAGCGCUCACCAAGAGAAUACGUCGCUUAGCG<br>AGGAAGAGGAGCGGAAG

Continued on next page

Table S5 – continued from previous page

ID	Method	mRNA Design
P56178	LinearDesign<br>-606.58 kcal/mol<br>*-602.80 kcal/mol*	AUGACUGGUGUGUUCGAUCGGCGGGUGCCGAGCAUCCGGAGUGGUGACUUUCAGGCUCCGUUCCAGACCUCUGCGGCUAUGCACCAUCCGUCUCAGGAAUC<br>UCCUACUCUGCCUGAGUCGUCGGCGACGGAUUCCGAUUAUUACUCCCCGACUGGUGGUGCGCCGCACGGGUACUGUAGUCCGACGUCGGCCUCGUACGGUA<br>AGGCCCUGAACCCGUAUCAGUACCAGUACCAUGGUGUGAACGGGUCGGCAGGGUCUUACCCUGCGAAGGCCUACGCGGACUACAGCUACGCGUCGUCGUAC<br>CACCAGUACGGGGGGGCGUAUAAUCGGGUUCCGUCGGCGACGAAUCAGCCAGAGAAGGAGGUGACUGAGCCGGAGGUGCGUAUGGUCAAUGGAAAGCCGAA<br>GAAGGUCCGCAAGCCGAGGACGAUCUAUUCGUCCUUCCAGCUUGCGGCCCUUCAGCGGCGUUUCCAGAAGACCCAGUAUCUCGCACUGCCGGAGAGGGCGG<br>AGCUGGCUGCAAGCCUGGGGCUCACUCAGACGCAGGUGAAGAUCUGGUUCCAGAACAAGCGUUCUAAGAUCAAGAAGAUCAUGAAGAACGGUGAGAUGCCG<br>CCAGAGCAUUCACCUUCGUCGAGUGACCCCAUGGCUUGCAACAGCCCGCAAUCUCCGGCAGUGUGGGAGCCGCAGGGGUCUUCGCGGAGCCUGAGUCACCA<br>CCCGCAUGCGCACCCGCCGACGAGCAACCAGUCACCAGCGAGCUCGUACCUGGAGAAUAGUGCGAGUUGGUACACUAGCGCGGCUAGUAGUAUCAACUCGC<br>ACUUGCCUCCACCGGGCUCGCUGCAGCACCCGCUGGCGCUGGCCAGCGGGACGCUGUAC
	Random Walk<br>-607.04 kcal/mol<br>*-602.80 kcal/mol*	AUGACUGGUGUGUUCGAUCGGCGGGUGCCGAGCAUCCGGAGUGGUGACUUUCAGGCUCCGUUCCAGACCUCUGCGGCUAUGCACCAUCCGUCUCAGGAAUC<br>UCCUACUCUGCCUGAGUCGUCGGCGACGGAUUCCGAUUAUUACUCCCCGACUGGUGGUGCGCCGCACGGGUACUGUAGUCCGACGUCGGCCUCGUACGGUA<br>AGGCCCUGAACCCGUAUCAGUACCAGUACCAUGGUGUGAACGGGUCGGCAGGGUCUUACCCUGCGAAGGCCUACGCGGACUACAGUUACGCGUCGUCGUAC<br>CACCAGUACGGGGGGGCGUAUAAUCGGGUUCCGUCGGCGACGAAUCAGCCAGAGAAGGAGGUGACUGAGCCGGAGGUGCGUAUGGUCAAUGGAAAGCCGAA<br>GAAGGUCCGCAAGCCGAGGACGAUCUAUUCGUCCUUCCAGCUUGCGGCCCUUCAGCGGCGUUUCCAGAAGACCCAGUAUCUCGCACUGCCGGAGAGGGCGG<br>AGCUGGCUGCAAGCCUGGGGCUCACUCAGACGCAGGUGAAGAUCUGGUUCCAGAACAAGCGUUCUAAGAUCAAGAAGAUCAUGAAGAACGGUGAGAUGCCG<br>CCAGAGCAUUCACCUUCGUCGAGUGACCCCAUGGCUUGCAACAGCCCGCAAUCUCCGGCAGUGUGGGAGCCGCAGGGGUCUUCGCGGAGCCUGAGUCACCA<br>CCCGCAUGCGCACCCGCCGACGAGCAACCAGUCACCAGCGAGCUCGUACCUGGAGAAUAGUGCGAGUUGGUACACUAGCGCGGCUAGUAGUAUCAACUCGC<br>ACUUGCCUCCACCGGGCUCGCUGCAGCACCCGCUGGCGCUGGCCAGCGGGACGCUGUAC
	Ours<br>-607.50 kcal/mol<br>*-602.80 kcal/mol*	AUGACUGGUGUGUUCGAUCGGCGGGUGCCGAGCAUCCGGAGUGGUGACUUUCAGGCUCCGUUUCAGACCUCUGCGGCUAUGCACCAUCCGUCUCAGGAAUC<br>UCCUACUCUGCCUGAGUCGUCGGCGACGGAUUCCGAUUAUUACUCCCCGACUGGUGGUGCGCCGCACGGGUACUGUAGUCCGACGUCGGCCUCGUAUGGUA<br>AGGCCCUGAACCCGUAUCAAUACCAGUACCAUGGUGUGAACGGGUCUGCAGGGUCUUACCCUGCGAAGGCCUACGCGGACUACAGUUACGCGUCGUCGUAC<br>CACCAGUACGGGGGGGCGUAUAAUCGGGUUCCGUCGGCGACGAAUCAGCCAGAGAAGGAGGUGACUGAGCCGGAGGUGCGUAUGGUCAAUGGAAAGCCGAA<br>GAAGGUCCGCAAGCCGAGGACGAUCUAUUCGUCCUUCCAGCUUGCGGCCCUUCAGCGGCGUUUCCAGAAGACCCAGUAUCUCGCACUGCCGGAGAGGGCGG<br>AGCUGGCUGCAAGCCUGGGGCUCACUCAGACGCAGGUGAAGAUCUGGUUCCAGAACAAGCGUUCUAAGAUCAAGAAGAUCAUGAAGAACGGUGAGAUGCCG<br>CCAGAGCAUUCACCUUCGUCGAGUGACCCCAUGGCUUGCAACAGCCCGCAAUCUCCGGCAGUGUGGGAGCCGCAGGGGUCUUCGCGGAGCCUGAGUCACCA<br>CCCGCAUGCGCACCCGCCGACGAGCAACCAGUCACCAGCGAGCUCGUAUUUGGAGAAUAGUGCGAGUUGGUACACUAGCGCGGCUAGUAGUAUCAACUCGC<br>ACUUGCCUCCACCGGGCUCGCUGCAGCACCCGCUGGCGUUGGCCAGCGGGACGCUGUAC
Q8NH87	LinearDesign<br>-572.37 kcal/mol<br>*-564.10 kcal/mol*	AUGCAGCGGAGUAACCACACGGUUACGGAGUUUAUACUGCUGGGGUUCACCACAGAUCCAGGAAUGCAGCUCGGGUUGUUCGUGGUGUUCUUGGGGGUGUA<br>CAGUCUAACUGUCGUUGGGAACAGCACGUUGAUCGUGCUGAUUUGCAACGACAGUUGUCUGCACACCCCCAUGUACUUCUUCACGGGCAACCUGAGCUUCC<br>UGGAUCUGUGGUACUCCAGCGUGUAUACUCCGAAGAUACUGGUCACAUGCAUCAGCGAGGAUAAGUCGAUAUCCUUCGCUGGAUGCUUGUGCCAGUUCUUC<br>UUCAGCGCCGGCCUUGCGUACUCCGAGUGCUACUUGUUGGCAGCAGUGGCGUAUGACAGGUACGUGGCCAUAUCAAAGCCGCUGCUGUAUGCACAGGCGAU<br>GUCAAUCAAGUUGUGCGCAUUGCUUGUAGCCGUCUCUUACUGCGGCGGCUUCAUCAACAGCAGCAUAAUCACGAAGAAGACGUUCAGUUUUAAUUUCUGCC<br>GAGAGAAUAUAAUUGACGACUUCUUCUGUGAUUUGCUGCCGUUGGUGGAGCUCGCCUGCGGUGAGAAGGGCGGCUACAAGAUAAUGAUGUACUUCUUGUUG<br>GCAUCGAAUGUGAUAUGCCCAGCGGUUUUGAUACUGGCCUCGUACCUGUUCAUCAUUACGUCAGUGCUGCGGAUAAGUAGCAGUAAGGGGUACCUCAAGGC<br>CUUCUCGACCUGUAGUAGUCACCUGACCUCGGUGACUCUGUACUACGGGUCGAUCCUUUACAUCUAUGCUCUGCCAAGGAGUAGUUACAGUUUCGACAUGG<br>AUAAGAUCGUCAGUACGUUCUAUACUGUGGUCUUUCCCAUGUUGAAUCUGAUGAUCUACUCCUUGCGGAACAAGGAUGUGAAGGAGGCGCUGAAGAAGCUG<br>CUGCCG
	Random Walk<br>-572.83 kcal/mol<br>*-563.90 kcal/mol*	AUGCAGCGGUCUAACCACACGGUUACGGAGUUUAUACUGCUGGGGUUUACCACAGAUCCAGGAAUGCAGCUCGGGUUGUUCGUGGUGUUCUUGGGGGUGUA<br>CAGUCUAACUGUCGUUGGGAACAGCACGUUGAUCGUGCUGAUUUGCAACGACAGUUGUCUGCACACCCCCAUGUACUUCUUCACGGGCAACCUGAGCUUCC<br>UGGAUCUGUGGUACUCCAGCGUGUAUACUCCGAAGAUACUGGUCACAUGCAUCAGCGAGGAUAAGUCGAUAUCCUUCGCUGGAUGCUUGUGCCAGUUCUUC<br>UUCAGCGCCGGCCUUGCGUACUCCGAGUGCUACUUGUUGGCAGCAGUGGCGUAUGACAGGUACGUGGCCAUAUCAAAGCCGCUGCUGUAUGCACAGGCGAU<br>GUCAAUCAAGUUGUGCGCAUUGCUUGUAGCCGUCUCUUACUGCGGCGGCUUCAUCAACAGCAGCAUAAUCACGAAGAAGACGUUCAGUUUUAAUUUCUGCC<br>GAGAGAAUAUAAUUGACGACUUCUUCUGUGAUUUGCUGCCGUUGGUGGAGCUCGCCUGCGGUGAGAAGGGCGGCUACAAGAUAAUGAUGUACUUCUUGUUG<br>GCAUCGAAUGUGAUAUGCCCAGCGGUUUUGAUACUGGCCUCGUACCUGUUCAUCAUUACGUCAGUGCUGCGGAUAAGUAGCAGUAAGGGGUACCUCAAGGC<br>CUUCUCGACCUGUAGUAGUCACCUGACCUCGGUGACUCUGUACUACGGGUCGAUCCUUUACAUCUAUGCUCUGCCAAGGAGUAGUUACAGUUUCGACAUGG<br>AUAAGAUCGUCAGUACGUUCUAUACUGUGGUCUUUCCCAUGUUGAAUCUGAUGAUCUACUCCUUGCGGAACAAGGAUGUGAAGGAGGCGCUGAAGAAGCUG<br>CUGCCA
	Ours<br>-573.43 kcal/mol<br>*-563.90 kcal/mol*	AUGCAGCGGUCUAACCACACGGUUACGGAGUUUAUACUGCUGGGGUUUACCACAGAUCCAGGAAUGCAGCUCGGGUUGUUCGUGGUGUUCUUGGGGGUGUA<br>CAGUCUAACUGUCGUUGGGAACAGCACGUUGAUCGUGCUGAUUUGCAACGACAGUUGUCUGCACACCCCCAUGUACUUCUUCACGGGCAACCUGAGCUUCC<br>UGGAUCUGUGGUACUCCAGCGUGUAUACUCCGAAGAUACUGGUCACAUGCAUCAGCGAGGAUAAGAGCAUAUCCUUCGCUGGAUGCUUGUGCCAGUUCUUC<br>UUCAGCGCCGGCCUUGCGUACUCCGAGUGCUACUUAUUGGCAGCAGUGGCGUAUGACAGGUACGUGGCCAUAUCAAAGCCGCUGUUGUAUGCACAGGCGAU<br>GUCAAUCAAGUUGUGCGCAUUGCUUGUAGCCGUCUCUUACUGCGGCGGCUUCAUCAACAGCAGCAUAAUCACGAAGAAGACGUUCAGUUUUAAUUUCUGUA<br>GAGAGAAUAUAAUUGACGACUUCUUCUGUGAUUUGCUGCCGUUGGUGGAGCUCGCCUGCGGUGAGAAGGGCGGCUACAAGAUAAUGAUGUACUUCUUGUUG<br>GCAUCGAAUGUGAUAUGCCCAGCGGUUUUGAUAUUGGCCUCGUACCUGUUCAUCAUUACGUCAGUGCUGCGGAUAAGUAGCAGUAAGGGGUACCUCAAGGC<br>CUUUUCGACCUGUAGUAGUCACCUGACGUCGGUGACUCUCUACUACGGGUCGAUCCUUUACAUCUAUGCUCUGCCAAGGAGUAGUUACAGUUUCGACAUGG<br>AUAAGAUCGUCAGUACGUUCUAUACUGUGGUCUUUCCCAUGUUGAAUCUGAUGAUCUACUCCUUGCGGAACAAGGAUGUGAAGGAGGCGCUGAAGAAGCUG<br>CUGCCU

Continued on next page

Table S5 – continued from previous page

ID	Method	mRNA Design
Q8NGU1	LinearDesign<br>-612.55 kcal/mol<br>*-606.30 kcal/mol*	AUGUUGGGCAACUAUAGCAGUGCUACUGAGUUCUUUCUGCUUGGGUUUCCGGGCAGUCAAGAGGUCUGCCGUAUCCUGUUCGCCACGUUCUUUCUGCUGUA<br>CGCCGUGACGGUGAUGGGGAACGUCGUCAUCAUCAUCACGGUGUGCGUAGACAAGUGCCUGCAAUCGCCAAUUUACUUCUUUCUGGGCCAUCUCUGCGUCC<br>UCGAGAUUCUGAUAACAAGUACAGCUGUGCCGUUCAUGCUGUGGGGCUUAUUGCUGCCGAGCACGCAGAUAAUGAGCCUCACAGCAUGUGCGGCACAGCUG<br>UACUUGUAUCUGAGUCUCGGGACGCUAGAGUUGGCCCUGAUGGGAGUGAUGGCGGUUGACAGGUACGUAGCGGUGUGUAACCCACUCCGCUACAACAUUAU<br>CAUGAAUUCGUCGACGUUUAUAUGGGUGAUAAUUGUGUCGUGGGUGUUAGGCUUCCUGAGUGAGAUAUGGCCAGUGUAUGCCACCUUUCAGCUCACCUUCU<br>GCAAGAGCAGCGUCCUCGAUCACUUCUACUGUGAUCGAGGACAGCUGCUGAAGGUGAGCUGUGAGGAUACACUGUUCAGGGAGUUCAUCCUCUUCCUGAUG<br>GCCGUGUUCAUCAUUAUAGGAAGCCUGAUACCCACGAUAGUCAGUUACACCUAUAUAAUCUCGACGAAUCUCAAGAUACCGAGUGCAAGUGGCUGGCGCAA<br>GUCGUUCUCGACUUGCGCCAGCCACUUCACUUACGUGGUGAUAGGAUACGGCAGCUGCCUCUUUCUGUACGUGAAGCCCAAGCAGACUCAGGCCGCCGAGU<br>ACAACAGGGUGGUGUCCCUGUUGGUCUUGGUGGUCACGCCGUUCCUGAACCCUUUUAUCUUUACGCUGCGUAAUGAUAAAUUCAUCCAGGCGUUCGGGGAC<br>GGCAUGAAGCACUGCUAUAAGUUGCUCAAGAAC
	Random Walk<br>-612.67 kcal/mol<br>*-606.30 kcal/mol*	AUGUUGGGCAACUAUAGCAGUGCUACUGAGUUCUUUCUGCUUGGGUUUCCGGGCAGUCAAGAGGUCUGCCGUAUCCUGUUCGCCACGUUUUUUCUGCUGUA<br>CGCCGUGACGGUGAUGGGGAACGUCGUCAUCAUCAUCACGGUGUGCGUAGACAAGUGCCUGCAAUCGCCAAUUUACUUCUUUCUGGGCCAUCUCUGCGUCC<br>UCGAGAUUCUGAUAACAAGUACAGCUGUGCCGUUCAUGCUGUGGGGCUUAUUGCUGCCGAGCACGCAGAUAAUGAGCCUCACAGCAUGUGCGGCACAGCUG<br>UACUUGUAUCUGAGUCUCGGGACGCUAGAGUUGGCCCUGAUGGGAGUGAUGGCGGUUGACAGGUACGUAGCGGUGUGUAACCCACUCCGCUACAACAUUAU<br>CAUGAAUUCGUCGACGUUUAUAUGGGUGAUAAUUGUGUCGUGGGUAUUAGGCUUCCUGAGUGAGAUAUGGCCAGUGUAUGCUACCUUUCAGCUCACCUUCU<br>GCAAGAGCAGCGUCCUCGAUCACUUCUACUGUGAUCGAGGACAGCUGCUGAAGGUGAGCUGUGAGGAUACACUGUUCAGGGAGUUCAUCCUCUUCCUGAUG<br>GCCGUGUUCAUCAUUAUAGGAAGCCUGAUACCCACGAUAGUCAGUUACACCUAUAUAAUCUCGACGAAUCUCAAGAUACCGAGUGCAAGUGGCUGGCGCAA<br>GUCGUUCUCGACUUGCGCCAGCCACUUCACUUACGUGGUGAUAGGAUACGGCAGCUGCCUCUUUCUGUACGUGAAGCCCAAGCAGACUCAGGCCGCCGAGU<br>ACAACAGGGUGGUGUCCCUGUUGGUCUUGGUGGUCACGCCGUUCCUGAACCCUUUUAUCUUUACGCUGCGUAAUGAUAAAUUUAUCCAGGCGUUCGGGGAC<br>GGCAUGAAGCACUGCUAUAAGUUGCUCAAGAAC
	Ours<br>-613.42 kcal/mol<br>*-605.50 kcal/mol*	AUGUUGGGCAACUAUAGCAGUGCUACUGAGUUCUUUCUGCUUGGGUUUCCGGGCAGUCAAGAGGUCUGCCGUAUCCUGUUCGCCACGUUUUUUCUACUGUA<br>CGCCGUGACGGUGAUGGGAAUGUCGUCAUCAUCAUCACGGUGUGCGUAGAUAAGUGCCUGCAAUCGCCAAUUUACUUCUUUCUGGGCCAUCUCUGCGUCC<br>UCGAGAUUCUGAUUACAAGUACAGCUGUGCCGUUCAUGCUGUGGGGCUUAUUGCUGCCGAGCACGCAGAUAAUGAGCCUCACAGCAUGUGCGGCACAGCUG<br>UACUUGUAUCUGAGUCUCGGGACGCUAGAGUUGGCCCUGAUGGGAGUGAUGGCGGUUGACAGGUACGUAGCGGUGUGUAAUCCACUCCGCUACAAUAUCAU<br>CAUGAAUUCGUCGACGUUUAUAUGGGUGAUAAUUGUGUCGUGGGUGUUAGGCUUCCUGAGUGAGAUAUGGCCAGUGUAUGCCACCUUUCAGCUCACCUUCU<br>GCAAGAGCUCUGUCCUCGAUCACUUCUACUGUGAUCGAGGACAGCUCUUGAAGGUGAGCUGUGAGGAUACACUGUUCAGGGAGUUUAUCCUCUUCCUGAUG<br>GCCGUGUUCAUCAUUAUAGGAAGCCUGAUACCCACGAUAGUCAGUUACACCUAUAUAAUCUCGACGAAUUUGAAGAUACCGAGUGCAAGUGGCUGGCGCAA<br>GUCGUUUUCGACUUGCGCCAGCCACUUCACUUACGUGGUGAUAGGAUACGGCAGCUGCCUCUUUCUGUACGUGAAGCCCAAGCAGACUCAGGCCGCCGAGU<br>ACAACAGGGUGGUGUCCCUGUUGGUCUUGGUGGUCACGCCGUUCCUGAACCCUUUUAUCUUUACGCUGCGUAAUGAUAAAUUUAUCCAGGCGUUCGGGGAC<br>GGCAUGAAGCACUGCUAUAAGUUGCUCAAGAAU
Q8NGC9	LinearDesign<br>-582.39 kcal/mol<br>*-576.40 kcal/mol*	AUGUCAUUUUUCUUCGUAGAUCUUCGCCCCAUGAACAGGAGCGCGACGCACAUAGUCACCGAGUUCAUACUGCUGGGUUUUCCAGGGUGCUGGAAGAUCCA<br>GAUCUUCCUGUUCAGUUUGUUCUUGGUGAUCUAUGUGCUCACGCUCCUGGGCAAUGGGGCGAUAAUCUACGCGGUGAGAUGCAACCCGCUGUUGCAUACGC<br>CUAUGUACUUCUUGCUCGGGAAUUUCGCCUUCUUGGAGAUAUGGUACGUGAGCAGUACCAUACCCAACAUGUUGGUGAAUAUCCUGAGCAAGACAAAGGCG<br>AUAAGCUUCAGCGGGUGCUUCCUGCAGUUUUACUUCUUCUUUUCGCUGGGUACUACGGAGUGCUUGUUCUUGGCCGUGAUGGCGUACGAUCGCUAUCUGGC<br>CAUCUGUCAUCCACUCCAGUACCCAGCGAUCAUGACGGUGAGAUUCUGCGGGAAGCUAGUCAGCUUCUGCUGGCUAAUCGGAUUUCUGGGGUAUCCCAUCC<br>CGAUAUUUUACAUUUCGCAGCUGCCAUUCUGCGGGCCCAAUAUCAUAGAUCACUUCCUCUGUGAUAUGGACCCGCUGAUGGCACUGUCGUGCGCUCCGGCG<br>CCGAUAACUGAGUGUAUCUUUUACACUCAGUCCAGCUUGGUGCUCUUUUUUACCUCAAUGUAUAUAUUAAGGAGUUAUAUACUGCUGCUGACGGCAGUGUU<br>CCAAGUGCCGUCAGCAGCGGGGCGUCGUAAGGCUUUUAGCACGUGUGGGAGUCAUCUGGUAGUAGUGUCGCUGUUCUAUGGGACGGUGAUGGUAAUGUACG<br>UAUCACCGACCUAUGGAAUACCGACACUGCUCCAGAAGAUUCUCACACUUGUUUAUAGCGUUACGACGCCCCUGUUUAACCCCUUGAUAUAUACAUUGAGG<br>AAUAAAGACAUGAAGCUGGCGCUGCGAAAUGUAUUAUUCGGGAUGCGGAUACGCCAGAAUUCC
	Random Walk<br>-582.86 kcal/mol<br>*-576.40 kcal/mol*	AUGUCAUUUUUCUUCGUAGAUCUUCGCCCCAUGAACAGGAGCGCGACGCACAUAGUCACCGAGUUCAUACUGCUGGGUUUUCCAGGGUGCUGGAAGAUCCA<br>GAUCUUCCUGUUCAGUUUGUUCUUGGUGAUCUAUGUGCUCACGCUCCUGGGCAAUGGGGCGAUAAUCUACGCGGUGAGAUGCAACCCGCUGUUGCAUACGC<br>CUAUGUACUUCUUGCUCGGGAAUUUCGCCUUCUUGGAGAUAUGGUACGUAAGCAGUACCAUACCCAACAUGUUGGUGAAUAUCCUGAGCAAGACAAAGGCG<br>AUAAGCUUCAGCGGGUGCUUCCUGCAGUUUUACUUUUUCUUUUCGCUGGGUACUACGGAGUGCUUGUUCUUGGCCGUGAUGGCGUAUGAUCGCUAUCUGGC<br>CAUCUGUCAUCCACUCCAGUACCCAGCGAUAAUGACGGUGAGAUUCUGCGGGAAGCUAGUCAGCUUCUGCUGGCUAAUCGGAUUUCUGGGGUAUCCCAUCC<br>CGAUAUUUUACAUUUCGCAGCUGCCAUUCUGCGGGCCCAAUAUCAUAGAUCAUUUCCUCUGUGAUAUGGACCCGCUGAUGGCACUGUCGUGCGCUCCGGCG<br>CCGAUAACUGAGUGUAUAUUUUACACUCAGUCCAGCUUGGUGCUCUUUUUUACCUCAAUGUAUAUAUUAAGGAGUUAUAUACUGCUGCUGACGGCAGUGUU<br>CCAAGUGCCGUCAGCAGCGGGGCGUCGUAAGGCUUUUAGCACGUGUGGGAGUCAUCUGGUAGUAGUGUCGCUGUUCUAUGGGACGGUGAUGGUAAUGUACG<br>UAUCACCGACCUAUGGAAUACCGACACUGCUCCAGAAGAUUCUCACACUUGUUUAUAGCGUUACGACGCCCCUGUUUAACCCCUUGAUAUAUACAUUGAGG<br>AAUAAAGACAUGAAGCUGGCGCUGCGAAAUGUAUUAUUCGGGAUGCGGAUACGCCAGAAUUCC
	Ours<br>-583.43 kcal/mol<br>*-576.20 kcal/mol*	AUGUCAUUUUUCUUCGUAGAUCUUCGCCCCAUGAACAGGAGCGCGACGCACAUAGUCACCGAGUUCAUACUGCUGGGUUUUCCAGGGUGCUGGAAGAUCCA<br>GAUCUUCUUAUUCAGUUUGUUCUUGGUGAUCUAUGUGCUCACGCUCCUGGGCAAUGGGGCGAUAAUCUACGCGGUGAGAUGCAACCCGCUGUUGCAUACGC<br>CUAUGUACUUCUUGCUCGGGAAUUUCGCCUUCUUGGAGAUAUGGUACGUGUCGAGUACCAUACCCAACAUGUUGGUGAAUAUCCUGAGCAAGACAAAGGCG<br>AUAAGCUUCAGCGGGUGCUUCCUGCAGUUUUACUUCUUCUUUUCGCUGGGUACUACGGAGUGUUUGUUUUUGGCCGUGAUGGCGUACGAUCGCUAUCUGGC<br>CAUAUGUCAUCCACUCCAGUACCCAGCGAUAAUGACGGUGAGAUUCUGCGGGAAGCUAGUCAGCUUCUGCUGGCUAAUCGGGUUUCUGGGGUAUCCCAUCC<br>CGAUAUUUUACAUUUCGCAGCUGCCAUUCUGCGGGCCCAAUAUCAUAGAUCACUUUCUCUGUGAUAUGGACCCGCUGAUGGCAUUGUCGUGCGCUCCGGCG<br>CCGAUAACUGAGUGUAUCUUUUACACUCAGUCCAGCUUGGUGCUCUUUUUUACCUCAAUGUAUAUAUUAAGGAGUUAUAUACUGCUGCUGACGGCAGUGUU<br>UCAAGUGCCGUCAGCAGCGGGGCGUCGUAAGGCUUUUAGCACGUGUGGGAGUCAUCUGGUAGUAGUGUCGUUAUUCUAUGGGACGGUGAUGGUAAUGUACG<br>UAUCACCGACCUAUGGAAUACCGACACUGCUCCAGAAGAUUCUCACACUUGUUUAUAGCGUUACGACGCCCCUGUUUAACCCUUAAUAUAUACAUUGAGG<br>AAUAAAGACAUGAAGCUGGCGCUGCGAAAUGUAUUAUUCGGGAUGCGGAUACGCCAGAACUCG

Continued on next page

Table S5 – continued from previous page

ID	Method	mRNA Design
Q99729	LinearDesign<br>-667.21 kcal/mol<br>*-663.00 kcal/mol*	AUGAGUGAGGCUGGUGAGGAGCAGCCCAUGGAGACCACCGGGGCGACUGAGAACGGCCAUGAGGCGGUCCCUGAGGCUAGUCGUGGCCGGGGCUGGACUGG<br>GGCCGCGGCUGGGGCUGGCGGCGCCACCGCCGCCCCCCCUCCGGAAAUCAGAAUGGGGCCGAGGGCGACCAGAUCAACGCCUCGAAGAAUGAAGAGGAUG<br>CAGGCAAGAUGUUCGUGGGCGGCCUGAGUUGGGACACAUCUAAGAAGGACUUGAAGGACUACUUCACCAAGUUUGGUGAAGUAGUCGACUGUACCAUCAAG<br>AUGGAUCCCAACACAGGCCGCUCACGAGGGUUUGGCUUCAUCCUCUUCAAGGAUGCCGCGUCCGUCGAGAAGGUGCUCGACCAGAAGGAGCACCGUCUCGA<br>CGGACGCGUCAUCGACCCCAAAAAGGCCAUGGCCAUGAAGAAGGAUCCCGUAAAGAAGAUCUUCGUUGGGGGACUCAACCCUGAGUCCCCCACCGAAGAGA<br>AGAUACGGGAGUACUUCGGCGAGUUUGGGGAGAUCGAGGCGAUCGAGUUGCCCAUGGACCCCAAGCUGAACAAGCGGCGGGGCUUCGUCUUCAUCACCUUC<br>AAGGAGGAGGAGCCCGUCAAGAAGGUGUUGGAGAAGAAGUUCCACACCGUCUCCGGUUCCAAGUGCGAGAUCAAGGUUGCCCAGCCCAAGGAGGUGUACCA<br>GCAGCAGCAGUACGGCUCCGGUGGGCGGGGCAACCGGAAUCGCGGGAACCGGGGUUCCGGAGGGGGGGGCGGCGGUGGCGGCCAGUCCCAGUCGUGGAACC<br>AGGGCUAUGGUAACUAUUGGAACCAGGGCUAUGGCUACCAGCAGGGCUAUGGCCCUGGUUACGGGGGGUAUGAUUACUCCCCGUAUGGUUACUAUGGCUAC<br>GGCCCCGGCUACGACUACUCUCAGGGAUCGACCAAUUAUGGCAAGUCUCAGCGCCGCGGUGGUCACCAGAACAAUUACAAGCCUUAC
	Random Walk<br>-667.74 kcal/mol<br>*-663.00 kcal/mol*	AUGAGUGAGGCUGGUGAGGAGCAGCCCAUGGAGACCACCGGGGCGACUGAGAACGGCCAUGAGGCGGUCCCUGAGGCUAGUCGUGGCCGGGGCUGGACUGG<br>GGCCGCGGCUGGGGCUGGCGGCGCCACCGCCGCCCCCCCUCCGGAAAUCAGAAUGGGGCCGAGGGCGACCAGAUCAACGCCUCGAAGAAUGAAGAGGAUG<br>CAGGCAAGAUGUUCGUGGGCGGCCUGAGUUGGGAUACAUCUAAGAAGGACUUGAAGGACUACUUCACCAAGUUUGGUGAAGUAGUCGACUGUACCAUCAAG<br>AUGGAUCCCAACACAGGCCGCUCACGAGGGUUUGGCUUCAUCCUCUUCAAGGAUGCCGCGUCCGUCGAGAAGGUGCUCGACCAGAAGGAGCACCGUCUCGA<br>CGGACGCGUCAUCGACCCCAAAAAGGCCAUGGCCAUGAAGAAGGAUCCCGUAAAGAAGAUCUUCGUUGGGGGACUCAACCCUGAGUCCCCCACCGAAGAGA<br>AGAUACGGGAGUACUUCGGCGAGUUUGGGGAGAUCGAGGCGAUCGAGUUGCCCAUGGACCCCAAGCUGAACAAGCGGCGGGGCUUCGUCUUCAUCACCUUC<br>AAGGAGGAGGAGCCCGUCAAGAAGGUGUUGGAGAAGAAGUUCCACACCGUCUCCGGUUCCAAGUGCGAGAUCAAGGUUGCCCAGCCCAAGGAGGUGUACCA<br>GCAGCAGCAGUACGGCUCCGGUGGGCGGGGCAACCGGAAUCGCGGGAACCGGGGUUCCGGAGGGGGGGGCGGCGGUGGCGGCCAGUCCCAGUCGUGGAACC<br>AGGGCUAUGGUAACUAUUGGAACCAGGGCUAUGGCUACCAGCAGGGCUAUGGCCCUGGUUACGGGGGGUAUGAUUACUCCCCGUAUGGUUACUAUGGCUAC<br>GGCCCCGGCUACGACUACUCUCAGGGAUCGACCAAUUAUGGCAAGUCUCAGCGCCGCGGUGGUCACCAGAAUAAUUACAAGCCUUAC
	Ours<br>-668.21 kcal/mol<br>*-663.00 kcal/mol*	AUGAGUGAGGCUGGUGAGGAGCAGCCCAUGGAGACCACCGGGGCGACUGAGAACGGCCAUGAGGCGGUCCCUGAGGCUAGUCGUGGCCGGGGCUGGACUGG<br>GGCCGCGGCUGGGGCUGGCGGCGCCACCGCCGCCCCCCCUCCGGAAAUCAGAAUGGGGCCGAGGGCGACCAGAUCAACGCCUCGAAGAAUGAAGAGGAUG<br>CAGGCAAGAUGUUCGUGGGCGGCCUGAGUUGGGAUACAUCUAAGAAGGACUUAAAGGACUACUUCACCAAGUUUGGUGAAGUAGUCGAUUGUACCAUCAAG<br>AUGGAUCCCAACACAGGCCGCUCACGAGGGUUUGGCUUCAUCCUCUUCAAGGAUGCCGCGUCCGUCGAGAAGGUGCUCGAUCAGAAGGAGCACCGUCUCGA<br>CGGACGCGUCAUCGACCCCAAAAAAGCCAUGGCCAUGAAGAAGGAUCCCGUAAAGAAGAUCUUCGUUGGGGGACUCAAUCCUGAGUCCCCCACCGAAGAGA<br>AGAUACGGGAGUACUUCGGCGAGUUUGGGGAGAUCGAGGCGAUCGAGUUGCCCAUGGACCCCAAGCUGAAUAAGCGGAGGGGCUUCGUCUUCAUCACCUUC<br>AAGGAGGAGGAGCCCGUCAAGAAGGUGUUGGAGAAGAAGUUCCAUACCGUCUCCGGUUCCAAGUGCGAGAUCAAGGUUGCCCAGCCCAAGGAGGUGUACCA<br>GCAGCAGCAGUACGGCUCCGGUGGGCGGGGCAACCGGAAUCGCGGGAACCGGGGUUCCGGAGGGGGGGGCGGCGGUGGCGGCCAGUCCCAGUCGUGGAACC<br>AGGGCUAUGGUAACUAUUGGAACCAGGGCUAUGGCUACCAGCAGGGCUAUGGCCCUGGUUACGGGGGGUAUGAUUACUCCCCGUAUGGUUACUAUGGCUAC<br>GGCCCCGGCUACGACUACUCUCAGGGAUCGACCAAUUAUGGCAAGUCUCAGCGCCGCGGUGGUCACCAGAAUAAUUACAAGCCUUAC
Q9P2M1	LinearDesign<br>-663.54 kcal/mol<br>*-656.10 kcal/mol*	AUGAAGCUGACCUCGGAGAAGUUGCCAAAGAACCCCUUCUACGCCUCUGUCAGCCAGUACGCGGCGAAGAAUCAGAAGUUCUUUCAGUGGAAGAAAGAAAA<br>GACUGAUUACACGCACGCGAAUCUGGUUGACAAGGCGUUGCAGCUCCUGAAGGAGCGCAUCCUGAAGGGGGACACUUUGGCAUACUUUCUCCGAGGUCAGC<br>UUUAUUUCGAGGAGGGUUGGUACGAGGAGGCCUUGGAGCAGUUCGAGGAAAUCAAAGAGAAGGACCACCAAGCUACGUACCAGCUUGGGGUCAUGUACUAU<br>GAUGGCCUCGGGACGACUUUAGAUGCCGAGAAGGGCGUGGACUAUAUGAAGAAGAUCUUGGAUAGUCCAUGCCCGAAGGCUCGGCAUCUGAAGUUUGCCGC<br>GGCCUAUAAUCUCGGGCGGGCAUACUACGAGGGCAAGGGCGUCAAGAGGAGUAACGAGGAGGCCGAGCGCCUCUGGCUCAUUGCCGCUGACAACGGGAAUC<br>CGAAGGCCAGUGUGAAGGCCCAGAGCAUGCUUGGGCUCUAUUACAGUACUAAAGAGCCCAAGGAGCUCGAGAAGGCCUUCUACUGGCAUUCGGAGGCCUGU<br>GGCAACGGCAAUCUGGAGAGCCAAGGGGCGCUCGGCCUCAUGUACCUCUAUGGUCAGGGGAUCCGCCAGGACACGGAGGCCGCGCUCCAGUGCCUCCGGGA<br>GGCAGCGGAGCGCGGCAACGUGUACGCGCAAGGCAACUUGGUGGAGUACUACUACAAGAUGAAGUUCUUCACCAAGUGCGUUGCGUUCUCCAAGCGGAUCG<br>CCGACUAUGACGAGGUGCACGAUAUUCCUAUGAUCGCCCAGGUCACCGAUUGCCUGCCCGAGUUUAUAGGCCGCGGCAUGGCUAUGGCCUCGUUUUAUCAC<br>GCGCGUUGUCUCCAGCUGGGGCUGGGGAUAACGCGCGAUGAGACGACGGCCAAGCACUACUACUCCAAGGCCUGCCGUCUCAACCCUGCGCUGGCGGAUGA<br>GCUGCACUCGCUGCUCAUCCGCCAGCGCAUC
	Random Walk<br>-664.15 kcal/mol<br>*-656.10 kcal/mol*	AUGAAGCUGACCUCGGAGAAGUUGCCAAAGAACCCCUUCUACGCCUCUGUCAGCCAGUACGCGGCGAAGAAUCAGAAGUUCUUUCAGUGGAAGAAAGAAAA<br>GACUGAUUACACGCACGCGAAUCUGGUUGACAAGGCGUUGCAGCUCCUGAAGGAGCGCAUCCUGAAGGGGGACACUUUGGCAUACUUUCUCCGAGGUCAGC<br>UUUAUUUCGAGGAGGGUUGGUACGAGGAGGCCUUGGAGCAGUUCGAGGAAAUCAAAGAGAAGGACCACCAAGCUACGUACCAGCUUGGGGUCAUGUACUAU<br>GAUGGCCUCGGGACGACUUUAGAUGCCGAGAAGGGCGUGGACUAUAUGAAGAAGAUCUUGGAUAGUCCAUGCCCGAAGGCUCGGCAUCUGAAGUUUGCCGC<br>GGCCUAUAAUCUCGGGCGGGCAUACUACGAGGGCAAGGGCGUCAAGAGGAGUAACGAGGAGGCCGAGCGCCUCUGGCUCAUUGCCGCUGACAACGGGAAUC<br>CGAAGGCCAGUGUGAAGGCCCAGAGCAUGCUUGGGCUCUAUUACAGUACUAAAGAGCCCAAGGAGCUCGAGAAGGCCUUCUACUGGCAUUCGGAGGCCUGU<br>GGCAACGGCAAUCUGGAGAGCCAGGGGGCGCUCGGCCUCAUGUACCUCUAUGGUCAGGGGAUCCGCCAGGACACGGAGGCCGCGCUCCAGUGCCUCCGGGA<br>GGCAGCGGAGCGCGGCAACGUGUACGCGCAAGGCAACUUGGUGGAGUACUACUACAAGAUGAAGUUCUUCACCAAGUGCGUUGCGUUCUCCAAGCGGAUCG<br>CCGACUAUGACGAGGUGCACGAUAUUCCUAUGAUCGCCCAGGUCACCGAUUGCCUGCCCGAGUUUAUAGGCCGCGGCAUGGCUAUGGCCUCGUUUUAUCAC<br>GCGCGUUGUCUCCAGCUUGGGCUGGGGAUAACGCGCGAUGAGACGACGGCCAAGCACUACUACUCCAAGGCCUGCCGUCUCAACCCUGCGCUGGCGGAUGA<br>GCUGCAUUCGCUGCUCAUCCGCCAGCGCAUC
	Ours<br>-665.07 kcal/mol<br>*-655.10 kcal/mol*	AUGAAGCUGACCUCGGAGAAGUUGCCAAAGAACCCCUUCUACGCCUCUGUCAGCCAGUACGCGGCGAAGAAUCAGAAGUUCUUUCAGUGGAAGAAAGAAAA<br>GACUGAUUAUACGCACGCGAACCUGGUUGACAAGGCGUUGCAGCUCCUGAAGGAGCGCAUAUUGAAGGGGGAUACUUUGGCAUACUUUCUCCGAGGUCAGC<br>UUUAUUUCGAGGAGGGUUGGUACGAGGAGGCCUUGGAGCAGUUCGAGGAAAUCAAGGAGAAGGACCACCAAGCUACGUAUCAGCUUGGGGUCAUGUACUAU<br>GAUGGCCUCGGAACGACUUUAGAUGCCGAGAAGGGCGUGGACUAUAUGAAGAAGAUCUUGGAUAGUCCAUGCCCGAAGGCUCGGCAUCUGAAGUUUGCCGC<br>GGCCUAUAAUCUCGGGCGGGCAUAUUACGAGGGCAAGGGCGUCAAGCGGAGUAACGAGGAGGCCGAGCGCCUCUGGCUCAUUGCCGCUGACAACGGGAAUC<br>CGAAGGCCAGUGUGAAGGCCCAGAGCAUGCUUGGGCUCUAUUACAGUACUAAAGAGCCCAAGGAGCUCGAGAAGGCCUUCUACUGGCAUUCGGAGGCCUGU<br>GGCAACGGCAAUCUGGAGAGCCAGGGGGCGCUCGGCCUCAUGUACCUCUAUGGUCAGGGGAUCCGCCAGGACACGGAGGCCGCGCUCCAGUGCCUCAGAGA<br>GGCAGCGGAGCGCGGCAACGUGUAUGCGCAAGGCAACUUGGUGGAGUACUACUAUAAGAUGAAGUUCUUCACCAAGUGCGUUGCGUUUUCCAAGCGGAUCG<br>CCGACUAUGAUGAGGUGCAUGAUAUUCCGAUGAUCGCCCAGGUCACCGAUUGCCUGCCCGAGUUUAUAGGCCGCGGCAUGGCUAUGGCCUCGUUUUAUCAC<br>GCGCGUUGUCUCCAGCUGGGGCUGGGGAUAACGCGCGAUGAGACGACGGCCAAGCACUACUACUCCAAGGCCUGCCGUCUCAACCCUGCGCUGGCGGAUGA<br>GCUGCAUUCGCUGCUCAUCCGCCAGCGCAUC

Continued on next page

Table S5 – continued from previous page

ID	Method	mRNA Design
	LinearDesign<br>-2511.49 kcal/mol<br>*-2486.70 kcal/mol*	AUGUUCGUGUUUCUUGUCCUCCUUCCACUGGUUUCGAGUCAGUGCGUCAAUCUUACAACACGAACCCAGCUGCCGCCAGCCUACACGAACUCCUUCACGCGGGGAGUGUACUACCCCGACAAGGUGUUCCGCUCGUCUGUU<br>CUGCACAGCACGCAGGACCUCUUCCUCCCGUUCUUCUCGAACGUGACAUGGUUCCAUGCCAUUCACGUUUCGGGAACGAACGGGACGAAGAGGUUCGACAACCCUGUUCUGCCGUUCAACGACGGGGUGUACUUCGCUUCG<br>ACAGAGAAGUCCAACAUUAUUCGCGGAUGGAUAUUCGGAACCACUCUCGAUUCCAAGACUCAGUCCUUGUUGAUAGUGAACAACGCCACGAACGUGGUCAUUAAGGUCUGUGAGUUUCAGUUCUGUAAUGACCCGUUCCUG<br>GGUGUUUACUAUCACAAGAACAACAAGUCUUGGAUGGAGAGUGAGUUCCGAGUGUAUUCAUCCGCGAAUAACUGUACCUUCGAGUAUGUCAGUCAGCCGUUCCUGAUGGAUCUUGAAGGCAAGCAGGGCAAUUUCAAGAAU<br>UUGCGCGAGUUUGUCUUCAAGAACAUCGACGGCUACUUCAAGAUAUACUCGAAGCACACGCCAAUCAACCUCGUCCGUGAUCUCCCGCAGGGCUUCAGCGCUCUGGAGCCGCUGGUGGACUUGCCGAUAGGCAUCAACAUC<br>ACGCGGUUCCAGACGCUGCUGGCCCUGCACAGGAGUUACCUGACGCCAGGUGACUCCUCGUCCGGGUGGACUGCAGGUGCCGCCGCGUACUACGUGGGGUACCUGCAGCCCCGGACGUUCUUGUUGAAGUACAACGAGAAC<br>GGGACGAUCACGGACGCGGUUGAUUGCGCGUUGGACCCUCUGUCGGAGACGAAGUGCACCCUGAAGUCGUUUACGGUAGAAAAGGGGAUCUAUCAGACCUCCAACUUCCGCGUCCAGCCGACUGAGAGUAUCGUUCGGUUC<br>CCGAACAUCACGAACCUGUGUCCGUUCGGAGAGGUCUUCAACGCGACCAGGUUCGCCUCCGUGUACGCUUGGAACAGGAAGAGGAUCUCGAAUUGUGUAGCAGACUACAGUGUGCUAUACAAUUCGGCGUCCUUUUCCACU<br>UUCAAGUGUUACGGAGUGUCGCCCACGAAGUUGAACGACCUCUGCUUCACCAACGUGUAUGCGGAUUCCUUCGUCAUCCGUGGUGACGAAGUGCGUCAGAUUGCUCCGGGACAGACGGGUAAGAUAGCGGACUACAAUUAU<br>AAGUUGCCCGACGACUUUACUGGCUGCGUUAUUGCUUGGAAUAGCAAUAACUUAGACAGUAAAGUCGGGGGCAACUAUAACUAUCUUUACCGUCUGUUCCGGAAGAGCAAUCUGAAGCCCUUCGAGCGCGAUAUCUCGACC<br>GAAAUCUACCAGGCAGGCUCCACCCCGUGCAACGGGGUGGAGGGCUUCAAUUGUUACUUUCCGUUGCAGAGCUACGGAUUUCAGCCCACCAACGGGGUAGGGUACCAGCCCUACCGCGUGGUGGUGCUGAGUUUCGAGCUU<br>CUGCACGCCCCCGCGACUGUCUGUGGGCCCAAGAAGUCGACGAACUUGGUGAAGAACAAGUGCGUCAACUUCAACUUCAAUGGGCUCACAGGCACGGGGGUGUUGACGGAGAGUAACAAGAAGUUCCUGCCUUUCCAGCAG<br>UUCGGUCGAGAUAUCGCGGACACCACGGAUGCCGUAAGGGAUCCGCAGACGCUUGAGAUUCUGGACAUCACGCCCUGCAGCUUCGGGGGCGUCAGUGUAAUCACGCCUGGUACAAACACCAGCAACCAGGUUGCGGUGUUG<br>UACCAGGACGUGAAUUGCACUGAGGUCCCCGUAGCGAUCCACGCGGAUCAGCUAACCCCAACGUGGAGGGUGUACUCGACAGGGAGUAAUGUCUUCCAGACUCGCGCUGGUUGUCUGAUUGGCGCUGAGCAUGUGAACAAC<br>UCGUACGAGUGUGACAUCCCCAUUGGAGCGGGGAUCUGCGCGUCGUACCAGACCCAGACGAACAGCCCGAGGCGUGCCCGCUCAGUAGCGUCGCAGUCGAUCAUCGCGUACACGAUGAGCUUGGGGGCGGAGAACAGUGUA<br>GCCUAUUCGAACAACAGCAUUGCUAUCCCCACAAAUUUUACAAUUAGUGUAACCACCGAGAUCUUGCCGGUCUCGAUGACCAAGACCUCGGUGGAUUGCACUAUGUAUAUUUGUGGGGAUAGCACUGAGUGUUCUAACCUC<br>CUGCUCCAGUACGGCAGUUUCUGUACGCAGCUCAACCGAGCGCUUACAGGAAUUGCCGUGGAGCAGGACAAGAACACUCAGGAGGUGUUUGCGCAGGUCAAGCAGAUCUACAAGACACCUCCGAUCAAGGACUUUGGCGGC<br>UUUAACUUCUCCCAGAUACUCCCCGACCCCAGCAAGCCCAGCAAGCGUAGCUUUAUUGAAGAUCUGCUCUUCAAUAAAGUUACGCUUGCUGAUGCUGGGUUCAUCAAGCAGUACGGGGACUGUCUGGGAGAUAUAGCCGCC<br>AGAGACUUGAUCUGUGCUCAGAAGUUCAAUGGGCUCACUGUUCUCCCCCCCUUGCUCACGGACGAGAUGAUCGCGCAGUACACUUCGGCGCUGCUGGCGGGCACGAUCACCUCGGGCUGGACGUUUGGGGCUGGUGCGGCG<br>CUGCAGAUCCCGUUCGCCAUGCAGAUGGCGUACCGCUUCAAUGGGAUCGGUGUCACACAGAACGUGUUGUACGAGAAUCAGAAGCUCAUCGCCAAUCAGUUCAACAGCGCGAUCGGGAAGAUACAGGACUCCCUGUCGAGU<br>ACAGCCUCCGCGUUGGGGAAGCUGCAGGACGUGGUGAACCAGAAUGCUCAAGCGUUGAACACGUUGGUGAAGCAGUUGUCGUCCAACUUCGGGGCGAUAAGUUCGGUGCUGAACGAUAUUCUCAGUCGGCUGGACAAGGUG<br>GAAGCGGAGGUCCAGAUAGAUCGGCUCAUCACUGGUCGCCUCCAGAGUUUGCAGACGUACGUAACUCAGCAGCUCAUCCGAGCUGCUGAGAUACGUGCGUCUGCAAACCUGGCGGCGACCAAGAUGAGCGAGUGCGUGCUG<br>GGGCAGAGCAAGCGAGUGGACUUCUGCGGGAAGGGCUAUCACCUGAUGUCCUUCCCGCAGAGCGCACCUCACGGGGUAGUCUUCCUGCACGUGACAUAUGUGCCGGCGCAGGAGAAGAACUUCACGACUGCGCCGGCCAUA<br>UGUCACGACGGGAAGGCGCACUUCCCGCGUGAAGGAGUGUUCGUGUCAAACGGGACGCAUUGGUUCGUCACGCAGCGCAACUUCUAUGAGCCCCAGAUAAUUACCACUGACAAUACCUUUGUCAGUGGUAACUGCGACGUG<br>GUCAUAGGCAUUGUGAACAACACUGUCUAUGACCCGUUGCAGCCCGAGCUAGAUAGUUUCAAGGAGGAGCUUGAUAAGUACUUCAAGAAUCAUACUUCCCCAGAUGUGGAUCUUGGCGACAUUAGCGGGAUCAACGCUAGU<br>GUCGUCAACAUCCAGAAGGAGAUCGACAGGCUCAAUGAGGUUGCGAAGAACCUCAACGAGAGCCUGAUCGAUCUCCAGGAGCUGGGGAAGUAUGAGCAGUACAUCAAGUGGCCUUGGUACAUCUGGCUCGGGUUUAUCGCG<br>GGGCUCAUAGCGAUCGUCAUGGUGACGAUCAUGUUGUGCUGCAUGACGAGCUGUUGCUCCUGUUUGAAGGGCUGCUGCAGCUGCGGUUCGUGUUGUAAGUUUGACGAGGAUGACUCGGAGCCAGUGCUGAAGGGGGUCAAG<br>UUACACUACACG
SARS-CoV-2<br>SPIKE	Random Walk<br>-2512.31 kcal/mol<br>*-2486.70 kcal/mol*	AUGUUCGUGUUUCUUGUCCUCCUUCCACUGGUUUCGAGUCAGUGCGUCAAUCUUACAACACGAACCCAGCUGCCGCCAGCCUACACGAACUCCUUCACGCGGGGAGUGUACUACCCCGACAAGGUGUUCCGCUCGUCUGUU<br>CUGCACAGCACGCAGGACCUCUUCCUCCCGUUCUUCUCGAACGUGACAUGGUUCCAUGCCAUUCACGUUUCGGGAACGAACGGGACGAAGAGGUUCGACAACCCUGUUCUGCCGUUCAACGACGGGGUGUACUUCGCUUCG<br>ACAGAGAAGUCCAACAUUAUUCGCGGAUGGAUAUUCGGAACCACUCUCGAUUCCAAGACUCAGUCCUUGUUGAUAGUGAACAACGCCACGAACGUGGUCAUUAAGGUCUGUGAGUUUCAGUUCUGUAAUGACCCGUUCCUG<br>GGUGUUUACUAUCACAAGAACAACAAGUCUUGGAUGGAGAGUGAGUUCCGAGUGUAUUCAUCCGCGAAUAAUUGUACCUUCGAGUAUGUCAGUCAGCCGUUCCUGAUGGAUCUUGAAGGCAAGCAGGGCAAUUUCAAGAAU<br>UUGCGCGAGUUUGUCUUCAAGAACAUCGACGGCUACUUCAAGAUAUACUCGAAGCACACGCCAAUCAACCUCGUCCGUGAUCUCCCGCAGGGCUUCAGCGCUCUGGAGCCGCUGGUGGACUUGCCGAUAGGCAUCAACAUC<br>ACGCGGUUCCAGACGCUGCUGGCCCUGCACAGGAGUUACCUGACGCCAGGUGACUCCUCGUCCGGGUGGACUGCAGGUGCCGCCGCGUACUACGUGGGGUACCUGCAGCCCCGGACGUUCUUGUUGAAGUACAACGAGAAC<br>GGGACGAUCACGGACGCGGUUGAUUGCGCGUUGGACCCUCUGUCGGAGACGAAGUGCACCCUGAAGUCGUUUACGGUAGAAAAGGGGAUCUAUCAGACCUCCAACUUCCGCGUCCAGCCGACUGAGAGUAUCGUUCGGUUC<br>CCGAACAUCACGAACCUGUGUCCGUUCGGAGAGGUCUUCAACGCGACCAGGUUCGCCUCCGUGUACGCUUGGAACAGGAAGAGGAUCUCGAAUUGUGUAGCAGACUACAGUGUGCUAUACAAUUCGGCGUCCUUUUCCACU<br>UUCAAGUGUUACGGAGUGUCGCCCACGAAGUUGAACGACCUCUGCUUCACCAACGUGUAUGCGGAUUCCUUCGUCAUCCGUGGUGACGAAGUGCGUCAGAUUGCUCCGGGACAGACGGGUAAGAUAGCGGACUACAAUUAU<br>AAGUUGCCCGACGACUUUACUGGCUGCGUUAUUGCUUGGAAUAGCAAUAACUUAGACAGUAAAGUCGGGGGCAACUAUAACUAUCUUUACCGUCUGUUCCGGAAGAGCAAUCUGAAGCCCUUCGAGCGCGAUAUCUCGACC<br>GAAAUCUACCAGGCAGGCUCCACCCCGUGCAACGGGGUGGAGGGCUUCAAUUGUUACUUUCCGUUGCAGAGCUACGGAUUUCAGCCCACCAACGGGGUAGGGUACCAGCCCUACCGCGUGGUGGUGCUGAGUUUCGAGCUU<br>CUGCACGCCCCCGCGACUGUCUGUGGGCCCAAGAAGUCGACGAACUUGGUGAAGAACAAGUGCGUCAACUUCAACUUCAAUGGGCUCACAGGCACGGGGGUGUUGACGGAGAGUAACAAGAAGUUCCUGCCUUUCCAGCAG<br>UUCGGUCGAGAUAUCGCGGACACCACGGAUGCCGUAAGGGAUCCGCAGACGCUUGAGAUUCUGGACAUCACGCCCUGCAGCUUCGGGGGCGUCAGUGUAAUCACGCCUGGUACAAACACCAGCAACCAGGUUGCGGUGUUG<br>UACCAGGACGUGAAUUGCACUGAGGUCCCCGUAGCGAUCCACGCGGAUCAGCUAACCCCAACGUGGAGGGUGUACUCGACAGGGAGUAAUGUCUUCCAGACUCGCGCUGGUUGUCUGAUUGGCGCUGAGCAUGUGAACAAC<br>UCGUACGAGUGUGACAUCCCCAUUGGAGCGGGGAUCUGCGCGUCGUACCAGACCCAGACGAACAGCCCGAGGCGUGCCCGCUCAGUAGCGUCGCAGUCGAUCAUCGCGUACACGAUGAGCUUGGGGGCGGAGAACAGUGUA<br>GCCUAUUCGAACAACAGCAUUGCUAUCCCCACAAAUUUUACAAUUAGUGUAACCACCGAGAUCUUGCCGGUCUCGAUGACCAAGACCUCGGUGGAUUGCACUAUGUAUAUUUGUGGGGAUAGCACUGAGUGUUCUAAUCUC<br>CUGCUCCAGUACGGCAGUUUCUGUACGCAGCUCAACCGAGCGCUUACAGGAAUUGCCGUGGAGCAGGACAAGAACACUCAGGAGGUGUUUGCGCAGGUCAAGCAGAUCUACAAGACACCUCCGAUCAAGGACUUUGGCGGC<br>UUUAACUUCUCCCAGAUACUCCCCGACCCCAGCAAGCCCAGCAAGCGUAGCUUUAUUGAAGAUCUGCUCUUCAAUAAAGUUACGCUUGCUGAUGCUGGGUUCAUCAAGCAGUACGGGGACUGUCUGGGAGAUAUAGCCGCC<br>AGAGACUUGAUCUGUGCUCAGAAGUUCAAUGGGCUCACUGUUCUCCCCCCCUUGCUCACGGACGAGAUGAUCGCGCAGUACACUUCGGCGCUGCUGGCGGGCACGAUCACCUCGGGCUGGACGUUUGGGGCUGGUGCGGCG<br>CUGCAGAUCCCGUUCGCCAUGCAGAUGGCGUACCGCUUCAAUGGGAUCGGUGUCACACAGAACGUGUUGUACGAGAAUCAGAAGCUCAUCGCCAAUCAGUUCAACAGCGCGAUCGGGAAGAUACAGGACUCCCUGUCGAGU<br>ACAGCCUCCGCGUUGGGGAAGCUGCAGGACGUGGUGAACCAGAAUGCUCAAGCGUUGAACACGUUGGUGAAGCAGUUGUCGUCCAACUUCGGGGCGAUAAGUUCGGUGCUGAACGAUAUUCUCAGUCGGCUGGACAAGGUG<br>GAAGCGGAGGUCCAGAUAGAUCGGCUCAUCACUGGUCGCCUCCAGAGUUUGCAGACGUACGUAACUCAGCAGCUCAUCCGAGCUGCUGAGAUACGUGCGUCUGCAAACCUGGCGGCGACCAAGAUGAGCGAGUGCGUGCUG<br>GGGCAGAGCAAGCGAGUGGACUUCUGCGGGAAGGGCUAUCACCUGAUGUCCUUCCCGCAGAGCGCACCUCACGGGGUAGUCUUCCUGCACGUGACAUAUGUGCCGGCGCAGGAGAAGAACUUCACGACUGCGCCGGCCAUA<br>UGUCACGACGGGAAGGCGCACUUCCCGCGUGAAGGAGUGUUCGUGUCAAACGGGACGCAUUGGUUCGUCACGCAGCGCAACUUCUAUGAGCCCCAGAUAAUUACCACUGACAAUACCUUUGUCAGUGGUAACUGCGACGUG<br>GUCAUAGGCAUUGUGAACAACACUGUCUAUGACCCGUUGCAGCCCGAGCUAGAUAGUUUCAAGGAGGAGCUUGAUAAGUACUUCAAGAAUCAUACUUCCCCAGAUGUGGAUCUUGGCGACAUUAGCGGGAUCAACGCUAGU<br>GUCGUCAACAUCCAGAAGGAGAUCGACAGGCUCAAUGAGGUUGCGAAGAACCUCAACGAGAGCCUGAUCGAUCUCCAGGAGCUGGGGAAGUAUGAGCAGUACAUCAAGUGGCCUUGGUACAUCUGGCUCGGGUUUAUCGCG<br>GGGCUCAUAGCGAUCGUCAUGGUGACGAUCAUGUUGUGCUGCAUGACGAGCUGUUGCUCCUGUUUGAAGGGCUGCUGCAGCUGCGGUUCGUGUUGUAAGUUUGACGAGGAUGACUCGGAGCCAGUGCUGAAGGGGGUCAAG<br>UUACACUACACG

Continued on next page

Table S5 – continued from previous page

ID	Method	mRNA Design
	Ours<br>-2515.84 kcal/mol<br>*-2486.20 kcal/mol*	AUGUUUGUGUUUCUUGUCCUCCUUCCACUGGUUUCGAGUCAGUGCGUCAAUCUUACAACACGAACCCAGCUGCCGCCAGCCUACACGAACUCCUUCACGCGGGGAGUGUACUACCCCGAUAAGGUGUUCCGCUCGUCUGUU<br>CUGCACAGCACGCAGGACCUCUUCCUCCCGUUCUUCUCGAACGUGACAUGGUUUCAUGCCAUUCACGUUUCGGGAACGAACGGGACGAAGAGGUUCGAUAACCCUGUUCUGCCGUUUAACGACGGGGUGUACUUCGCUUCG<br>ACAGAGAAGUCCAACAUUAUUCGCGGAUGGAUAUUCGGAACCACUCUCGAUUCCAAGACUCAGUCCUUGUUGAUAGUGAACAACGCCACGAACGUGGUCAUUAAGGUCUGUGAAUUUCAGUUCUGUAAUGACCCGUUCUUG<br>GGUGUUUACUAUCACAAGAACAAUAAGUCUUGGAUGGAGAGUGAGUUCCGAGUGUAUUCAUCCGCGAAUAAUUGUACCUUCGAGUAUGUCAGUCAGCCGUUCUUGAUGGAUCUUGAAGGCAAACAGGGCAAUUUUAAGAAU<br>UUGCGCGAGUUUGUCUUCAAGAACAUCGACGGCUACUUCAAGAUAUACUCGAAGCACACGCCAAUCAACCUCGUCCGUGAUCUCCCGCAGGGCUUCAGCGCUCUGGAGCCGCUGGUGGAUUUGCCGAUAGGCAUAAACAUC<br>ACGCGGUUCCAGACGCUGUUAGCCCUGCACAGGAGUUACCUGACGCCAGGUGACUCCUCGUCCGGGUGGACUGCAGGUGCCGCCGCGUACUACGUGGGGUACCUGCAGCCCCGGACGUUCUUGUUGAAGUACAACGAGAAC<br>GGGACGAUCACGGACGCGGUUGAUUGCGCGUUGGACCCUCUGUCGGAGACGAAGUGCACCCUGAAGUCGUUUACGGUAGAAAAGGGGAUCUAUCAGACCUCCAACUUCCGCGUCCAGCCGACUGAGAGUAUCGUUCGGUUC<br>CCGAACAUCACGAACCUGUGUCCGUUUGGAGAAGUCUUCAACGCGACCAGGUUCGCCUCCGUGUACGCUUGGAAUAGGAAGAGGAUCUCGAAUUGUGUAGCAGACUAUAGUGUGCUAUACAAUUCGGCGUCCUUUUCCACU<br>UUCAAGUGUUACGGAGUGUCGCCCACGAAGUUGAACGACCUCUGCUUCACCAACGUGUAUGCGGAUUCCUUCGUCAUCCGUGGUGACGAGGUGCGUCAGAUUGCUCCGGGACAGACGGGUAAGAUAGCGGAUUAUAAUUAU<br>AAGUUGCCCGACGACUUUACUGGCUGCGUUAUUGCUUGGAAUAGCAAUAACUUAGACAGUAAAGUCGGGGGCAACUAUAACUAUCUUUACCGUCUGUUCCGGAAGAGCAAUCUGAAGCCCUUCGAACGCGAUAUCUCGACC<br>GAAAUCUAUCAGGCAGGCUCCACCCCGUGCAACGGGGUGGAGGGCUUCAAUUGUUACUUUCCGUUGCAGAGCUACGGAUUUCAGCCCACCAACGGGGUAGGGUACCAGCCCUACCGCGUGGUGGUGCUGAGUUUCGAGCUC<br>CUGCACGCCCCCGCGACUGUCUGUGGGCCCAAGAAGUCGACGAACUUGGUGAAGAACAAGUGCGUCAACUUCAACUUCAAUGGGCUCACAGGCACGGGGGUGUUGACGGAGAGUAACAAGAAGUUCCUGCCUUUUCAGCAG<br>UUCGGUCGAGAUAUCGCGGACACCACGGAUGCCGUAAGGGAUCCGCAGACGCUUGAGAUUCUGGACAUCACGCCCUGCAGCUUCGGGGGCGUCAGUGUAAUCACGCCUGGUACAAACACCAGCAACCAAGUUGCGGUGUUG<br>UACCAGGACGUGAAUUGCACUGAGGUCCCCGUAGCGAUCCACGCGGAUCAGCUAACCCCAACGUGGAGGGUGUACUCGACAGGGAGUAAUGUCUUCCAGACUCGCGCUGGUUGUCUGAUUGGCGCUGAGCAUGUGAAUAAC<br>UCGUACGAGUGUGACAUCCCCAUUGGAGCGGGGAUCUGCGCGUCGUACCAGACCCAGACGAACAGCCCGAGGCGUGCCCGCUCAGUAGCGUCGCAGUCGAUCAUCGCGUACACGAUGAGCUUGGGGGCGGAGAACAGUGUA<br>GCCUAUUCGAACAACAGCAUUGCUAUCCCCACAAAUUUUACAAUUAGUGUAACCACCGAGAUCUUGCCGGUCAGCAUGACCAAGACCUCGGUGGAUUGCACUAUGUAUAUUUGUGGGGAUAGCACUGAGUGUUCUAAUCUC<br>CUGCUCCAAUACGGCAGUUUCUGUACGCAGCUCAAUAGAGCGCUUACAGGAAUUGCCGUGGAGCAGGAUAAGAACACUCAGGAGGUGUUUGCGCAGGUCAAGCAGAUCUACAAGACACCUCCGAUCAAGGACUUUGGCGGC<br>UUUAAUUUCUCCCAGAUACUCCCCGACCCCAGCAAGCCCAGCAAGCGUAGCUUUAUUGAAGAUUUACUCUUCAAUAAAGUUACGCUUGCUGAUGCUGGGUUCAUCAAGCAGUACGGGGAUUGUCUGGGAGAUAUAGCCGCC<br>AGAGACUUGAUCUGUGCUCAGAAGUUCAAUGGGCUCACUGUUCUCCCCCCCUUGCUCACGGACGAGAUGAUCGCUCAGUACACUUCGGCGCUGCUGGCGGGCACGAUCACCUCGGGCUGGACGUUUGGGGCUGGUGCGGCG<br>CUGCAGAUCCCGUUCGCCAUGCAGAUGGCGUACCGCUUCAAUGGGAUCGGUGUCACACAGAACGUCUUGUACGAGAAUCAGAAGCUCAUCGCCAAUCAGUUCAACAGCGCGAUCGGGAAGAUACAGGACUCCCUGUCGAGU<br>ACAGCCUCCGCGUUGGGGAAGCUGCAGGACGUGGUGAACCAGAAUGCUCAAGCGUUGAACACGUUGGUGAAGCAGUUGUCGUCCAACUUCGGGGCGAUAAGUUCGGUGCUGAACGAUAUUCUCAGUCGGCUGGACAAGGUG<br>GAAGCGGAGGUCCAGAUAGAUCGGCUCAUCACUGGUCGCCUCCAGAGUUUGCAGACGUACGUAACUCAGCAGCUCAUCCGAGCUGCUGAGAUACGUGCGUCUGCAAACCUGGCGGCGACCAAGAUGAGCGAGUGCGUGCUG<br>GGGCAGAGCAAGCGAGUGGACUUCUGCGGGAAGGGCUAUCAUUUGAUGUCCUUCCCGCAGAGUGCACCUCACGGGGUAGUCUUCCUGCACGUGACAUAUGUGCCGGCGCAGGAGAAAAAUUUUACGACUGCGCCGGCCAUA<br>UGUCACGACGGGAAGGCGCACUUCCCGCGUGAAGGAGUGUUCGUGUCAAACGGGACGCAUUGGUUCGUCACGCAGCGCAACUUCUAUGAGCCCCAGAUAAUUACCACUGACAAUACCUUUGUCAGUGGUAACUGCGACGUG<br>GUCAUAGGCAUUGUGAACAACACUGUCUAUGACCCGUUGCAGCCCGAGCUAGAUAGUUUCAAGGAAGAGCUUGAUAAGUACUUCAAGAAUCAUACUUCCCCAGAUGUGGAUCUUGGCGACAUUAGCGGGAUCAACGCUAGU<br>GUCGUCAAUAUCCAGAAGGAGAUCGACAGGCUCAAUGAGGUUGCGAAGAACCUCAAUGAGAGCCUGAUCGAUCUCCAGGAGCUGGGGAAGUAUGAGCAGUACAUCAAGUGGCCUUGGUACAUCUGGCUCGGGUUUAUCGCG<br>GGGCUCAUAGCGAUCGUCAUGGUGACGAUCAUGUUGUGCUGCAUGACGAGCUGUUGCUCCUGUUUGAAGGGCUGCUGCAGCUGCGGUUCGUGUUGUAAGUUUGACGAGGAUGACUCGGAGCCAGUGCUGAAGGGGGUCAAG<br>UUACACUACACA

Appendix D: Tightness and Optimality of Surrogate Objective

Equivalence at integral solutions.
Let $\delta_{\boldsymbol{x}^*}$ be a one-hot distribution that puts all its probability mass on a single sequence $\boldsymbol{x}^*$. Because the expectation collapses,

$$\mathbb{E}_{\boldsymbol{x}\sim\delta_{\boldsymbol{x}^*}(\cdot)}\big[\log Q(\boldsymbol{x})\big] \;=\; \log Q(\boldsymbol{x}^*), \quad \mathbb{E}_{\boldsymbol{x}\sim\delta_{\boldsymbol{x}^*}(\cdot)}\big[Q(\boldsymbol{x})\big] \;=\; Q(\boldsymbol{x}^*),$$

and therefore $\widetilde{\Delta G^\circ_{\text{ens}}}(\delta_{\boldsymbol{x}^*}) = \overline{\Delta G^\circ_{\text{ens}}}(\delta_{\boldsymbol{x}^*}) = -RT\log Q(\boldsymbol{x}^*)$.

Local optimality at integral solutions.
Let $\mathbb{D}$ be the probability simplex over sequences. We define

$$c(\boldsymbol{x}) \;\triangleq\; -RT\log Q(\boldsymbol{x}), \qquad \tilde{c}(\boldsymbol{x}) \;\triangleq\; Q(\boldsymbol{x}).$$

For the original EFE objective we have

$$\widetilde{\Delta G^\circ_{\text{ens}}}(\mathbb{D}) \;=\; \sum_{\boldsymbol{x}} p(\boldsymbol{x})\, c(\boldsymbol{x}).$$

If a local optimum is achieved at some non-integral solution (i.e. interior point) $\mathbb{D}$ where two sequences $\boldsymbol{x}_1, \boldsymbol{x}_2$ satisfy $p(\boldsymbol{x}_1), p(\boldsymbol{x}_2) > 0$ and $c(\boldsymbol{x}_1) < c(\boldsymbol{x}_2)$. We choose an infinitesimal $\varepsilon > 0$ small enough from $\boldsymbol{x}_2$ to $\boldsymbol{x}_1$ that $p'(\boldsymbol{x}_1) = p(\boldsymbol{x}_1) + \varepsilon \le 1$ and $p'(\boldsymbol{x}_2) = p(\boldsymbol{x}_2) - \varepsilon \ge 0$. Keep all other probabilities unchanged, so it give us a new distribution $\mathbb{D}$'. We have

$$\widetilde{\Delta G^\circ_{\text{ens}}}(\mathbb{D}') - \widetilde{\Delta G^\circ_{\text{ens}}}(\mathbb{D}) \;=\; \big[p(\boldsymbol{x}_1)+\varepsilon\big]c(\boldsymbol{x}_1) + \big[p(\boldsymbol{x}_2)-\varepsilon\big]c(\boldsymbol{x}_2) - \Big[p(\boldsymbol{x}_1)c(\boldsymbol{x}_1) + p(\boldsymbol{x}_2)c(\boldsymbol{x}_2)\Big] \;=\; \varepsilon\big[c(\boldsymbol{x}_1) - c(\boldsymbol{x}_2)\big].$$

Since $c(\boldsymbol{x}_1) < c(\boldsymbol{x}_2)$ by assumption, $c(\boldsymbol{x}_1) - c(\boldsymbol{x}_2) < 0$, so

$$\widetilde{\Delta G^\circ_{\text{ens}}}(\mathbb{D}') - \widetilde{\Delta G^\circ_{\text{ens}}}(\mathbb{D}) < 0 \quad\Longrightarrow\quad \widetilde{\Delta G^\circ_{\text{ens}}}(\mathbb{D}') < \widetilde{\Delta G^\circ_{\text{ens}}}(\mathbb{D}).$$

which contradicts with the local optimality of $\widetilde{\Delta G^\circ_{\text{ens}}}(\mathbb{D})$. Thus any locally optimal $\mathbb{D}$ must put its entire mass on sequences with the *lowest* $c(\boldsymbol{x})$, i.e. on a single $\boldsymbol{x}^*$ whenever the minimum is unique.

On the other hand, the surrogate can be rewritten as

$$\overline{\Delta G^\circ_{\text{ens}}}(\mathbb{D}) \;=\; -RT\log\Big(\sum_{\boldsymbol{x}} p(\boldsymbol{x})\,\tilde{c}(\boldsymbol{x})\Big),$$

and the logarithm is strictly *monotone*. Hence minimizing $\overline{\Delta G^\circ_{\text{ens}}}$ is equivalent to minimizing the *linear* form $\sum p(\boldsymbol{x})\tilde{c}(\boldsymbol{x})$, so the same simplex argument shows that *every local (and global) minimizer of* $\overline{\Delta G^\circ_{\text{ens}}}$ *is also a one-hot distribution.* When the minimum of $\tilde{c}$ is unique, both objectives select the *same* integral solution $\delta_{\boldsymbol{x}^*}$; when it is tied, each objective admits the *set* of one-hot distributions on the tied sequences as local optima, so integral solutions still suffice.

Why the surrogate is usually tight.
The Jensen gap is

$$\widetilde{\Delta G^\circ_{\text{ens}}}(\mathbb{D}) - \overline{\Delta G^\circ_{\text{ens}}}(\mathbb{D}) = RT\Big(\log \mathbb{E}_{\boldsymbol{x}\sim\mathbb{D}(\cdot)}[Q(\boldsymbol{x})] - \mathbb{E}_{\boldsymbol{x}\sim\mathbb{D}(\cdot)}[\log Q(\boldsymbol{x})]\Big).$$

Using a second-order Taylor expansion of log around the mean $\mu = \mathbb{E}_{\boldsymbol{x}\sim\mathbb{D}(\cdot)}[Q(\boldsymbol{x})]$, one obtains

$$\log Q(\boldsymbol{x}) \approx \log\mu \;+\; \frac{Q(\boldsymbol{x})-\mu}{\mu} \;-\; \frac{(Q(\boldsymbol{x})-\mu)^2}{2\mu^2},$$

so the gap is approximately

$$\frac{RT}{2\mu^2}\,\mathbb{E}_{\boldsymbol{x}\sim\mathbb{D}(\cdot)}[(Q(\boldsymbol{x})-\mu)^2] = \frac{RT}{2\mu^2}\,\mathrm{Var}_{\boldsymbol{x}\sim\mathbb{D}(\cdot)}[Q(\boldsymbol{x})].$$

When the $Q(\boldsymbol{x})$ values are large, μ is large and the coefficient $1/\mu^2$ shrinks rapidly (the slope of log is $1/\mu$), making the gap empirically negligible.
